# Development of an improved RT-qPCR Assay for detection of *Japanese encephalitis virus* (JEV) RNA including a systematic review and comprehensive comparison with published methods

**DOI:** 10.1371/journal.pone.0194412

**Published:** 2018-03-23

**Authors:** Tehmina Bharucha, Onanong Sengvilaipaseuth, Manivanh Vongsouvath, Malavanh Vongsouvath, Viengmon Davong, Phonepasith Panyanouvong, Géraldine Piorkowski, Jeremy A. Garson, Paul N. Newton, Xavier de Lamballerie, Audrey Dubot-Pérès

**Affiliations:** 1 Lao-Oxford-Mahosot Hospital-Wellcome Trust Research Unit (LOMWRU), Microbiology Laboratory, Mahosot Hospital, Vientiane, Lao P.D.R; 2 Division of Infection and Immunity, University College London, London, United Kingdom; 3 Departments of Infectious Diseases and Microbiology, Royal Free Hospital, London, United Kingdom; 4 UMR "Unité des Virus Emergents" (UVE: Aix-Marseille Univ–IRD 190 –Inserm 1207 –IHU Méditerranée Infection), Marseille, France; 5 National Transfusion Microbiology Laboratories, NHS Blood and Transplant, London, United Kingdom; 6 Centre for Tropical Medicine and Global Health, Nuffield Department of Clinical Medicine, University of Oxford, Churchill Hospital, Oxford, United Kingdom; 7 London School of Hygiene and Tropical Medicine (LSHTM), London, United Kingdom; Consejo Superior de Investigaciones Cientificas, SPAIN

## Abstract

**Background:**

*Japanese encephalitis virus* (JEV) is a major cause of encephalitis in Asia, and the commonest cause of mosquito-borne encephalitis worldwide. Detection of JEV RNA remains challenging due to the characteristic brief and low viraemia, with 0–25% of patients positive, and the mainstay of diagnosis remains detection of anti-JEV IgM antibody.

**Methods:**

We performed a systematic review of published RT-PCR protocols, and evaluated them *in silico* and *in vitro* alongside new primers and probes designed using a multiple genome alignment of all JEV strains >9,000nt from GenBank, downloaded from the NCBI website (November 2016). The new assays included pan-genotype and genotype specific assays targeting genotypes 1 and 3.

**Results:**

Ten RT-qPCR assays were compared, a pre-existing in-house assay, three published assays and six newly designed assays, using serial RNA dilutions. We selected three assays, one published and two novel assays, with the lowest limit of detection (LOD) for further optimisation and validation. One of the novel assays, detecting NS2A, showed the best results, with LOD approximately 4 copies/ reaction, and no cross-reaction on testing closely related viruses in the JEV serocomplex, *West Nile Virus* and *St*. *Louis Virus*. The optimised assays were validated in consecutive patients with central nervous system infections admitted to hospitals in Laos, testing paired CSF and serum samples.

**Conclusions:**

We succeeded in developing a JEV specific RT-qPCR assay with at least 1 log_10_ improved sensitivity as compared to existing assays. Further evaluation is required, field-testing the assay in a larger group of patients.

## Introduction

*Japanese encephalitis virus* (JEV) represents a major cause of encephalitis in Asia, with an estimated 70,000 cases per year, and 20,000 deaths [1–5]. It is a highly neurotropic arthropod-borne virus (arbovirus), a member of the genus *Flavivirus* and family *Flaviviridae*, and closely related to *West Nile virus* (WNV) and *St*. *Louis encephalitis virus* (SLEV).

Five JEV genotypes have been identified, G1-5, with at least 12% nucleotide difference between them, and concomitant geographical variations. G1 and G3 have been isolated and characterised most extensively in humans [6–14]. G3 was the main genotype identified in Asia until the 1990s. However, there has been a recognised genotype displacement to G1 which is now predominant [10, 15]. G1 is the only genotype that has been isolated in Laos, but it is likely that G3 co-circulates as well [6, 16].

JEV is transmitted between birds and swine in enzootic cycles with mosquito vectors (mainly *Culex* spp.) [17]. Humans are ‘dead-end’ hosts, infected when they encroach upon enzootic cycles, mainly involving transmission from swine, which live close to humans and have high and prolonged viraemias. The viraemia elicited in humans is typically brief and low, and in endemic areas only a small proportion of those infected (1 in 300) experience symptoms [18–20].

There are recognised limitations in current diagnostics for JEV [21–24]. The mainstay of diagnosis remains the detection of anti-JEV IgM antibody using IgM antibody capture ELISA (JEV MAC-ELISA) from blood and/or cerebrospinal fluid (CSF) samples [25–27]. However cross-reactivity of *Flavivirus* immune-assays is well-documented, as seen with *Dengue virus* infection [28]. Moreover, recent dynamics in *Flavivirus* epidemiology, seen with the epidemic of *Zika virus*, further questions our dependence on detection of anti-JEV IgM [28–31]. Field studies suggest the sensitivity of JEV MAC-ELISA is only 50–93%, and more recently the positive predictive value has also been questioned [22, 23, 32, 33]. Additionally, the use of serology for diagnostic confirmation limits knowledge of molecular epidemiology and vaccine efficacy.

Reasons for reliance on JEV MAC-ELISA are the short period of JEV viraemia in humans and potential low sensitivity of existing JEV RT-PCR techniques. Data suggest that JEV RNA is detected in blood and/or CSF in a very small proportion (0–25%) of clinical cases [32, 34]. Studies reporting higher proportions, 25–30%, involve selected patient populations, such as patients with less than 3 days onset of fever and more severe symptoms [35, 36].

We aimed to perform a systematic review of the JEV RT-PCR assays available and to compare their performance. In addition, we developed new assays, in order to obtain the highest efficiency. This required extensive sequence analysis to design an *in-silico* optimal system followed by optimization of the experimental protocol. A set of selected assays were then assessed on patient samples.

## Material and methods

### Systematic literature review

MEDLINE, EMBASE and Web of Science databases were searched for MESH and keywords ‘*Japanese encephalitis*’ and ‘Polymerase Chain Reaction or $ $ $PCR’ upto 31^st^ January 2017. References and citations of selected studies were also identified using Web of Science, and hand-searched in bibliographies. Inclusion criteria involved English language studies reporting validation of RT-PCR methods for detection of JEV. Panflavivirus assays were not included unless this was part of a multiplex including a specific probe for JEV.

### Bioinformatic analysis, primer and probe design

JEV sequences over 9,000 nucleotides (nt) in length, identified in GenBank, were downloaded in FASTA format from the NCBI website on 12^th^ November 2016, and aligned using MAFFT version 7 [37]. The pre-existing in-house assay (Unité des Virus Emergents, Marseille, 2014) and published RT-qPCR assays were evaluated for best fit using the sequence alignment uploaded to MEGA 7 [38]. New primers and probes were designed in areas with maximal conservation, with a GC clamp (one or more Gs or Cs within the last 5 bases from the 3’ end), Tm of probes 10°C higher than the primers, checking for hairpins, self-dimers, heterodimers using the online IDTNA ‘oligoanalyzer tool’ 3.1 [39, 40]. The published and newly designed assays included TaqMan® Minor Groove Binder (MGB) probes. The sequences were also submitted to nucleotide BLAST search on NCBI web server to ensure high specificity of the sequences for JEV, and predicted products were folded using the M-fold web server to check for secondary structures affecting the performance of the RT-qPCR [41, 42].

### RNA standards

RNA from three JEV isolates were used for the optimisation process. Two G1 strains from Laos, JEV/CNS1326/Laos/2013 (no titre available) and JEV/CNS769/Laos/2009 (1.3x10^6^ RNA copies/μl; GenBank KC196115, EVA 001V-02217); and one G3 strain, UVE/JEV/UNK/TW/RP9-190 (1.2 x10^7^ RNA copies/μl; GenBank KF907505, EVA 001V-02344) [16]. Other *Flavivirus* RNAs from the JEV complex: two WNV strains, UVE/WNV/1999/US/NY 385–99 (10^3.57^ TCDI50/mL, GenBank AY842931, EVA 001v-EVA140) and UVE/WNV/UNK/CF/Ar B 3573/82 (10^4.07^ TCID50/mL, GenBank DQ318020, EVA 001v-EVA91), and one SLEV strain: UVE/SLEV/UNK/US/MSI-7 (10^4.82^ TCID50/mL, GenBank DQ359217, EVA 001v-EVA128). Serial 1:10 dilutions of each RNA were prepared using AVE buffer containing RNA carrier (Qiagen, UK) [43]. Working aliquots were frozen at -20° C to avoid repeat freeze-thawing. JEV RNA dilutions were selected to cover a wide range of concentrations from positive to negative for all assays and conditions tested. For other Flaviviruses RNA, 10^−5^ dilution was chosen because it represented a moderate Cq value of approximately 30 cycles in WNV and SLEV specific qPCR assays.

### Technical development of RT-qPCR assays

#### Comparison of RT-qPCR conditions using the pre-existing in-house system

Different factors were evaluated following step-by-step variation using the pre-existing in-house assay with dilutions of JEV G1-1326 RNA. Factors tested included: 1) Mastermix: EXPRESS One-Step SuperScript® qRT-PCR ‘Express kit’ (Thermo Fisher, UK), SuperScript™ III One-Step RT-PCR System with Platinum™ Taq DNA Polymerase ‘Superscript-III kit’ (Thermo Fisher, UK) and TaqMan® Fast Virus 1-Step ‘Fastvirus kit’ (Thermo Fisher, UK), according to manufacturers’ instructions, see standard reaction mix preparation and cycling conditions in Table 1; 2) Reaction volumes (25 vs 50μL); 3) Sample volumes (5–16μL), see Table 2. All RT-qPCR assays for optimisation and subsequent experiments were run using a CFX96^TM^ qPCR detection system (Biorad laboratories). Cq > 40 were reported as negative, and ≤40 as positive.

**Table 1:** Standard reaction mixes and cycling conditions.

Mastermix	Preparation	Cycling Conditions
**Superscript-III kit** SuperScript™ III One-Step RT-PCR System with Platinum™ Taq DNA Polymerase	Total Reaction volume 25 μL: 1) x2 Mastermix 12.5 μL 2) Forward and reverse primers 400nM 3) Probe 160nM 4) Enzyme 0.5 μL 5) JEV RNA 5 μL	50°C for 15 minutes 95°C for 2 minutes X 45 (95°C for 15 seconds + 60°C for 45 seconds)
**Express kit** EXPRESS One-Step SuperScript® qRT-PCR	Total Reaction volume 20 μL: 1) x2 Mastermix 10 μL 2) Forward and reverse primers 400nM 3) Probe 160nM 4) Enzyme 2 μL 5) JEV RNA 5 μL	50°C for 15 minutes 95°C for 2 minutes 45 x (95°C for 15 seconds + 60°C for 45 seconds)
**Fastvirus kit** TaqMan® Fast Virus 1-Step	Total Reaction volume 25 μL: 1) X4 Mastermix 6.25 μL 2) Forward and reverse primers 400nM 3) Probe 160nM 4) JEV RNA 5 μL	50°C for 5 minutes 95°C for 20 seconds 45 x (95°C for 15 seconds + 60°C for 60 seconds)

**Table 2:** Comparison of RT-qPCR conditions using the pre-existing in-house RT-qPCR assay*.

Factor	Mastermix^#^	Sample volume	Reaction volume	Annealing temperature	Primer concentration	Probe concentration
I Mastermix	1. Superscript-III 2. Express 3. Fastvirus	5μl	1. 25μl 2. 20μl 3. 25μl	60°C	400nM	160nM
II Reaction volume	Superscript-III	1. 5μl 2. 10μl	1. 25μl 2. 50μl	60°C	400nM	160nM
III Sample volume	Superscript-III	1. 10μl 2. 16μl	50μl	60°C	400nM	160nM
	Fastvirus	1. 5μl 2. 15μl	25μl	60°C	400nM	160nM

* All experiments were performed with G1-1326 RNA tenfold dilutions.

^#^Superscript-III kit: SuperScript™ III One-Step RT-PCR System with Platinum™ Taq DNA Polymerase; Express kit: EXPRESS One-Step SuperScript® qRT-PCR and Fastvirus kit: TaqMan® Fast Virus 1-Step.

#### Selection of the best performing RT-qPCR systems

The ten different systems were compared by testing them on 7 tenfold dilutions of JEV G1-1326 RNA in duplicate, and G3 RP9-190 RNA tested once, using ‘Superscript-III kit’ following standard conditions, see Table 1. On the basis of these results, the three RT-qPCR systems with the lowest limit of detection (LOD), i.e. highest dilution found positive for all replicates, were selected for optimisation.

#### Assay optimisation

The three selected RT-qPCR assays were performed on dilutions of JEV G1-1326 RNA in duplicate using ‘Superscript-III kit’ and step-by-step optimisation of various conditions, involving 1) annealing temperatures 54–64°C, 2) primer concentration (200-800nM), and 3) probe concentration (100-400nM), see Table 3.

**Table 3:** Optimisation experiments performed for the three best performing RT-qPCR systems*.

Factor	Mastermix	Sample volume	Reaction volume	Annealing temperature^#^	Primer concentration	Probe concentration
I Annealing temperature	Superscript-III	5μl	25μl	54, 56, 58, 60, 62 and 64°C	400nM	200nM
II Primer concentration	Superscript-III	5μl	25μl	1. 60°C 2. 62°C 3. 56°C	200, 300, 400, 500, 600 and 800 nM	200nM
III Probe concentration	Superscript-III	5μl	25μl	1. 60°C 2. 62°C 3. 56°C	600nM	100, 200, 300 and 400nM

* All experiments were performed with G1-1326 tenfold dilutions.

^#^Annealing temperature for the Pyke, NS2A and NS3 systems were 60, 62 and 56°C respectively.

### Validation

For the three selected RT-qPCR assays, efficiency, repeatability and LOD, were assessed using optimised RT-qPCR assay protocols (based on results of 2.4.1 and 2.4.3) using JEV G1-769 or JEV G3- RP9-190 RNA dilutions (10^−3^ to 10^−10^) in triplicate, and repeated on two different days. Cross-reaction of the assays were tested using RNA from viruses in the JEV serocomplex at 10^−5^ dilutions: two WNV strains and one SLEV strain.

### Evaluation using patient samples with Central Nervous System (CNS) infections

Two groups of consecutive patients with CNS infection, admitted 2009–2015, were retrospectively tested: group 1 with acute encephalitis syndrome determined using the clinical WHO criteria [44] and admitted within 7 days of fever onset to Mahosot Hospital; and group 2 with suspected CNS infection from three other hospitals in Vientiane, the Friendship, Children and Setthathirat Hospitals. CSF was collected by lumbar puncture (LP) from consenting patients without contraindications to LP, according to the judgment of the responsible physician. Blood was collected on admission for all patients. Samples were sent to the Mahosot Hospital Microbiology Laboratory. CSF and serum, after blood centrifugation, were aliquoted and stored at -80°C.

200 μl serum and CSF samples were extracted and eluted in 60μl on a EZ1 Advanced machine using EZ1 Virus Mini Kit v2.0 (Qiagen, UK) following manufacturer’s instructions. The validated JEV RT-qPCR assays were performed as per the optimised protocols for each sample. Positive and negative (no template) controls were performed for each RT-qPCR run. An internal control (MS2 phage) was added to each patient sample. After extraction, MS2 RT-qPCR was performed to control the extraction process and to exclude inhibition as previously described [45].

### Sequencing

Single positive RT-qPCR results were further investigated by sending the RT-qPCR product for next-generation sequencing using Ion S5 system (Thermo Fisher, Waltham, USA), to Unité des Virus Emergents, Faculty of Medicine, Marseille, France.

### Pan-flavivirus RT-PCR

The standard diagnostic algorithm at Mahosot Hospital did not include a JEV RT-PCR assay, but it did include prospective testing using a pan-flavivirus hemi-nested SybrGreen RT-qPCR with sequencing for identification. This was performed as per a published protocol [46], with results presented alongside JEV RT-qPCR results.

### Panbio ELISA

Detection of anti-JEV IgM using JEV MAC-ELISA is the World Health Organisation recommended test for diagnosing JEV infection. Japanese Encephalitis-Dengue IgM Combo ELISA kit (Catalogue number E-JED01C; Panbio ELISA; Inverness Medical Innovations, Brisbane, Australia, formerly Panbio Ltd) is a commercial JEV MAC-ELISA kit that is combined with an anti-Dengue IgM test to exclude cross-reactivity. The test was performed according to the manufacturers’ instructions, reported as anti-JEV or anti-Dengue IgM Positive, Equivocal or Negative [22]. For the purposes of this analysis, anti-Dengue IgM Positive or Equivocal, or anti-JEV IgM Equivocal were interpreted as anti-JEV IgM Negative.

### Ethical considerations

The study was part of an ongoing study on the causes of CNS infections in Laos. Ethical clearance was granted by the Ethical Review Committee of the Faculty of Medical Sciences, National University of Laos, and the Oxford University Tropical Ethics Research Committee, Oxford, UK. Diagnostic testing was performed on anonymised and frozen serum and CSF samples previously collected. Venepuncture and LPs were performed if written consent was given by patients or their parents/guardian.

## Results

### Systematic review and bioinformatics analysis

Forty-six studies evaluating JEV RT-PCR assays were identified (see S1 File, S1 Fig and Table A in S2 File), including 7 assays utilising hydrolysis probes [27, 47–52]. The first published record of JEV RT-PCR was performed as a conventional RT-PCR at the National Institute of Health, Maryland, USA, in 1991 [53]. Since then, a variety of techniques have been harnessed: conventional and nested RT-PCR; and RT-qPCR using hydrolysis probes or SYBR green. Genotype-specific assays have also been reported, specifically for G1 and G3 [52, 54].

Thirteen (28%) studies reported detection of JEV RNA in patients (see Table A in S2 File). Among these studies, the median number of published JEV cases confirmed by the assay was 12 (range 1–75). These mainly involved CSF samples, but the assays also detected JEV RNA in serum. A case report has recently demonstrated detection of JEV RNA in urine, although this was not substantiated by a larger case series of 52 JEV patients [55, 56].

The primers and probes from the five published hydrolysis probe RT-qPCR assays specifically designed for detection of JEV RNA in human cases (Table C in S2 File) were aligned with the 303 complete genome sequence (see Table B in S2 File for Accession numbers, Table D in S2 File for sequence alignment). Pyke *et al*, Yang *et al* and Shirato *et al* [27, 48, 50] systems showed the best fit with the sequence alignment *in-silico*, with published validation data including genotypes tested, and were chosen for subsequent analysis. Six new primer/probe sets were also designed, targeting conserved regions of 1) all Genotypes, 2) G1 and G3 (Table 4). It is notable that the G1 and G3 assays were designed using two separate multiple genome alignments containing only strains belonging to the corresponding genotype. However, they were not exclusively targeting a specific genotype, and for this reason the assays were tested against both G1 and G3.

**Table 4:** Primers and probes evaluated during the study.

Author	Target (nt)	Oligonucleotides	Sequence (5’-3’)*	Tm (°C)
Pre-existing in-house	NS3 (103)	Forward	6399-TYG-AYG-CRA-GRG-TTT-ATG-CAG	61–68
		Probe 1	Fam-AGT-GGT-TTA-AGG-ATT-TTG-CAG-C-Tamra	62
		Probe 2	Fam-AGT-GGT-TCA-ARG-ACT-TTG-CAG-C-Tamra	64–66
		Reverse	6502-CAT-RCG-ACC-GAG-CAC-CTC-TA	64–67
Pyke *et al* 2004	NS5 (62)	Forward	10230-ATC-TGG-TGY-GGY-AGT-CTC-A	61–67
		Probe	Fam-CGG-AAC-GCG-ATC-CAG-GGC-AA-Tamra	69
		Reverse	10292-CGC-GTA-GAT-GTT-CTC-AGC-CC	65
Yang *et al* 2004	3' UTR (146)	Forward	10764-GGT-GTA-AGG-ACT-AGA-GGT-TAG-AGG	64
		Probe	Fam-CCC-GTG-GAA-ACA-ACA-TCA-TGC-GGC-Tamra	70
		Reverse	10910-ATT-CCC-AGG-TGT-CAA-TAT-GCT-GTT	66
Shirato *et al* 2005	5' UTR (75)	Forward	80-AGA-ACG-GAA-GAY-AAC-CAT-GAC-TAA-A	64–66
		Probe	Fam-ACC-AGG-AGG-GCC-CGG-MGB NFQ	81
		Reverse	155-CCG-CGT-TTC-AGC-ATA-TTG-AT	62
**New (this study)**				
NS2A (all Genotypes)–v1	NS2A (116)	Forward	3563-AGC-TGG-GCC-TTC-TGG-T	64
		Probe	Fam-CTT-CGC-AAG-AGG-TGG-ACG-GCC-A-Tamra	70
		Reverse	3675-CCC-AAG-CAT-CAG-CAC-AAG	62
NS2A)–v2	NS2A (112)	Forward	3563-AGC-TGG-GCC-TTC-TGG-T	64
		Probe	Fam- TGG-CCG-TCC-ACC-TCT-TGC-GAA-G -Tamra	70
		Reverse	3675-CCC-AAG-CAT-CAG-CAC-AAG	62
NS5 (G 1)–v1	NS5 (315)	Forward	9925-GDG-CTG-GAT-GGA-ATG-TGA	61–63
		Probe	Fam-AGG-AGA-GTG-GAT-GAC-CAC-MGB NFQ	75
		Reverse	10240-CCA-CAC-CAG-ATG-TCC-TC	60
NS5 (G 1)–v2	NS5 (315)	Forward	9925-GDG-CTG-GAT-GGA-ATG-TGA	61–63
		Probe	Fam-AGG-AGA-GTG-GAT-GAC-YAC-MGB NFQ	72–76
		Reverse	10240-CCA-CAC-CAG-ATG-TCC-TC	60
NS3 (G 3)–v1	NS3 (141)	Forward	5726-GCA-ATG-TGC-CTC-CAA-AGA-GC	65
		Probe	Fam-TCC-TAT-GAY-ACA-GAA-TAY-CCA-AA-MGB NFQ	73–78
		Reverse	5884-GTC-GAT-GAC-CCT-GCT-CGC	66
NS3 (G 3)–v2	NS3 (158)	Forward	5726-GCA-ATG-TGY-CTC-CAA-AGA-GC	63–66
		Probe	Fam-TCC-TAT-GAY-ACA-GAA-TAY-CCA-AA-MGB NFQ	73–78
		Reverse	5884-GTC-GAT-GAC-CCT-GCT-CGC	66

*Position of the oligonucleotides is related to the Nakayama strain, Accession number EF571853.

Tm: melting temperature calculated using IDTNA tool https://www.idtdna.com/calc/analyzer setting parameters as follows: Target type RNA, Oligo Conc 0.4μM, Na Conc 50mM, Mg Conc 3mM, dNTPs Conc 0.8mM. Tms for MGB (minor groove binder) probes include an additional 15°C.

A more extensive list of all oligonucleotides evaluated *in-silico* is included in Table C in S2 File.

NS2Av2 was designed as NS2Av1 with a modification of the probe sequence as its reverse complement.

NS5v2 was designed as NS5v1 with a modification of the probe sequence, with a degeneracy Y inserted instead of C at position 16.

NS3v2 was designed as NS3v1 with a modification of the forward primer sequence, with a degeneracy Y inserted instead of C at position 9.

NS = gene coding for non-structural protein. G = genotype

### Technical development of RT-qPCR assays

#### Comparison of RT-qPCR conditions using the pre-existing in-house system

Experiments were performed to establish the best mastermix kits, reaction and sample volumes using the pre-existing in-house system. All the possible combinations were not performed, but the kits were evaluated with 5 tenfold dilutions of G1-1326 RNA in duplicate or triplicate to adequately compare the limits of detection.

#### Mastermix

Three kits were compared, Superscript-III kit, Express kit and Fastvirus kit. The LOD for Superscript-III kit and Fastvirus kit was 10^−6^, as compared to 10^−5^ for Express kit (Table E in S2 File). The Express kit was not therefore used in further experiments.

#### Reaction volumes (25μl vs 50μl)

The manufacturers’ instructions for both Superscript-III and Fastvirus kits advise performing RT-qPCR as 50μl reaction volumes. In an effort to conserve reagents, only Superscript-III kit was used to compare two reaction volumes, 25μl and 50μl. Increasing the reaction volume from 25μl to 50μl resulted in a lower LOD, 10^−6^ vs 10^−7^ (Table E in S2 File).

#### Sample volumes

The standard sample volume was 5μl. As suggested by the kit manufacturer, the sample volume may be increased by not adding water in the reaction mix. For Superscript-III kit, the sample volume may be increased to 8μl in a 25μl reaction volume, equivalent to 16μl in a 50μl reaction volume. For Fastvirus kit the sample volume may be increased to 15μl in a 25μl reaction volume, vs. 30μl in a 50μl reaction volume. The results demonstrate a lower LOD (10^−7^ vs. 10^−6^) with a larger sample volume (Table E in S2 File).

The experiments thus demonstrated that the optimal conditions involved use of the Fastvirus kit with a 30μl sample volume and 50μl reaction volume. These optimised conditions were subsequently used for the validation experiments described in Section 3.3.

#### Selection of the best performing RT-qPCR systems

A total of ten RT-qPCR systems, the pre-existing in-house, three published systems, and six (two versions each of three) newly designed systems, were tested on JEV G1-1326 RNA in duplicate, and G3 RP9-190 RNA once, in tenfold serial dilutions using Superscript-III under standard conditions. RT-qPCR results are presented in Table F in S2 File. Three RT-qPCR systems with the lowest LOD were selected for further optimisation: two pan-genotype assays, the Pyke and NS2A version 1 (subsequently called NS2A); and a G3 assay, NS3 version 2 (subsequently called NS3).

#### Assay optimisation

The Tm, primer and probe concentrations were optimised using Superscript-III under standard reaction and sample volumes.

The performance of the three selected assays at different annealing temperatures from 52 to 64°C was assessed by testing JEV G1-1326 RNA in triplicate in tenfold serial dilutions. A three-step protocol was used for the cycling: 1) denaturation 95°C for 0:15, 2) annealing x°C for 0:30, and then 3) extension 68°C for 0:30. Adjusting the annealing temperature of the thermocycling protocol improved the LOD by one log_10_ for two assays, Cq results presented in Table G in S2 File. The optimal annealing temperature for the Pyke assay was 60°C, NS2A assay was 62°C and NS3 assay was 56°C (Table G in S2 File).

The assays were tested in duplicate at various combinations of forward and reverse primers concentrations, from 200-600nM, using G1-1326 RNA at 10^−6^, results presented in Table H in S2 File. There was minimal difference between Cq results, and a concentration of 600nM for both forward and reverse primers were selected for further experiments.

The assays were tested at different probe concentrations, from 100-400nM, using G1-1326 RNA in duplicate at 10^−6^, results presented in Table I in S2 File. 300nM was selected for further experiments.

Note that Cq values in Tables H and Table I in S2 File are not directly comparable to each other because different PCR runs employed different threshold settings.

#### Validation

In view of the results detailed above, the final conditions employed Fastvirus kit with a reaction volume of 50μL, sample volume of 30μl, and primer and probe concentrations of 600nM and 300nM, respectively. The optimal annealing temperature was different for each assay: 60°C, 62°C and 56°C for the Pyke, NS2A and NS3 assays, respectively. This was performed as a two-step protocol, as per the Fastvirus manufacturer’s instructions.

**Fig 1:**
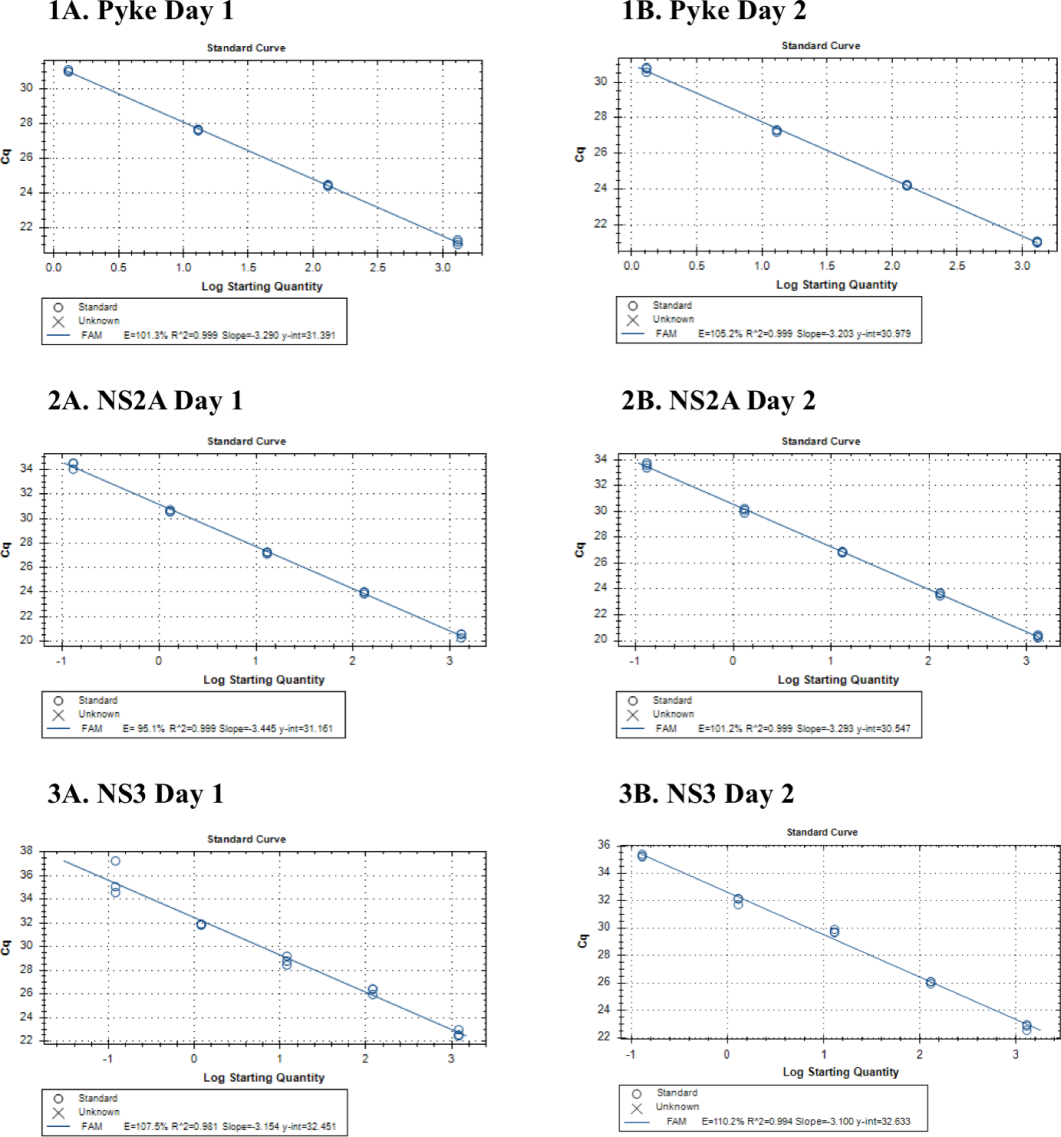
Standard curves of the 1) Pyke, 2) NS2A assays and 3) NS3 assays with G3-RP9-190 on (A) Day 1 and repeated on (B) Day 2. Result of the RT-qPCR run, ‘Cq’, is plotted against the ‘log starting copies number’, at the RNA dilutions detected: Pyke assay 1:10 serial dilutions of G1-769 in triplicate at 10^−3^ to 10^−6^; NS2A assay 1:10 serial dilutions of G1-769 in triplicate at 10^−3^ to 10^−7^; and NS3 with G3-RP-190 10^−4^ to 10^−7^. Efficiency = 10^−1/slope^-1. R^2^ = Correlation Coefficient. RT-qPCR performed with Fastvirus kit (TaqMan® Fast Virus 1-Step) with a reaction volume of 50μL, sample volume of 30μl, and primer and probe concentrations of 600nM and 300nM respectively. Thermocycling conditions were 50°C for 5 minutes, 95°C for 20 seconds and 45 x (95°C for 15 seconds + x°C for 60 seconds). The optimal annealing temperature ‘x°C’ was different for each assay: 62°C, 60°C and 56°C for the Pyke, NS2A and NS3 assays respectively.

RT-qPCR was performed with serial RNA dilutions in triplicate on 2 different days, for the Pyke and the NS2A assays using G1-769 10^−3^ to 10^−10^ or for NS3 (G3 assay) with G3-RP-190 10^−4^ to 10^−11^. The standard curves are shown in Fig 1 with Cq results in Table 5. The limit of detection (LOD) for the Pyke assay was 1.3 JEV RNA copies/μl or 39 copies/reaction, for the NS2A assay was 1.3x10^-1^ JEV RNA copies/μl or ~4 copies/reaction and for the NS3 assay was 1.2 x10^-1^ JEV RNA copies/μl or ~4 copies/reaction. Similar results were obtained when testing NS2A on G3-RP9-190, see Table 5.

**Table 5:** Cq results for validation of the optimised JEV RT-qPCR assays.

Assay	JEV Strain	Day	N	10^−3^			10^−4^			10^−5^			10^−6^			10^−7^			10^−8^			10^−9^			10^−10^			10^−11^		
				Cq	Mean	SD	Cq	Mean	SD	Cq	Mean	SD	Cq	Mean	SD	Cq	Mean	SD	Cq	Mean	SD	Cq	Mean	SD	Cq	Mean	SD	Cq	Mean	SD
Pyke	G1	1	1	21.30	21.16	0.14	24.45	24.44	0.05	27.66	27.65	0.05	31.14	31.06	0.07	39.28	No Cq	-	No Cq	No Cq	-	No Cq	No Cq	-	No Cq	No Cq	-			
Pyke	G1	1	2	21.17			24.39			27.69			31.03			35.24			No Cq			No Cq			No Cq					
Pyke	G1	1	3	21.02			24.49			27.60			31.02			No Cq			No Cq			No Cq			No Cq					
Pyke	G1	2	1	21.00	21.05	0.05	24.20	24.22	0.04	27.19	27.27	0.07	30.55	30.71	0.14	34.27	35.95	2.52	No Cq	No Cq	-	No Cq	No Cq	-	No Cq	No Cq	-			
Pyke	G1	2	2	21.05			24.27			27.30			30.75			38.84			No Cq			No Cq			No Cq					
Pyke	G1	2	3	21.09			24.19			27.32			30.82			34.73			No Cq			No Cq			No Cq					
NS2A	G1	1	1	20.57	20.47	0.18	24.00	23.96	0.10	27.10	27.21	0.10	30.58	30.62	0.09	34.03	34.36	0.29	36.98	No Cq	-	No Cq	No Cq	-	No Cq	No Cq	-			
NS2A	G1	1	2	20.57			23.85			27.29			30.72			34.51			No Cq			No Cq			No Cq					
NS2A	G1	1	3	20.26			24.04			27.24			30.56			34.54			36.27			No Cq			No Cq					
NS2A	G1	2	1	20.44	20.33	0.12	23.46	23.60	0.13	26.91	26.84	0.06	30.09	30.06	0.18	33.58	33.57	0.19	39.83	37.60	1.99	No Cq	No Cq	-	No Cq	No Cq	-			
NS2A	G1	2	2	20.33			23.71			26.82			29.87			33.37			36.93			No Cq			No Cq					
NS2A	G1	2	3	20.21			23.64			26.80			30.22			33.75			36.03			No Cq			No Cq					
NS2A	G3	1	1													30.51	30.65	0.20	34.06	34.18	0.17	No Cq	No Cq	-	No Cq	No Cq	-			
NS2A	G3	1	2													30.79			34.30			39.67			No Cq					
NS3	G3	1	1				22.46	22.66	0.28	25.96	26.27	0.27	28.78	28.81	0.38	31.85	31.87	0.04	34.57	35.63	1.43	38.49	No Cq	-	No Cq	No Cq	-	No Cq	No Cq	-
NS3	G3	1	2				22.98			26.42			28.45			31.84			37.25			38.25			No Cq			No Cq		
NS3	G3	1	3				22.55			26.42			29.20			31.92			35.07			No Cq			No Cq			No Cq		
NS3	G3	2	1				22.95	22.78	0.21	26.08	26.04	0.09	29.72	29.77	0.13	31.72	32.01	0.25	35.22	35.30	0.10	39.85	No Cq	-	No Cq	No Cq	-	No Cq	No Cq	-
NS3	G3	2	2				22.85			25.94			29.68			32.18			35.27			No Cq			No Cq			No Cq		
NS3	G3	2	2				22.54			26.10			29.92			32.12			35.41			No Cq			No Cq			No Cq		

Dilution series performed using JEV G1 RNA (JEV/CNS769/Laos/2009; 1.3x10^6^ RNA copies/μl; GenBank: KC196115, EVA 001V-02217) and JEV G3 RNA (UVE/JEV/UNK/TW/RP9-190; 1.2 x10^7^ RNA copies/μl; GenBank KF907505, EVA 001V-02344) with triplicates on 2 different days. N = replicate number. LOD (highest dilution found positive by all replicates) is shaded in grey. RT-qPCR performed with Fastvirus kit (TaqMan® Fast Virus 1-Step) with a reaction volume of 50μL, sample volume of 30μl, and primer and probe concentrations of 600nM and 300nM respectively. Thermocycling conditions were 50°C for 5 minutes, 95°C for 20 seconds and 45 x (95°C for 15 seconds + x°C for 60 seconds). The optimal annealing temperature ‘x°C’ was different for each assay: 62°C, 60°C and 56°C for the Pyke, NS2A and NS3 assays respectively.

Efficiency was calculated from the standard curve for Day 1 and 2: 101.3% and 105.2% for the Pyke; 95.1% and 101.2% for NS2A; and 107.5% and 110.2% for NS3 (Fig 1). Assay repeatability was assessed by calculating the standard deviation (SD) for the Cq variance, presented in Table 5. SD for all assays was less than 0.40, excluding the dilution at the LOD, suggesting adequate repeatability of the assays.

No amplification was detected in testing the assays with WNV or SLEV.

### Evaluation using patient samples with Central Nervous System (CNS) infections

123 patients were tested with the three optimised RT-qPCR assays (conditions as described in Section 3.2.1), 58 patients from group 1 (2011–2014) and 65 from group 2 (2008–2015), including a total of 95 serum and 120 CSF samples. 35.8% (44/123) of the patients were found anti-JEV IgM positive by MAC ELISA. Positive RT-qPCR results are presented in Table 6, alongside results for JEV MAC-ELISA, hemi-nested panflavivirus RT-qPCR and RT-qPCR sequencing results. None of the samples were considered inhibitory according to the results of MS2 testing. Two patients (2 CSF samples) were positive using the NS2A assay, of which 1 (1 CSF) was also positive by the NS3 assay. These 2 patients were known positives by cell inoculation and/or hemi-nested panflavivirus assay. 12 (8 CSF and 4 serum samples) were positive by the Pyke assay alone. As 11/12 (92%) of these patients were not previously detected by the pan-flavivirus assay or cell inoculation, the RT-qPCR products were sent for sequencing. This revealed that the amplicons generated by the Pyke assay with these clinical samples consisted of primer multimers and primer/probe concatemers resulting from non-specific amplification, possibly related to the unusually high GC content at 3’ end of the reverse primer, GCCC.

**Table 6:** Evaluation of the three optimised RT-qPCR assays using patient samples with Central Nervous System (CNS) infections. Patients with RT-qPCR ‘positive’ test results.

Number	Age	Sex	Serology Results (PU)*			JEV RT-qPCR Results (Cq)**						Pan-flavivirus nested RT-qPCR		Sequencing Results
			Admission Serum JEV IgM	Convalescent Serum JEV IgM	CSF JEV IgM	Serum			CSF					
						Pyke	NS2A	NS3	Pyke	NS2A	NS3	CSF	Serum	
1	17	F	Positive(15.3)	Positive(56.5)	Positive(32.8)	ND	ND	ND	Negative	Positive(36)	Positive(37)	Negative	Negative	JEV sequence identified^◆^
2	42	F	Negative (8.6)	ND	Negative (2.9)	Positive(31)	Negative	Negative	Negative	Negative	Negative	Negative	Negative	Non-specific amplification
3	60	M	Negative (3.9)	ND	Negative (3.5)	Negative	Negative	Negative	Positive(31)	Negative	Negative	Negative	Negative	Non-specific amplification
4	60	M	Negative (3.6)	ND	Negative (3.6)	Positive(40)	Negative	Negative	Negative	Negative	Negative	ND	ND	Non-specific amplification
5	58	F	Positive(15.9)	ND	Negative (3.4)	Negative	Negative	Negative	Positive(34)	Negative	Negative	ND	ND	Non-specific amplification
6	-	-	Positive(54.6)	ND	Positive(80.8)	Negative	Negative	Negative	Positive(40)	Negative	Negative	Negative	Negative	Non-specific amplification
7	-	M	Negative (2.5)	ND	Negative (2.6)	Negative	Negative	Negative	Positive(32)	Negative	ND	ND	ND	Non-specific amplification
8	12	M	Negative (6.8)	ND	Positive(57.2)	Negative	Negative	Negative	Positive(39)	Positive(35)	Negative	Positive	ND	JEV sequence identified^◆^
9	26	M	Negative (5.8)	ND	Negative (3.3)	Negative	Negative	-	Positive(36)	Negative	Negative	ND	ND	Non-specific amplification
10	27	M	Negative (4.6)	ND	Negative (3.6)	Positive(28)	Negative	Negative	Negative	Negative	Negative	Negative	Negative	Non-specific amplification
11	33	M	Negative (3.2)	ND	Negative (2.8)	Positive(40)	Negative	Negative	Negative	Negative	Negative	ND	ND	Non-specific amplification
12			Negative (6.4)	ND	Negative (4.9)	Negative	Negative	Negative	Positive(36)	Negative	Negative	ND	ND	Non-specific amplification
13	-	-	Positive(27.8)	ND	Positive(29.9)	Negative	Negative	Negative	Positive(32)	Negative	Negative	Negative	Negative	Non-specific amplification

* JEV-Dengue IgM Combo ELISA kit (Panbio ELISA), Inverness Medical Innovations, Brisbane, Australia (formerly Panbio Ltd.). PU = Panbio Unit, calculated from OD according to manufacturer’s instruction. JEV IgM Positive, Negative or Equivocal cut-offs calculated according to the manufacturer’s instructions.

** Pyke, NS2A and NS3 JEV RT-qPCR assays were reported as negative if the Cq>40 or not detected.

^◆^ JEV sequence identified as part of a previous study, for Patient 1 by sequencing following cell culture, and for Patient 2 by sequencing of the hemi-nested qPCR product.

ND Not done.

## Discussion

This systematic review confirmed that the detection of JEV RNA in suspected human cases by RT-PCR is uncommon. Forty-six studies were identified, involving a variety of RT-PCR techniques, however only a quarter had any published record of detection of JEV RNA in human samples. It was common for an article title to poorly identify the study as evaluating a JEV RT-PCR method, and while every effort was made to hand-search publications, it is possible that the search was not 100% comprehensive. Additionally, studies utilising the assays may not have cited them.

Articles rarely adhered to the MIQE guidelines for evaluation of RT-PCR methods, such as reporting accession numbers of JEV strains used or details of the optimisation process [39]. Many were only optimised and validated on G3, whereas G1 is now the predominant strain. It is also important to differentiate studies developing RT-qPCR for detection in mosquitos as opposed to humans, as they are not consistently interchangeable. Mosquito surveillance may require a cost-effective and rapid method, but in clinical diagnostics analytical accuracy is of utmost important. To this end, a number of studies detecting JEV RNA in clinical samples incorporated nested RT-PCR methods. Although nested RT-PCR may improve both analytical sensitivity and specificity, it is cumbersome and prone to cross-contamination. In the study by Meiyu *et al* [57], it was reported that out of 52 clinical JEV cases, an extraordinary 45 (87%) were PCR positive for JEV RNA. However, there was no additional evidence to support the diagnosis, such as repeat testing, sequencing of the isolates or alternative confirmation. Existing RT-qPCR assays detect JEV RNA in 0–25% of clinical cases.

It is for these reasons that we focussed on identifying and validating the most effective RT-qPCR assay. Hydrolysis probe assays are the most extensively used methods for nucleic acid detection, both for clinical and research diagnostic purposes. From the results of the systematic review, 3 published systems incorporating hydrolysis probes were selected on the basis of best matching *in silico*. Assessment with JEV RNA dilutions of G1 and G3 showed the lowest LOD was obtained with the Pyke system: 2 logfold below both the Yang and Shirato assays with G1, and 1 logfold below both with G3 [27, 50]. It is recognised that the conditions employed were not identical to those published, and it would have been interesting to compare the validated assays with existing assays performed with published conditions. Validation experiments confirmed good RT-qPCR efficiency and LOD of 1.3 JEV RNA copies/μl or 39 copies/reaction. However, in a study of patient samples with paired serum and CSF, all the positive RT-qPCR products that were sequenced, even results with Cq of approximately 30 showed non-specific amplification. Eleven positive samples were detected by the Pyke assay alone, of which all were sequenced and showed non-specific amplification.

The results highlight the importance of validating a newly designed RT-qPCR method against a panel of negative patients samples in addition to the no-template controls recommended by the MIQE guidelines [39]. The oligonucleotides tested in these experiments had been checked *in-silico* for specificity, and also experimentally tested for cross-reactivity against strains of the closely related viruses, WNV and SLE. All the experiments had been performed with at least one no template control per RT-qPCR plate. Nonetheless, the results of validation against patient CSF and serum samples suggested poor specificity of the Pyke assay. It is possible that further optimisation, increasing the annealing temperature and reducing the primer concentrations may have improved the specificity, although this may adversely affect sensitivity of the assay.

The new assays aimed to improve the sensitivity of detection, through targeting various conserved regions from a multiple genome alignment. The NS2A region was chosen for the pan-genotype assay, and NS5 and NS3 for the G1 and G3 systems, respectively. Two versions of each system were designed. An initial assessment showed poor performance of the NS5 assay, and this was not used in further experiments. Initial optimisation experiments demonstrated best results with Fastvirus mix, with the maximal reaction and sample volumes (although it is recognised that use of 30 μL of extract for a single assay may not be consistently possible in practice). The assays were individually optimised for annealing temperature, primer and probe concentrations. Validation showed one logfold lower LOD for the NS2A compared to the published Pyke assay for G1, NS2A assay was 1.3x10^-1^ JEV RNA copies/μl or ~4 copies/reaction. NS2A and NS3 showed equally good results for G3, with LOD 1.2 x10^-1^ JEV RNA copies/μl or ~4 copies/reaction. Individual assays targeting highly conserved regions of G1 and G3 did not improve the sensitivity of diagnosis. Nonetheless, it may be useful to target multiple regions of the viral genome to overcome false negatives due to mutations in any single region.

A study of patient samples, including paired serum and CSF, demonstrated marginally improved detection of JEV RNA as compared to the existing hemi-nested panflavivirus RT-qPCR assay. One case was detected with the hemi-nested RT-qPCR assay, and both this and an additional case were detected by the new assays. The low number of positives is consistent with previous data. It must also be taken into account that this involved retrospective testing using samples stored for many years. The potential greater sensitivity of the NS2A as compared to the NS3 is likely due to both JEV strains detected established previously by sequencing as G1.

In conclusion, the novel pan-genotype NS2A assay and G3 NS3 assay described here showed higher analytical sensitivity than previously described RT-qPCR assays for JEV RNA. However, it will be essential to field-test them in serum, CSF and other body fluids, to evaluate their sensitivity and potential for improved diagnostic capacity in a large prospective study. Since our systematic review, there have been no further JEV specific diagnostic assays published, but there has been evidence of the potential for improved detection of JEV RNA in other body fluids, whole blood [56], throat swabs (Bharucha *et al*, submitted) and urine samples [56, 58], and this needs further evaluation.

## Supporting information

S1 Fig PRISMA flow diagram. (DOC)

S1 File PRISMA checklist. (DOC)

S2 File Table A: Overview of JEV-specific RT-PCR assays* and Sample Types Used to Detect Human JEV cases. Table B: Accession numbers of all included sequences. Table C: Complete list of Primers and Probes Evaluated *In-Silico*. Table D: Sequence alignment of Primers and Probes. Table E: Cq Results for Comparison of RT-qPCR conditions using the Pre-existing In-house JEV RT-qPCR assay. Table F: Cq Results for the Selection of the Best Performing RT-qPCR systems Using Superscript-III kit with Standard Conditions. Table G: Cq Results for Annealing Temperature Optimisation using the Pre-existing In-house JEV RT-qPCR assay. Table H: Cq Results for Primer Concentration Optimisation Experiments Using Superscript-III kit with Standard Conditions. Table I: Cq Results for Probe Concentrations Optimisation Experiments Using Superscript-III kit with Standard Conditions.
(ZIP)
