# Supplementary material for: Development of an improved RT-qPCR Assay for detection of Japanese encephalitis virus (JEV) RNA including a systematic review and comprehensive comparison with published methods
Source: PLoS One. 2018 Mar 23;13(3):e0194412. doi: 10.1371/journal.pone.0194412 (PMC5865736; doi:10.1371/journal.pone.0194412)
Supplement: S1 Fig — (DOC) [file pone.0194412.s001.doc]

S1 Fig: PRISMA Flow Diagram.

**Screening**

**Included**

**Eligibility**

**Identification**

Records identified through database searching
(n = 976)

Additional records identified through other sources
(n = 22)

Records after duplicates removed
(n = 656)

Titles & Abstracts screened
(n = 656)

Records excluded
(n = 579)

Full-text articles assessed for eligibility
(n = 77)

Full-text articles excluded (non-English language/ not reporting JEV RT-PCR validation study/ panflavivirus assay)

(n = 31)

Studies included in qualitative synthesis
(n = 46)
