# Supplementary material for: Development of an improved RT-qPCR Assay for detection of Japanese encephalitis virus (JEV) RNA including a systematic review and comprehensive comparison with published methods
Source: PLoS One. 2018 Mar 23;13(3):e0194412. doi: 10.1371/journal.pone.0194412 (PMC5865736; doi:10.1371/journal.pone.0194412)
Supplement: S2 File — Table A: Overview of JEV-specific RT-PCR assays* and Sample Types Used to Detect Human JEV cases. Table B: Accession numbers of all included sequences. Table C: Complete list of Primers and Probes Evaluated In-Silico. Table D: Sequence alignment of Primers and Probes. Table E: Cq Results for Comparison of RT-qPCR conditions using the Pre-existing In-house JEV RT-qPCR assay. Table F: Cq Results for the Selection of the Best Performing RT-qPCR systems Using Superscript-III kit with Standard Conditions. Table G: Cq Results for Annealing Temperature Optimisation using the Pre-existing In-house JEV RT-qPCR assay. Table H: Cq Results for Primer Concentration Optimisation Experiments Using Superscript-III kit with Standard Conditions. Table I: Cq Results for Probe Concentrations Optimisation Experiments Using Superscript-III kit with Standard Conditions. (ZIP) [file pone.0194412.s003.zip › S2 File Tables A-I.docx]

**Supplementary Data**

**Table A. Overview of JEV-specific RT-PCR assays* and Sample Types Used to Detect Human JEV cases.**

|  |  |  |  |  |  |  | **Studies using the assay on human cases** | | |
| --- | --- | --- | --- | --- | --- | --- | --- | --- | --- |
| **RT-PCR** | **Ref-erence** | **RT-PCR target**** | **RT-PCR technique** | **Amplicon size (nt)** | **JEV Genotype Analytical** | **JEV Genotype Field** | **Number of positive** | **Sample types** | **Ref** |
| Barros, 2013 | (46) | NS2A | RT-qPCR with Hydrolysis Probe | 136 | G3 | - | None published | - | (59) |
| Chao 2007 | (60) | NS5 | Multiplex Flavivirus RT-qPCR with Hydrolysis probes | 261 | G3 | G1, G4 | None published | - | (61, 62) |
| Chen, 2011 | (63) | E | Conventional and Nested RT-PCR (and RT-LAMP^$^) | 221 | G3 | - | None published | - |  |
| Chen, 2014 | (51) | 1) C/PrM (U=Universal),  2) PrM/E (G3) 3) E/NS1 (G1) | Mulitplex JEV RT-qPCR with JEV pan-genotype (U), G1 and G3 specific Hydrolysis probes | 1) 570 (U)  2) 395 (G3)  3) 708 (G1) | G1, G3 | G1, G3 | None published | - |  |
| Deng, 2015 | (53) | E | Conventional RT-PCR (and RT-LAMP combined with a Lateral Flow Assay) | 200 | G3 | - | None published | - |  |
| Dhanze, 2015 | (64) | NS1 | Conventional RT-PCR, and RT-qPCR (and SYBR Green RT-LAMP) | 220 | G3 | Not reported | None published | - |  |
| Dong, 2012 | (65) | C | Multiplex RT-qPCR | 192 | G1 | Not reported | 29 | CSF | (65) |
| Eldadah, 1991 | (50) | E | Conventional RT-PCR and confirmed by restriction endonuclease cleavage. | 350 | G3 | - | None published | - |  |
| Huang, 2004 | (48) | NS3 | RT-qPCR with Hydrolysis probe | 96 | G1, G2, G3 | - | None published | - | (54) |
| Huang, 2008 | (66) | E | Conventional RT-PCR, RT-qPCR, SYBR Green, with and without gold naonparticles | 306 | G3 | - | None published | - |  |
| Jeong, 2011 | (67) | 1) 5’UTR/PrM  2) C | Nested Conventional RT-PCR | 1) 517 2) 227 | G1, G3 | G1 | None published | - |  |
| Kabilan, 1999 | (68) | NS5 | Conventional RT-PCR | 546 | - | Not reported | 11 | CSF | (68) |
| Kong, 2015 | (69) | E | Multiplex Flavivirus Conventional RT-PCR | 429 | - | Not reported | 11 | CSF | (69) |
| Kuwayama, 2005 | (70) | E | Nested Conventional RT-PCR*** | 326 | G3 | G3 | 4 | CSF | (70) |
| Lindahl, 2013 | (71) | 1) NS5/3’UTR  2) NS5 | Nested Conventional RT-PCR. Inner oligonucleotides based on previously published assay with one degeneracy (47). | 1) 650  2) 62 | G3 | G1, G3 | None published | - |  |
| Liu, 2009 | (72) | 1) PrM  2) 5’UTR/C/PrM | Conventional RT-PCR (and RT-LAMP) | 1) 186  2) 868 | G3 | - | None published |  |  |
| Liu, 2012 | (73) | E | Conventional RT-PCR and RT-qPCR (and RT-LAMP) | 1) 475(G1) 2) 524 (G3) | G1, G3 | G1, G3 | None published | - |  |
| Ma, 2017 | (74) | E | Conventional RT-PCR (and RT-LAMP with ZnO nanomaterials) | 185 | G3 | - | None published |  |  |
| Meiyu, 1997 | (56) | NS1 | Nested Conventional RT-PCR | 1) 413  2) 323 | G3 | Not reported | 45 | Serum | (56) |
| Murakami, 1994 | (75) | E | Nested Conventional RT-PCR | 1) 241  2) 181 | G3 | - | None published | - |  |
| Ogawa, 2009 | (76) | PrM/E | Multiplex Conventional RT-PCR | 605 | G3 | Not reported | None published | - |  |
| Paranjpe, 1998 | (77) | E | Nested RT-PCR | 488 | G1 | Not reported | None published | - |  |
| Parida, 2006 | (78) | E | Conventional RT-PCR (and RT-LAMP) | 235 | G3 | Not reported | 13 | CSF | (78) |
| Patel, 2013 | (79) | NS5 | Multiplex Flavivirus RT-qPCR with Locked Nucleic Acid Hydrolysis Probes | 260 | G3 | - | None published | - |  |
| Pham, 2017 | (80) | E | Multiplex Conventional RT-PCR for 10 Viruses causing Encephalitis: HHV6, Influenza A virus, Parechovirus, HSV1&2, JEV, Rotavirus, Enterovirus, Adenovirus, and Dengue virus | 326 | G1, G3 | Not reported | None published | - |  |
| Pierre, 1994 | (81) | NS5 | Flavivirus RT-qPCR primers with specific hybridisation probes | 564 | G3 | - | None published | - |  |
| Pyke, 2004 | (47) | NS5 | RT-qPCR with Hydrolysis probe | 62 | G1, G2 | G1-3 | None published | - | (71, 82-85) |
| Raengsakulrach, 1999 | (86) | NS3 | Nested Conventional RT-PCR | 191 | G1 | - | # | CSF and Serum | (87, 88) |
| Santhosh, 2007 | (36) | NS3 | RT-qPCR with SYBR Green | 162 | G1 | G1, G3 | 18 | CSF | (36, 63) |
| Sapkal, 2007 | (89) | C/PrM | Conventional RT-PCR | 346 | G3 | G3 | 2 | Blood Clots | (89, 90) |
| Sarkar, 2012 | (91) | E | Conventional RT-PCR | 1500 | G3 | G3 | 75 | CSF and Serum | (91-93) |
| Saxena, 2009 | (57) | E | Conventional RT-PCR | 237 | G3 | Not reported | 28 | Serum | (57) |
| Shirato, 2005 | (49) | 5’UTR | RT-qPCR with a Multiplex MGB hydrolysis probe to detect both JEV and WNV | 75 | G1, G3 | G1 | 1 | Urine | (55, 94) |
| Swami, 2008 | (26) | PrM/E | Nested Conventional RT-PCR | 488 | G3 | Not reported | 11 | CSF and Post-mortem brain tissue | (26) |
| Tanaka, 1993 | (95) | NS3 | RT-qPCR with specific hybridisation probe | 142 | G3 | Not reported | None published | - | (59) |
| Tian, 2012 | (96) | NS3 | Conventional RT-PCR (and RT-LAMP with SYBR Green) | 162 | G3 | Not reported | None published | - |  |
| Tiawsirisup, 2010 | (97) | C/PrM | Conventional RT-PCR | 681 | G3 | Not reported | None published | - |  |
| Toriniwa, 2006 | (98) | E | Conventional RT-PCR (and RT-LAMP and confirmed by restriction endonuclease cleavage) | 250 | G3 | Not reported | None published | - | (99) |
| Wang, 2007 | (100) | 1) PrM  2) E | Conventional RT-PCR | 1) 675  2) 582 | G1, G3 | G1, G3 | None published | # | (54) |
| Van Den Hurk 2014 | (101) | 3’UTR | RT-qPCR with a MGB hydrolysis probe | 81 | - | - | None published | - |  |
| Yang, 2004 | (27) | 5’UTR, PrM, 3’UTR | RT-qPCR with hydrolysis probes | 1) 125  2) 65  3) 146 | G1, G3 | G1, G3 | None published | - | (102, 103) |
| Yang, 2016 | (52) | E | Multiplex Conventional RT-PCR for U, G1 and G3 | 1) 306 (U)  2) 565 (G1)  3) 451 (G3) | G1, G3 | - | None published | - |  |
| Yeh, 2010 | (104) | NS3 | Duplex Conventional RT-PCR for JEV and WNV | 241 | G1, G3 |  |  |  |  |
| Zeng, 2014 | (105) | E | Multiplex Conventional RT-PCR for swine viruses: Pseudorabies virus, Porcine parvovirus, Porcine circovirus type 2; Porcine reproductive and respi-ratory syndrome virus, Classical swine fever virus, and JEV. | 1015 | Not reported | Not reported | None published | - |  |
| Zhang, 2001 | (106) |  | Long Conventional RT-PCR for sequencing |  |  |  |  |  |  |
| Zhang, 2015 | (107) | C | Conventional RT-PCR and Nested Conventional RT-PCR (and RT-LAMP) | G1, G3 | 341  342 | Not reported | None published | - |  |

*Panflavivirus RT-PCR assays are not presented unless they involve a multiplex or nested technique with a specific JEV probe.

**The JEV genome encodes for 3 structural proteins, capsid (C), pre-membrane/membrane (PrM/M) and envelope (E); and 7 non-structural proteins, NS2A, NS2B, NS3, NS4A, NS4B and NS5. There are also 5’ and 3’ Untranslated Regions (UTR).

***The Nested RT-PCR had been previously reported but the publication was not included in the review as it was published in Japanese (108).

# No positive out of human samples tested.

$ Although RT-LAMP is not a RT-PCR method, it is reported in this table in brackets for publications that include both RT-LAMP to RT-PCR methods.

Abbreviations used in the table in order of first appearance: RT-PCR assay – Reverse transcription Polymerase Chain Reaction assay; qPCR – Real-time PCR; JEV – *Japanese encephalitis virus*; nt – nucleotides; G1 – genotype 1; G3 – genotype 3; U – Universal/Pan-genotype; CSF – cerebrospinal fluid; WNV – West Nile virus.

**Table B. Accession numbers of all included sequences.**

| **Number** | **Genotype** | **Accession Number** |
| --- | --- | --- |
| 1 | 1 | AB241118.1 |
| 2 | 1 | KU351667.1 |
| 3 | 1 | JN381849.1 |
| 4 | 1 | JF706274.1 |
| 5 | 1 | GQ902062.1 |
| 6 | 1 | KM658163.1 |
| 7 | 1 | KU508409.1 |
| 8 | 1 | KU351668.1 |
| 9 | 1 | KU508408.1 |
| 10 | 1 | KF667316.1 |
| 11 | 1 | JF706286.1 |
| 12 | 1 | HQ893545.1 |
| 13 | 1 | JF706271.1 |
| 14 | 1 | JF706270.1 |
| 15 | 1 | JN381850.1 |
| 16 | 1 | JN381852.1 |
| 17 | 1 | KT957420.1 |
| 18 | 1 | KT229574.1 |
| 19 | 1 | KT229575.1 |
| 20 | 1 | JN381844.1 |
| 21 | 1 | JN381845.1 |
| 22 | 1 | GU556217.1 |
| 23 | 1 | KX357114.1 |
| 24 | 1 | EU880214.1 |
| 25 | 1 | AB830335.1 |
| 26 | 1 | AB981183.1 |
| 27 | 1 | AB981184.1 |
| 28 | 1 | EU429297.1 |
| 29 | 1 | EU693899.1 |
| 30 | 1 | JF499788.1 |
| 31 | 1 | KF667325.1 |
| 32 | 1 | KF667321.1 |
| 33 | 1 | KF667322.1 |
| 34 | 1 | JF499789.1 |
| 35 | 1 | KF667317.1 |
| 36 | 1 | AB594829.1 |
| 37 | 1 | JF706268.1 |
| 38 | 1 | JN381833.1 |
| 39 | 1 | KC196115.1 |
| 40 | 1 | KF667323.1 |
| 41 | 1 | KF667324.1 |
| 42 | 1 | KF667326.1 |
| 43 | 1 | AB471666.2 |
| 44 | 1 | GQ902058.1 |
| 45 | 1 | GQ902059.1 |
| 46 | 1 | AB920399.1 |
| 47 | 1 | KT229572.1 |
| 48 | 1 | KT957419.1 |
| 49 | 1 | KT229573.1 |
| 50 | 1 | JF706267.1 |
| 51 | 1 | JN381836.1 |
| 52 | 1 | JN381835.1 |
| 53 | 1 | JQ031753.1 |
| 54 | 1 | JF499790.1 |
| 55 | 1 | KF667319.1 |
| 56 | 1 | KF667320.1 |
| 57 | 1 | KF667318.1 |
| 58 | 1 | KF667327.1 |
| 59 | 1 | AB471667.2 |
| 60 | 1 | AB698907.1 |
| 61 | 1 | AB698908.1 |
| 62 | 1 | AB853904.1 |
| 63 | 1 | AB698909.1 |
| 64 | 1 | JN381837.1 |
| 65 | 1 | JN381841.1 |
| 66 | 1 | AB698905.1 |
| 67 | 1 | AB698906.1 |
| 68 | 1 | JN381842.1 |
| 69 | 1 | HM366552.1 |
| 70 | 1 | GQ902060.1 |
| 71 | 1 | AY316157.1 |
| 72 | 1 | JN381848.1 |
| 73 | 1 | JF706278.1 |
| 74 | 1 | JN381847.1 |
| 75 | 1 | JN381851.1 |
| 76 | 1 | GQ902061.1 |
| 77 | 1 | JN381838.1 |
| 78 | 1 | JN381832.1 |
| 79 | 1 | JF706277.1 |
| 80 | 1 | JN381834.1 |
| 81 | 1 | GU205163.1 |
| 82 | 1 | AF045551.2 |
| 83 | 1 | HM228921.1 |
| 84 | 1 | AB471668.2 |
| 85 | 1 | AB471669.2 |
| 86 | 1 | AB471670.2 |
| 87 | 1 | AB241119.1 |
| 88 | 1 | JF706281.1 |
| 89 | 1 | AB051292.1 |
| 90 | 1 | JN381846.1 |
| 91 | 1 | HQ652538.1 |
| 92 | 1 | FJ495189.1 |
| 93 | 1 | JN381840.1 |
| 94 | 1 | JN381839.1 |
| 95 | 1 | KT957421.1 |
| 96 | 1 | JN381843.1 |
| 97 | 1 | JN381830.1 |
| 98 | 1 | GU187972.1 |
| 99 | 1 | JN381831.1 |
| 100 | 1 | KT957423.1 |
| 101 | 1 | JF706279.1 |
| 102 | 1 | KT957422.1 |
| 103 | 1 | JF706282.1 |
| 104 | 2 | AF217620 |
| 105 | 2 | HQ223287 |
| 106 | 2 | HQ223286 |
| 107 | 2 | HQ223285 |
| 108 | 3 | KU871370.1 |
| 109 | 3 | KU871332.1 |
| 110 | 3 | EF107523.1 |
| 111 | 3 | KU871335.1 |
| 112 | 3 | KU871353.1 |
| 113 | 3 | JN604986.1 |
| 114 | 3 | D90195.1 |
| 115 | 3 | JQ086762.1 |
| 116 | 3 | JX050179.1 |
| 117 | 3 | JX131374.1 |
| 118 | 3 | KT239164.1 |
| 119 | 3 | JN864064.1 |
| 120 | 3 | D90194.1 |
| 121 | 3 | KC517497.1 |
| 122 | 3 | KU871346.1 |
| 123 | 3 | KU871356.1 |
| 124 | 3 | KU871368.1 |
| 125 | 3 | KU871367.1 |
| 126 | 3 | KU871366.1 |
| 127 | 3 | KU871365.1 |
| 128 | 3 | KU871362.1 |
| 129 | 3 | KU871361.1 |
| 130 | 3 | KU871344.1 |
| 131 | 3 | KU871325.1 |
| 132 | 3 | KU871324.1 |
| 133 | 3 | KU871328.1 |
| 134 | 3 | KU871358.1 |
| 135 | 3 | KU871363.1 |
| 136 | 3 | AB196924.1 |
| 137 | 3 | AF486638.1 |
| 138 | 3 | AF014160.1 |
| 139 | 3 | AF014161.1 |
| 140 | 3 | KF907505.1 |
| 141 | 3 | KF667312.1 |
| 142 | 3 | KF667313.1 |
| 143 | 3 | AB196925.1 |
| 144 | 3 | AB196923.1 |
| 145 | 3 | AB196926.1 |
| 146 | 3 | AY303792.1 |
| 147 | 3 | AY303791.1 |
| 148 | 3 | AF254452.1 |
| 149 | 3 | AF254453.1 |
| 150 | 3 | KU871349.1 |
| 151 | 3 | GQ918133.2 |
| 152 | 3 | JF706275.1 |
| 153 | 3 | AB569982.2 |
| 154 | 3 | AB569984.2 |
| 155 | 3 | AB569985.2 |
| 156 | 3 | AB569983.2 |
| 157 | 3 | AB569990.2 |
| 158 | 3 | AB569987.2 |
| 159 | 3 | AB569989.2 |
| 160 | 3 | AB569988.2 |
| 161 | 3 | AB569980.2 |
| 162 | 3 | AB569981.2 |
| 163 | 3 | AY508812.1 |
| 164 | 3 | DD367827.1 |
| 165 | 3 | AY508813.1 |
| 166 | 3 | DD367828.1 |
| 167 | 3 | JF706280.1 |
| 168 | 3 | AB269326.1 |
| 169 | 3 | AF069076.1 |
| 170 | 3 | KU871348.1 |
| 171 | 3 | KU871364.1 |
| 172 | 3 | KU871339.1 |
| 173 | 3 | KU871338.1 |
| 174 | 3 | KU871340.1 |
| 175 | 3 | KX779520.1 |
| 176 | 3 | KX779521.1 |
| 177 | 3 | KU363309.1 |
| 178 | 3 | KC183732.1 |
| 179 | 3 | AY849939.1 |
| 180 | 3 | KR265316.1 |
| 181 | 3 | JN381853.1 |
| 182 | 3 | U47032.1 |
| 183 | 3 | JN381854.1 |
| 184 | 3 | JN381866.1 |
| 185 | 3 | JN381867.1 |
| 186 | 3 | JN381864.1 |
| 187 | 3 | JN381863.1 |
| 188 | 3 | JN381862.1 |
| 189 | 3 | JN381860.1 |
| 190 | 3 | JN381861.1 |
| 191 | 3 | JN381859.1 |
| 192 | 3 | JF706273.1 |
| 193 | 3 | JN381858.1 |
| 194 | 3 | JF706276.1 |
| 195 | 3 | JN381857.1 |
| 196 | 3 | JN381856.1 |
| 197 | 3 | JF706272.1 |
| 198 | 3 | JN381855.1 |
| 199 | 3 | KU871345.1 |
| 200 | 3 | KU871357.1 |
| 201 | 3 | KU871355.1 |
| 202 | 3 | KU871334.1 |
| 203 | 3 | L48961.1 |
| 204 | 3 | L78128.1 |
| 205 | 3 | JN381868.1 |
| 206 | 3 | EF571853.1 |
| 207 | 3 | HE861351.1 |
| 208 | 3 | JN711458.1 |
| 209 | 3 | JN711459.1 |
| 210 | 3 | FJ185036.1 |
| 211 | 3 | FJ185037.1 |
| 212 | 3 | JF706284.1 |
| 213 | 3 | JF706285.1 |
| 214 | 3 | KU871336.1 |
| 215 | 3 | KU821122.1 |
| 216 | 3 | KU871316.1 |
| 217 | 3 | KU871323.1 |
| 218 | 3 | KU871347.1 |
| 219 | 3 | KU871317.1 |
| 220 | 3 | KU871337.1 |
| 221 | 3 | KU871330.1 |
| 222 | 3 | KU871331.1 |
| 223 | 3 | KU871342.1 |
| 224 | 3 | KU871333.1 |
| 225 | 3 | KU871354.1 |
| 226 | 3 | KU871352.1 |
| 227 | 3 | KU871318.1 |
| 228 | 3 | KU871319.1 |
| 229 | 3 | KU871321.1 |
| 230 | 3 | KU871320.1 |
| 231 | 3 | KU871322.1 |
| 232 | 3 | KU871371.1 |
| 233 | 3 | KU871343.1 |
| 234 | 3 | KU871341.1 |
| 235 | 3 | KU323483.1 |
| 236 | 3 | U14163.1 |
| 237 | 3 | M55506.1 |
| 238 | 3 | U15763.1 |
| 239 | 3 | AF416457.1 |
| 240 | 3 | KX779522.1 |
| 241 | 3 | KF297915.1 |
| 242 | 3 | AF315119.1 |
| 243 | 3 | JQ086763.1 |
| 244 | 3 | JN381870.1 |
| 245 | 3 | KU871359.1 |
| 246 | 3 | KU871327.1 |
| 247 | 3 | KU871350.1 |
| 248 | 3 | KU871360.1 |
| 249 | 3 | KU871326.1 |
| 250 | 3 | DI075091.1 |
| 251 | 3 | DI085220.1 |
| 252 | 3 | DD298243.1 |
| 253 | 3 | DD298237.1 |
| 254 | 3 | AY585242.1 |
| 255 | 3 | AY585243.1 |
| 256 | 3 | GQ199609.1 |
| 257 | 3 | AB551991.1 |
| 258 | 3 | AB551992.1 |
| 259 | 3 | KC915016.1 |
| 260 | 3 | KF297916.1 |
| 261 | 3 | JN381873.1 |
| 262 | 3 | JF706269.1 |
| 263 | 3 | JN381872.1 |
| 264 | 3 | JN381869.1 |
| 265 | 3 | EF543861.1 |
| 266 | 3 | AB551990.1 |
| 267 | 3 | KF711994.1 |
| 268 | 3 | M18370.1 |
| 269 | 3 | NC_001437.1 |
| 270 | 3 | KP164498.2 |
| 271 | 3 | AY303795.1 |
| 272 | 3 | AY303798.1 |
| 273 | 3 | AY303796.1 |
| 274 | 3 | AY303797.1 |
| 275 | 3 | AF221499.1 |
| 276 | 3 | AF221500.1 |
| 277 | 3 | AY303793.1 |
| 278 | 3 | AY303794.1 |
| 279 | 3 | KF667310.1 |
| 280 | 3 | KF667311.1 |
| 281 | 3 | KF667314.1 |
| 282 | 3 | KF667315.1 |
| 283 | 3 | JN381871.1 |
| 284 | 3 | EF623987.1 |
| 285 | 3 | JN644310.1 |
| 286 | 3 | GQ902063.1 |
| 287 | 3 | EF623989.1 |
| 288 | 3 | AF075723.1 |
| 289 | 3 | EF623988.1 |
| 290 | 3 | KU871329.1 |
| 291 | 3 | JF706283.1 |
| 292 | 3 | JN381865.1 |
| 293 | 3 | AF098736.1 |
| 294 | 3 | AF098737.1 |
| 295 | 3 | AF098735.1 |
| 296 | 3 | KU871351.1 |
| 297 | 3 | KU871369.1 |
| 298 | 3 | JX072965.1 |
| 299 | 3 | AF080251.1 |
| 300 | 4 | AY184212 |
| 301 | 5 | HM596272 |
| 302 | 5 | JF915894.1 |
| 303 | 5 | KM677246 |

**Table C. Complete list of Primers and Probes Evaluated *In-Silico***

| **Author** | **Target (nt)** | **Oligonucleotides** | **Sequence (5’-3’)*** | **Tm (°C)** |
| --- | --- | --- | --- | --- |
| Pre-existing in-house 2014 (Unpublished) | NS3 (103) | Forward | 6399-TYG-AYG-CRA-GRG-TTT-ATG-CAG | 61-68 |
|  |  | Probe 1 | Fam-AGT-GGT-TTA-AGG-ATT-TTG-CAG-C-Tamra | 62 |
|  |  | Probe 2 | Fam-AGT-GGT-TCA-ARG-ACT-TTG-CAG-C-Tamra | 64-66 |
|  |  | Reverse | 6502-CAT-RCG-ACC-GAG-CAC-CTC-TA | 64-67 |
| Pyke *et al* 2004 | NS5 (62) | Forward | 10230-ATC-TGG-TGY-GGY-AGT-CTC-A | 61-67 |
|  |  | Probe | Fam-CGG-AAC-GCG-ATC-CAG-GGC-AA-Tamra | 69 |
|  |  | Reverse | 10292-CGC-GTA-GAT-GTT-CTC-AGC-CC | 65 |
| Yang *et al* 2004 | 3' UTR (146) | Forward | 10764-GGT-GTA-AGG-ACT-AGA-GGT-TAG-AGG | 64 |
|  |  | Probe | Fam-CCC-GTG-GAA-ACA-ACA-TCA-TGC-GGC-Tamra | 70 |
|  |  | Reverse | 10910-ATT-CCC-AGG-TGT-CAA-TAT-GCT-GTT | 66 |
| Shirato *et al* 2005 | 5' UTR (75) | Forward | 80-AGA-ACG-GAA-GAY-AAC-CAT-GAC-TAA-A | 64-66 |
|  |  | Probe | Fam-ACC-AGG-AGG-GCC-CGG-MGB | 81 |
|  |  | Reverse | 155-CCG-CGT-TTC-AGC-ATA-TTG-AT | 62 |
| Barros *et al*, 2013 | NS2A | Forward | 3548-CCT-TTT-CAG-YTG-GGC-CTT-CTG | 65-67 |
|  |  | Probe | Texas Red-TGA-CCA-TTC-CTG-CGG-TTT-TGG-GG-BHQ | 69 |
|  |  | Reverse | 3682-CAG-TGT-AVG-TVA-TRC-CCC-CAA | 61-68 |
| Huang *et al*, 2004 | NS3 | Forward | 5357- AGA-GCA-CCA-AGG-GAA-TGA-AAT-AGT | 65 |
|  |  | Probe | Fam-CCA-CGC-CAC-TCG-ACC-CAT-AGA-CTG-Tamra | 69 |
|  |  | Reverse | 5452-AAT-AGG-TTG-TAG-TTG-GGC-ACT-CT | 65 |
| **Newly designed as part of this study:** | | | | |
| NS2A (all genotypes) | NS2A (116) | Forward | 3563-AGC-TGG-GCC-TTC-TGG-T | 64 |
|  |  | Probe | Fam-CTT-CGC-AAG-AGG-TGG-ACG-GCC-A-Tamra | 70 |
|  |  | Reverse | 3675-CCC-AAG-CAT-CAG-CAC-AAG | 62 |
| NS2A–Reverse complement | NS2A (112) | Forward | 3563-AGC-TGG-GCC-TTC-TGG-T | 64 |
|  |  | Probe | Fam- TGG-CCG-TCC-ACC-TCT-TGC-GAA-G -Tamra | 70 |
|  |  | Reverse | 3675-CCC-AAG-CAT-CAG-CAC-AAG | 62 |
| NS5 (genotype 1) - v1 | NS5 (315) | Forward | 9925-GDG-CTG-GAT-GGA-ATG-TGA | 61-63 |
|  |  | Probe | Fam-AGG-AGA-GTG-GAT-GAC-CAC-MGB | 75 |
|  |  | Reverse | 10240-CCA-CAC-CAG-ATG-TCC-TC | 60 |
| NS5 (genotype 1) – v2 | NS5 (315) | Forward | 9925-GDG-CTG-GAT-GGA-ATG-TGA | 61-63 |
|  |  | Probe | Fam-AGG-AGA-GTG-GAT-GAC-YAC-MGB | 72-76 |
|  |  | Reverse | 10240-CCA-CAC-CAG-ATG-TCC-TC | 60 |
| NS3 (genotype 3) – v1 | NS3 (141) | Forward | 5726-GCA-ATG-TGC-CTC-CAA-AGA-GC | 65 |
|  |  | Probe | Fam-TCC-TAT-GAY-ACA-GAA-TAY-CCA-AA-MGB | 73-78 |
|  |  | Reverse | 5884-GTC-GAT-GAC-CCT-GCT-CGC | 66 |
| NS3 (genotype 3) – v2 | NS3 (158) | Forward | 5726-GCA-ATG-TGY-CTC-CAA-AGA-GC | 63-66 |
|  |  | Probe | Fam-TCC-TAT-GAY-ACA-GAA-TAY-CCA-AA-MGB | 73-78 |
|  |  | Reverse | 5884-GTC-GAT-GAC-CCT-GCT-CGC | 66 |

*Position of the oligonucleotides is related to the Nakayama strain, Accession number EF571853.

Tms for MGB probes include an additional 15°C added to the Tm calculated IDTNA tool.

NS2Av2 was designed as NS2Av1 with a modification of the probe sequence as its reverse complement.

NS5v2 was designed as NS5v1 with a modification of the probe sequence, with a degeneracy Y inserted instead of C at position 16.

NS3v2 was designed as NS3v1 with a modification of the forward primer sequence, with a degeneracy Y inserted instead of C at position 9.

**Table D. Sequence alignment of Primers and Probes**

**1) Pre-existing In-house Assay (2014)**

Accession 6399 6502

Numbers: ....|....|....|....|....|....|....|....|....|....|....|....|....|..

**TYGAYGCRAGRGTTTATGCAG~~AGTGGTTTAAGGATTTTGCAGC~~TAGAGGTGCTCGGTCGYATG**

**AGTGGTTCAARGACTTTGCAGC**

EF571853.1 .....................~~......................~~....................

AB051292.1 .....................~~......................~~.............c......

AB196923.1 .....................~~................c.....~~....................

AB196924.1 .....................~~................c.....~~....................

AB196925.1 .....................~~................c.....~~....................

AB196926.1 .....................~~................c.....~~....................

AB241118.1 .....................~~......................~~....................

AB241119.1 ................c....~~......................~~.............c......

AB269326.1 .....................~~................c.....~~....................

AB471666.2 .....................~~......................~~.............c......

AB471667.2 .....................~~......................~~.............c......

AB471668.2 .....................~~......................~~.............c......

AB471669.2 .....................~~......................~~.............c......

AB471670.2 .....................~~......................~~.............c......

AB551990.1 .....................~~......................~~....................

AB551991.1 .............a.......~~......................~~....................

AB551992.1 .............a.......~~......................~~....................

AB569980.2 .....................~~................c.....~~....................

AB569981.2 .....................~~................c.....~~....................

AB569982.2 .....................~~................c.....~~.............c......

AB569983.2 .....................~~................c.....~~.............c......

AB569984.2 .....................~~................c.....~~.............c......

AB569985.2 .....................~~................c.....~~.............c......

AB569987.2 .....................~~................c.....~~...........a........

AB569988.2 .....................~~................c.....~~....................

AB569989.2 .....................~~................c.....~~...........a........

AB569990.2 .....................~~................c.....~~....................

AB594829.1 .....................~~......................~~....................

AB698905.1 .....................~~......................~~....................

AB698906.1 .....................~~......................~~....................

AB698907.1 .....................~~......................~~....................

AB698908.1 .....................~~......................~~....................

AB698909.1 .....................~~......................~~....................

AB830335.1 .....................~~......................~~....................

AB853904.1 .....................~~......................~~....................

AB920399.1 .....................~~......................~~....................

AB981183.1 .....................~~......................~~....................

AB981184.1 .....................~~......................~~....................

AF014160.1 .....................~~................c.....~~....................

AF014161.1 .....................~~................c.....~~....................

AF045551.2 .....................~~......................~~....................

AF069076.1 .....................~~................c.....~~....................

AF075723.1 ................c....~~......................~~....................

AF080251.1 .....................~~......................~~....................

AF098735.1 .....................~~................c.....~~......a..c..........

AF098736.1 .....................~~......................~~....................

AF098737.1 ..t..................~~......................~~.......a............

AF217620.1 .....................~~...................g..~~....................

AF221499.1 .......c.............~~......................~~..........t.........

AF221500.1 .......c.............~~......................~~..........t.........

AF254452.1 .....................~~................c.....~~....................

AF254453.1 .....................~~................c.....~~....................

AF315119.1 .....................~~......................~~....................

AF416457.1 .....................~~......................~~....................

AF486638.1 .....................~~................c.....~~....................

AY184212.1 ...................t.~~................c.....~~....a.....t..a......

AY303791.1 .....................~~................c.....~~....................

AY303792.1 .....................~~................c.....~~....................

AY303793.1 .......c.............~~......................~~....a.....t.........

AY303794.1 .......c.............~~......................~~....a.....t.........

AY303795.1 .....................~~......................~~....................

AY303796.1 .....................~~......................~~....................

AY303797.1 .....................~~......................~~....................

AY303798.1 .....................~~......................~~....................

AY316157.1 .....................~~......................~~....................

AY508812.1 .....................~~................c.....~~....................

AY508813.1 .....................~~................c.....~~....................

AY585242.1 .............a..c....~~......................~~....................

AY585243.1 .............a..c....~~......................~~....................

AY849939.1 .....................~~......................~~.......a............

D90194.1 .....................~~......................~~....................

D90195.1 ....................~~......................~~....................

DD298237.1 .............a..c....~~......................~~....................

DD298243.1 .............a..c....~~......................~~....................

DD367827.1 .....................~~................c.....~~....................

DD367828.1 .....................~~................c.....~~....................

DI075091.1 .............a..c....~~......................~~....................

DI085220.1 .............a..c....~~......................~~....................

EF107523.1 .....................~~......................~~.......a............

EF543861.1 .....................~~......................~~....................

EF623987.1 ................c....~~....................t.~~....................

EF623988.1 ........c.......c....~~......................~~....................

EF623989.1 ........c.......c....~~......................~~....................

EU429297.1 .....................~~......................~~....................

EU693899.1 .....................~~......................~~....................

EU880214.1 .....................~~......................~~....................

FJ185036.1 .....................~~......................~~....................

FJ185037.1 .....................~~......................~~....................

FJ495189.1 .....................~~......................~~....................

GQ199609.1 .............a..c....~~......................~~....................

GQ902058.1 ................c....~~......................~~....................

GQ902059.1 ................c....~~......................~~....................

GQ902060.1 ................c....~~......................~~....................

GQ902061.1 .....................~~......................~~....................

GQ902062.1 ................c....~~......................~~.............c......

GQ902063.1 ........c.......c....~~......................~~....................

GQ918133.2 .....................~~................c.....~~....................

GU187972.1 .............c.......~~......................~~....................

GU205163.1 .....................~~......................~~....................

GU556217.1 .....................~~......................~~....................

HE861351.1 .....................~~......................~~....................

HM228921.1 .....................~~......................~~....................

HM366552.1 .....................~~......................~~....................

HM596272.1 .g......c.t..g.....g.~~................c..g..~~..........t..g......

HQ223285.1 ................c..g.~~......................~~....................

HQ223286.1 ...................g.~~......................~~....................

HQ223287.1 ...................g.~~......................~~....................

HQ652538.1 .....................~~......................~~.............c......

HQ893545.1 .....................~~......................~~....................

JF499788.1 .....................~~......................~~....................

JF499789.1 .....................~~......................~~....................

JF499790.1 .....................~~......................~~....................

JF706267.1 .....................~~......................~~....................

JF706268.1 .....................~~......................~~..........t.........

JF706269.1 .............a.......~~......................~~....................

JF706270.1 ...................g.~~......................~~....................

JF706271.1 .....................~~......................~~....................

JF706272.1 ....................~~......................~~....................

JF706273.1 .....................~~......................~~....................

JF706274.1 .....................~~......................~~....................

JF706275.1 .....................~~................c.....~~....................

JF706276.1 .....................~~......................~~....................

JF706277.1 .....................~~......................~~....................

JF706278.1 .....................~~......................~~....................

JF706279.1 .....................~~......................~~....................

JF706280.1 .....................~~................c.....~~....................

JF706281.1 .....................~~......................~~.............c......

JF706282.1 .....................~~......................~~....................

JF706283.1 .....................~~......................~~....................

JF706284.1 .....................~~......................~~....................

JF706285.1 .....................~~......................~~....................

JF706286.1 .....................~~......................~~....................

JF915894.1 .g......c....g..c....~~.a....................~~..........t..g......

JN381830.1 .....................~~......................~~....................

JN381831.1 .............c.......~~......................~~....................

JN381832.1 .....................~~......................~~....................

JN381833.1 .....................~~......................~~..........t.........

JN381834.1 .....................~~......................~~....................

JN381835.1 .....................~~......................~~....................

JN381836.1 .....................~~......................~~....................

JN381837.1 .....................~~......................~~....................

JN381838.1 .....................~~......................~~....................

JN381839.1 .....................~~......................~~....................

JN381840.1 .....................~~......................~~....................

JN381841.1 .....................~~......................~~....................

JN381842.1 .....................~~......................~~....................

JN381843.1 .....................~~......................~~....................

JN381844.1 .....................~~......................~~....................

JN381845.1 .....................~~......................~~....................

JN381846.1 .....................~~......................~~.............c......

JN381847.1 .....................~~......................~~....................

JN381848.1 .....................~~......................~~....................

JN381849.1 .....................~~......................~~....................

JN381850.1 .....................~~......................~~....................

JN381851.1 .....................~~......................~~....................

JN381852.1 .....................~~......................~~....................

JN381853.1 .....................~~......................~~....................

JN381854.1 .....................~~......................~~....................

JN381855.1 .....................~~......................~~....................

JN381856.1 .....................~~......................~~....................

JN381857.1 ....................~~......................~~....................

JN381858.1 .....................~~......................~~....................

JN381859.1 .....................~~......................~~....................

JN381860.1 .....................~~......................~~....................

JN381861.1 .....................~~......................~~....................

JN381862.1 .....................~~......................~~....................

JN381863.1 .....................~~......................~~....................

JN381864.1 .....................~~......................~~....................

JN381865.1 .....................~~......................~~....................

JN381866.1 .....................~~......................~~....................

JN381867.1 .....................~~......................~~....................

JN381868.1 .....................~~......................~~....................

JN381869.1 .....................~~......................~~....................

JN381870.1 .....................~~......................~~....................

JN381871.1 ................c....~~......................~~....................

JN381872.1 .....................~~......................~~....................

JN381873.1 .............a.......~~......................~~....................

JN604986.1 .....................~~......................~~....................

JN711458.1 .....................~~......................~~....................

JN711459.1 .....................~~......................~~....................

JN864064.1 .....................~~......................~~....................

JQ031753.1 .....................~~......................~~....................

JQ086762.1 .....................~~......................~~....................

JQ086763.1 .....................~~............g.........~~....................

JX050179.1 .....................~~......................~~....................

JX072965.1 .....................~~......................~~....................

JX131374.1 .....................~~......................~~....................

KC183732.1 .....................~~......................~~.......a............

KC196115.1 .....................~~......................~~....................

KC517497.1 .....................~~......................~~....................

KC915016.1 .............a.......~~......................~~....................

KF297915.1 .....................~~......................~~....................

KF297916.1 .............a.......~~......................~~....................

KF667310.1 .......c.............~~...................c..~~..........t.........

KF667311.1 .......c.............~~......................~~..........t.........

KF667312.1 .....................~~................c.....~~....................

KF667313.1 .....................~~................c.....~~....................

KF667314.1 .......c.............~~......................~~..........t.........

KF667315.1 .......c.............~~......................~~..........t.........

KF667316.1 .....................~~......................~~....................

KF667317.1 .....................~~......................~~....................

KF667318.1 .....................~~......................~~....................

KF667319.1 .....................~~......................~~....................

KF667320.1 .....................~~......................~~....................

KF667321.1 .....................~~......................~~....................

KF667322.1 .....................~~......................~~....................

KF667323.1 .....................~~......................~~....................

KF667324.1 .....................~~......................~~....................

KF667325.1 .....................~~......................~~....................

KF667326.1 .....................~~......................~~....................

KF667327.1 .....................~~......................~~....................

KF711994.1 .....................~~......................~~....................

KF907505.1 .....................~~................c.....~~....................

KM658163.1 .....................~~......................~~....................

KM677246.1 .g......c.t..g.....g.~~................c..g..~~..........t..g......

KP164498.2 .....................~~......................~~....................

KR265316.1 .....................~~......................~~.......a............

KT229572.1 .....................~~......................~~..........t.........

KT229573.1 .....................~~......................~~....................

KT229574.1 .....................~~......................~~....................

KT229575.1 .....................~~......................~~....................

KT239164.1 .....................~~......................~~....................

KT957419.1 .....................~~......................~~....................

KT957420.1 .....................~~......................~~....................

KT957421.1 .....................~~......................~~....................

KT957422.1 .....................~~......................~~....................

KT957423.1 .....................~~......................~~..a.................

KU323483.1 .....................~~......................~~....................

KU351667.1 .....................~~................c.....~~....................

KU351668.1 .....................~~......................~~....................

KU363309.1 .....................~~......................~~.......a............

KU508408.1 ...................g.~~......................~~....................

KU508409.1 .....................~~......................~~....................

KU821122.1 .....................~~......................~~....................

KU871316.1 .....................~~......................~~....................

KU871317.1 .....................~~......................~~....................

KU871318.1 .....................~~......................~~....................

KU871319.1 .....................~~......................~~....................

KU871320.1 .....................~~......................~~....................

KU871321.1 .....................~~......................~~....................

KU871322.1 .....................~~......................~~....................

KU871323.1 .....................~~......................~~....................

KU871324.1 .....................~~................c.....~~....................

KU871325.1 .....................~~................c.....~~....................

KU871326.1 .....................~~................c.....~~....................

KU871327.1 .....................~~................c.....~~....................

KU871328.1 .....................~~................c.....~~....................

KU871329.1 .....................~~................c.....~~....................

KU871330.1 .....................~~......................~~....................

KU871331.1 .....................~~......................~~....................

KU871332.1 .....................~~................c.....~~....................

KU871333.1 .....................~~......................~~....................

KU871334.1 .....................~~......................~~....................

KU871335.1 .....................~~......................~~....................

KU871336.1 .....................~~......................~~....................

KU871337.1 .....................~~......................~~....................

KU871338.1 .....................~~......................~~....................

KU871339.1 .....................~~......................~~....................

KU871340.1 .....................~~......................~~....................

KU871341.1 .....................~~......................~~....................

KU871342.1 .....................~~......................~~....................

KU871343.1 .....................~~......................~~....................

KU871344.1 .....................~~................c.....~~....................

KU871345.1 .....................~~......................~~....................

KU871346.1 .....................~~................c.....~~....................

KU871347.1 .....................~~......................~~....................

KU871348.1 .....................~~......................~~....................

KU871349.1 .....................~~................c.....~~....................

KU871350.1 .....................~~......................~~....................

KU871351.1 .....................~~......................~~....................

KU871352.1 .....................~~......................~~....................

KU871353.1 .....................~~......................~~....................

KU871354.1 .....................~~......................~~....................

KU871355.1 .....................~~......................~~....................

KU871356.1 .....................~~................c.....~~....................

KU871357.1 .....................~~......................~~....................

KU871358.1 .....................~~................c.....~~....................

KU871359.1 .....................~~................c.....~~....................

KU871360.1 .....................~~................c.....~~....................

KU871361.1 .....................~~................c.....~~....................

KU871362.1 .....................~~................c.....~~....................

KU871363.1 .....................~~................c.....~~....................

KU871364.1 .....................~~......................~~....................

KU871365.1 .....................~~................c.....~~....................

KU871366.1 .....................~~................c.....~~....................

KU871367.1 .....................~~................c.....~~....................

KU871368.1 .....................~~................c.....~~....................

KU871369.1 .....................~~................c.....~~....................

KU871370.1 .....................~~................c.....~~....................

KU871371.1 .....................~~......................~~....................

KX357114.1 .....................~~......................~~....................

KX779520.1 .....................~~......................~~....................

KX779521.1 .....................~~......................~~....................

KX779522.1 .....................~~......................~~....................

L48961.1 .....................~~......................~~....................

L78128.1 .....................~~......................~~.......c.gt.........

M18370.1 .....................~~......................~~....................

M55506.1 .....................~~......................~~....................

NC001437.1 .....................~~......................~~....................

JN644310.1 ................c....~~....................t.~~....................

U14163.1 .....................~~......................~~....................

U15763.1 .....................~~......................~~....................

U47032.1 .....................~~......................~~....................

**2) Pyke assay (2004)**

Accession 10230 10292

Numbers: ....|....|....|....|....|....|....|....|....|....|....|....|..

ATCTGGTGYGGYAGTCTCA~CGGAACGCGATCCAGGGCAA~~GGGCTGAGAACATCTACGCG

EF571853.1 ..............c..t.~...............a....~~................t...

AB051292.1 ...................~..........a.........~~....................

AB196923.1 ..............c....~...............a....~~....................

AB196924.1 ..............c....~...............a....~~....................

AB196925.1 ..............c....~...............a....~~....................

AB196926.1 ..............c....~...............a....~~....................

AB241118.1 ...................~....................~~....................

AB241119.1 ...................~..........a.........~~....................

AB269326.1 ..............c....~...............a....~~....................

AB471666.2 ...................~....................~~...................a

AB471667.2 ...................~....................~~...................a

AB471668.2 ...................~....................~~...................a

AB471669.2 ...................~....................~~...................a

AB471670.2 ...................~....................~~...................a

AB551990.1 ..............c....~...............a....~~................t...

AB551991.1 ..............c....~.........t.....a....~~................t...

AB551992.1 ..............c....~.........t.....a....~~................t...

AB569980.2 ..............c....~...............a....~~..........t.........

AB569981.2 ..............c....~...............a....~~..........t.........

AB569982.2 ..............c....~...............a....~~................t...

AB569983.2 ..............c....~...............a....~~....................

AB569984.2 ..............c....~...............a....~~................t...

AB569985.2 ..............c....~...............a....~~................t...

AB569987.2 ..............c....~...............a....~~....................

AB569988.2 ..............c....~...............a....~~..........t.........

AB569989.2 ..............c....~...............a....~~....................

AB569990.2 ..............c....~......a........a....~~....................

AB594829.1 ...................~....................~~....................

AB698905.1 ...................~....................~~....................

AB698906.1 ...................~......a.............~~....................

AB698907.1 ...................~....................~~....................

AB698908.1 ...................~....................~~....................

AB698909.1 ...................~....................~~....................

AB830335.1 ...................~....................~~...........g........

AB853904.1 ...................~....................~~....................

AB920399.1 ...................~....................~~....................

AB981183.1 ...................~....................~~...........g........

AB981184.1 ...................~....................~~...........g........

AF014160.1 ..............c....~...............a....~~....................

AF014161.1 ..............c....~...............a....~~....................

AF045551.2 ...................~....................~~.............t......

AF069076.1 ..............c....~...............a....~~....................

AF075723.1 ..............c....~...............a....~~................t...

AF080251.1 ..............c....~...............a....~~....................

AF098735.1 ..............c....~...............a....~~....c...............

AF098736.1 ..............c....~...............a....~~....c.....t..t......

AF098737.1 ..............c....~...............a....~~....c.....t..t......

AF217620.1 ...................~....................~~....................

AF221499.1 ..............c....~..........a.........~~................t...

AF221500.1 ..............c....~..........a.........~~................t...

AF254452.1 ..............c....~..............aa....~~....................

AF254453.1 ..............c....~..............aa....~~....................

AF315119.1 ..............c....~...............a....~~................t...

AF416457.1 ..............c....~...............a....~~................t...

AF486638.1 ..............c....~...............a....~~....................

AY184212.1 ..t...........c....~.........cg.t..a....~~................t..a

AY303791.1 ..............c....~...............a....~~....................

AY303792.1 ..............c....~...............a....~~....................

AY303793.1 ..............c....~..........a....a....~~................t...

AY303794.1 ..............c....~..........a....a....~~................t...

AY303795.1 ..............c....~..........a....a....~~................t...

AY303796.1 ..............c....~..........a....a....~~................t...

AY303797.1 ..............c....~..........a....a....~~................t...

AY303798.1 ..............c....~..........a....a....~~................t...

AY316157.1 ...................~....................~~....................

AY508812.1 .............ac....~...............a....~~....................

AY508813.1 ..............c....~...............a....~~....................

AY585242.1 ..............c....~.........t.....a....~~................t...

AY585243.1 ..............c....~.........t.....a....~~................t...

AY849939.1 ..............c....~............t..a....~~................t...

D90194.1 ..............c....~...............a....~~................t...

D90195.1 ..............c....~...............a....~~................t...

DD298237.1 ..............c....~.........t.....a....~~................t...

DD298243.1 ..............c....~.........t.....a....~~................t...

DD367827.1 .............ac....~...............a....~~....................

DD367828.1 ..............c....~...............a....~~....................

DI075091.1 ..............c....~.........t.....a....~~................t...

DI085220.1 ..............c....~.........t.....a....~~................t...

EF107523.1 ..............c....~............t..a....~~................t...

EF543861.1 ..............c....~...............a....~~................t...

EF623987.1 ..............c....~...............a....~~................t...

EF623988.1 ..............c....~...............a....~~................t...

EF623989.1 ..............c....~...............a....~~................t...

EU429297.1 ...................~....................~~....................

EU693899.1 ...................~....................~~....................

EU880214.1 ...................~....................~~...........g........

FJ185036.1 ..............c..t.~...............a....~~................t...

FJ185037.1 ..............c..t.~...............a....~~................t...

FJ495189.1 ...................~....................~~....................

GQ199609.1 ..............c....~.........t.....a....~~................t...

GQ902058.1 ...................~....................~~....................

GQ902059.1 ...................~....................~~....................

GQ902060.1 ...................~....................~~....................

GQ902061.1 ...........a..c....~....................~~....................

GQ902062.1 ...................~....................~~....................

GQ902063.1 ..............c....~t..............a....~~................t...

GQ918133.2 ..............c....~...............a....~~....................

GU187972.1 ...................~....................~~....................

GU205163.1 ...................~....................~~....................

GU556217.1 ...................~....................~~....................

HE861351.1 ..............c..t.~...............a....~~................t...

HM228921.1 ...................~....................~~....................

HM366552.1 ...................~....................~~....................

HM596272.1 ..............ct.g.~..........a.a..a..t.~~....a...........t..a

HQ223285.1 ...................~....................~~....................

HQ223286.1 ...................~..................g.~~....................

HQ223287.1 ...................~.........g..........~~....................

HQ652538.1 ...................~....................~~....................

HQ893545.1 ...................~....................~~....................

JF499788.1 ...................~....................~~....................

JF499789.1 ...................~....................~~....................

JF499790.1 ...................~....................~~....................

JF706267.1 ...................~....................~~....................

JF706268.1 ...................~....................~~....................

JF706269.1 ..............c....~...............a....~~....................

JF706270.1 ..............c....~....................~~...................c

JF706271.1 ...................~....................~~....................

JF706272.1 ..............c....~............t..a....~~................t...

JF706273.1 ..............c....~............t..a....~~................t...

JF706274.1 ...................~....................~~....................

JF706275.1 ..............c....~...............a....~~....................

JF706276.1 ..............c....~............t..a....~~................t...

JF706277.1 ...................~t...................~~....................

JF706278.1 ...................~....................~~....................

JF706279.1 ...................~....................~~....................

JF706280.1 ..............c....~...............a....~~....................

JF706281.1 ...................~....................~~....................

JF706282.1 ...................~....................~~....................

JF706283.1 ..............c....~...............a....~~................t...

JF706284.1 ..............c..t.~...............a....~~................t...

JF706285.1 ..............c..t.~...............a....~~................t...

JF706286.1 ...................~.........g..........~~....................

JF915894.1 ...............t.g.~.........ga.a.....c.~~....a..a........t..a

JN381830.1 ...................~....................~~....................

JN381831.1 ...................~....................~~....................

JN381832.1 ...................~....................~~....................

JN381833.1 ...................~....................~~....................

JN381834.1 ...................~....................~~....................

JN381835.1 ...................~....................~~....................

JN381836.1 ...................~t...................~~....................

JN381837.1 ...................~....................~~.............t......

JN381838.1 ...................~....................~~....................

JN381839.1 ...................~....................~~....................

JN381840.1 ...................~....................~~....................

JN381841.1 ...................~....................~~....................

JN381842.1 ...................~....................~~....................

JN381843.1 ...................~....................~~................t...

JN381844.1 ...................~....................~~....................

JN381845.1 ...................~....................~~....................

JN381846.1 ...................~..........a.........~~....................

JN381847.1 ...................~....................~~....................

JN381848.1 ...................~....................~~....................

JN381849.1 ...................~....................~~....................

JN381850.1 ...................~....................~~....................

JN381851.1 ...................~....................~~....................

JN381852.1 ...................~....................~~....................

JN381853.1 ..............c....~............t..a....~~................t...

JN381854.1 ..............c....~............t..a....~~................t...

JN381855.1 ..............c....~............t..a....~~................t...

JN381856.1 ..............c....~............t..a....~~................t...

JN381857.1 ..............c....~............t..a....~~................t...

JN381858.1 ..............c....~............t..a....~~................t...

JN381859.1 ..............c....~............t..a....~~................t...

JN381860.1 ..............c....~............t..a....~~................t...

JN381861.1 ..............c....~............t..a....~~................t...

JN381862.1 ..............c....~...............a....~~................t...

JN381863.1 ..............c..t.~...............a....~~................t...

JN381864.1 ..............c....~...............a....~~................t...

JN381865.1 ..............c....~...............a....~~................t...

JN381866.1 ..............c....~............t..a....~~................t...

JN381867.1 ..............c....~............t..a....~~................t...

JN381868.1 ..............c..t.~...............a....~~................t...

JN381869.1 ..............c..t.~...............a....~~................t...

JN381870.1 ..............c....~...............a....~~................t...

JN381871.1 ..............c....~...............a....~~................t...

JN381872.1 ..............c....~...............a....~~................t...

JN381873.1 ..............c....~...............a....~~................t...

JN604986.1 ..............c....~...............a....~~................t...

JN711458.1 ..............c..t.~...............a....~~................t...

JN711459.1 ..............c..t.~...............a....~~................t...

JN864064.1 ..............c....~...............a....~~..a.............t...

JQ031753.1 ...................~....................~~....................

JQ086762.1 ..............c....~...............a....~~..a.............t...

JQ086763.1 ..............c....~....t..........a....~~..a.............t...

JX050179.1 ..............c....~...............a....~~....................

JX072965.1 ..............c....~...............a....~~....................

JX131374.1 .............ac....~...............a....~~....................

KC183732.1 ..............c....~............t..a....~~................t...

KC196115.1 ...................~....................~~..............c.....

KC517497.1 ..............c....~...............a....~~................t...

KC915016.1 ..............c....~.........t.....a....~~................t..a

KF297915.1 ..............c....~...............a....~~................t...

KF297916.1 ..............c....~.........t.....a....~~................t..a

KF667310.1 ..............c....~..........a....a....~~................t...

KF667311.1 ..............c....~..........a....a....~~................t...

KF667312.1 ..............c....~...............a....~~....................

KF667313.1 ..............c....~...............a....~~....................

KF667314.1 ..............c....~..........g....a....~~................t...

KF667315.1 ..............c....~..........g....a....~~................t...

KF667316.1 ...................~....................~~....................

KF667317.1 ...................~....................~~....................

KF667318.1 ...................~....................~~................t...

KF667319.1 ...................~....................~~....................

KF667320.1 ...................~....................~~....................

KF667321.1 ...................~....................~~....................

KF667322.1 ...................~....................~~....................

KF667323.1 ...................~....................~~....................

KF667324.1 ...................~....................~~....................

KF667325.1 ...................~....................~~....................

KF667326.1 ...................~....................~~....................

KF667327.1 ...................~....................~~..........t.........

KF711994.1 ..............c....~...............a....~~................t...

KF907505.1 ..............c....~...............a....~~....................

KM658163.1 ...................~....................~~....................

KM677246.1 ..............ct.g.~..........a.a..a..t.~~....a...........t..a

KP164498.2 ..............c....~...............a....~~................t...

KR265316.1 ..............c...g~..a.....a...t..aa...~~...............tt...

KT229572.1 ...................~....................~~....................

KT229573.1 ...................~....................~~....................

KT229574.1 ...................~....................~~.......a............

KT229575.1 ...................~....................~~.......a............

KT239164.1 ..............c....~...............a....~~....................

KT957419.1 ...................~....................~~....................

KT957420.1 ...................~....................~~....................

KT957421.1 ...................~....................~~....................

KT957422.1 ...................~....................~~....................

KT957423.1 ...................~....................~~....................

KU323483.1 ..............c....~...............a....~~................t...

KU351667.1 ..............a....~....................~~....................

KU351668.1 ...................~....................~~....................

KU363309.1 ..............c....~............t..a....~~................t...

KU508408.1 ...................~....................~~....................

KU508409.1 ...................~....................~~....................

KU821122.1 ..............c....~...............a....~~................t...

KU871316.1 ..............c....~...............a....~~................t...

KU871317.1 ..............c....~...............a....~~................t...

KU871318.1 ..............c....~...............a....~~................t...

KU871319.1 ..............c....~...............a....~~................t...

KU871320.1 ..............c....~...............a....~~................t...

KU871321.1 ..............c....~...............a....~~................t...

KU871322.1 ..............c....~...............a....~~................t...

KU871323.1 ..............c....~...............a....~~................t...

KU871324.1 ..............c....~...............a....~~....................

KU871325.1 ..............c....~...............a....~~....................

KU871326.1 ..............c....~...............a....~~....................

KU871327.1 ..............c....~...............a....~~....................

KU871328.1 ..............c....~...............a....~~....................

KU871329.1 ..............c....~...............a....~~....................

KU871330.1 ..............c....~...............a....~~................t...

KU871331.1 ..............c....~...............a....~~................t...

KU871332.1 ..............c....~...............a....~~....................

KU871333.1 ..............c....~...............a....~~................t...

KU871334.1 ..............c....~...............a....~~................t...

KU871335.1 ..............c....~...............a....~~................t...

KU871336.1 ..............c....~...............a....~~................t...

KU871337.1 ..............c....~...............a....~~................t...

KU871338.1 ..............c....~...............a....~~................t...

KU871339.1 ..............c....~...............a....~~................t...

KU871340.1 ..............c....~...............a....~~................t...

KU871341.1 ..............c....~...............a....~~................t...

KU871342.1 ..............c....~...............a....~~................t...

KU871343.1 ..............c....~...............a....~~................t...

KU871344.1 ..............c....~...............a....~~....................

KU871345.1 ..............c....~...............a....~~................t...

KU871346.1 ..............c....~...............a....~~....................

KU871347.1 ..............c....~...............a....~~................t...

KU871348.1 ..............c....~...............a....~~................t...

KU871349.1 ..............c....~...............a....~~....................

KU871350.1 ..............c....~...............a....~~....................

KU871351.1 ..............c....~...............a....~~................t...

KU871352.1 ..............c....~...............a....~~................t...

KU871353.1 ..............c....~...............a....~~................t...

KU871354.1 ..............c....~...............a....~~................t...

KU871355.1 ..............c....~...............a....~~................t...

KU871356.1 ..............c....~...............a....~~....................

KU871357.1 ..............c....~...............a....~~................t...

KU871358.1 ..............c....~...............a....~~....................

KU871359.1 ..............c....~...............a....~~....................

KU871360.1 ..............c....~...............a....~~....................

KU871361.1 ..............c....~...............a....~~....................

KU871362.1 ..............c....~...............a....~~....................

KU871363.1 ..............c....~...............a....~~....................

KU871364.1 ..............c....~...............a....~~................t...

KU871365.1 ..............c....~...............a....~~....................

KU871366.1 ..............c....~...............a....~~....................

KU871367.1 ..............c....~...............a....~~....................

KU871368.1 ..............c....~...............a....~~....................

KU871369.1 ..............c....~...............a....~~....................

KU871370.1 ..............c....~...............a....~~....................

KU871371.1 ..............c....~...............a....~~................t...

KX357114.1 ...................~....................~~..............c.....

KX779520.1 ..............c....~...............a....~~................t...

KX779521.1 ..............c....~...............a....~~................t...

KX779522.1 ..............c....~...............a....~~................t...

L48961.1 ..............c..t.~...............a....~~................t...

L78128.1 ..............c..t.~...............a....~~................t...

M18370.1 ..............c....~...............a....~~................t...

M55506.1 ..............c....~...............a....~~................t...

NC_001437.1 ..............c....~...............a....~~................t...

JN644310.1 ..............c....~...............a....~~................t...

U14163.1 ..............c....~...............a....~~................t...

U15763.1 ..............c....~...............a....~~................t...

U47032.1 ..............c....~............t..a....~~................t...

**3) Yang assay (2004)**

Accession Numbers: 10764 10910

....|....|....|....|....|....|....|....|....|....|....|....|....|....|....|.

GGTGTAAGGACTAGAGGTTAGAGG~~CCCGTGGAAACAACATCATGCGG~~CAACAGCATATTGACACCTGGGAAT

EF571853.1 ........................~~...............a.......~~.........................

AB051292.1 ........................~~................t......~~.........................

AB196923.1 ........................~~...............a.......~~.........................

AB196924.1 ........................~~...............a.......~~.........................

AB196925.1 ........................~~...............a.......~~.........................

AB196926.1 ........................~~...............a.......~~.........................

AB241118.1 ........................~~................t......~~.........................

AB241119.1 ........................~~................tg.....~~.........................

AB269326.1 ------------------------~~-----------------------~~-------------------------

AB471666.2 ........................~~.......................~~.........................

AB471667.2 ........................~~.......................~~.........................

AB471668.2 ........................~~.......................~~.........................

AB471669.2 ........................~~.......................~~.........................

AB471670.2 ........................~~.......................~~.........................

AB551990.1 ........................~~...............a.......~~.........................

AB551991.1 ........................~~............g..a.......~~.........................

AB551992.1 ........................~~............g..a.......~~.........................

AB569980.2 ........................~~...............a.......~~.........................

AB569981.2 ........................~~...............a.......~~.........................

AB569982.2 ........................~~...............a.......~~.......................g.

AB569983.2 ........................~~...............a.......~~.......................g.

AB569984.2 ........................~~...............a.......~~.......................g.

AB569985.2 ........................~~...............a.......~~.......................g.

AB569987.2 ........................~~...............a.......~~.........................

AB569988.2 ........................~~...............a.......~~.........................

AB569989.2 ........................~~...............a.......~~.........................

AB569990.2 ........................~~...............a.......~~.........................

AB594829.1 ........................~~................t......~~.........................

AB698905.1 ........................~~................t......~~.........................

AB698906.1 ........................~~................t......~~.........................

AB698907.1 ........................~~................t......~~.........................

AB698908.1 ........................~~................t......~~.........................

AB698909.1 ........................~~................t......~~.........................

AB830335.1 ........................~~................tg.....~~.........................

AB853904.1 ........................~~................t......~~.........................

AB920399.1 ........................~~................t......~~.........................

AB981183.1 ........................~~................tg.....~~.........................

AB981184.1 ........................~~................tg.....~~.........................

AF014160.1 ........................~~...............a.......~~.........................

AF014161.1 ........................~~...............a.......~~.........................

AF045551.2 ........................~~.............a..t......~~.........................

AF069076.1 ........................~~...............a.......~~.........................

AF075723.1 ........................~~..........t....a.......~~.........................

AF080251.1 ........................~~...............a.......~~.........................

AF098735.1 ........................~~...............a.......~~.........................

AF098736.1 ........................~~...............a.......~~.........................

AF098737.1 ........................~~...............a.......~~.....................ag..

AF217620.1 ........................~~...............at......~~.........................

AF221499.1 ........................~~.............t.a.......~~.........................

AF221500.1 ........................~~.............t.a.......~~.........................

AF254452.1 ........................~~...............a.......~~.........................

AF254453.1 ........................~~...............a.......~~.........................

AF315119.1 ........................~~...............a.......~~.........................

AF416457.1 ........................~~...............a.......~~.........................

AF486638.1 ........................~~...............a.......~~.........................

AY184212.1 .a......................~~...............a.......~~.........................

AY303791.1 ........................~~...............a.......~~.........................

AY303792.1 ........................~~...............a.......~~.........................

AY303793.1 ........................~~.............tga.......~~.........................

AY303794.1 ........................~~.............tga.......~~.........................

AY303795.1 ........................~~.............t.a.......~~.........................

AY303796.1 ........................~~.............t.a.......~~.........................

AY303797.1 ........................~~.............t.a.......~~.........................

AY303798.1 ........................~~.............t.a.......~~.........................

AY316157.1 ........................~~.......................~~.........................

AY508812.1 ........................~~...............a.......~~.........................

AY508813.1 ........................~~...............a.......~~.........................

AY585242.1 ........................~~...............a.......~~.........................

AY585243.1 ........................~~...............a.......~~.........................

AY849939.1 ........................~~...............a.......~~.........................

D90194.1 ........................~~...............a.......~~.........................

D90195.1 ........................~~...............at......~~.........................

DD298237.1 ........................~~...............a.......~~.................--------

DD298243.1 ........................~~...............a.......~~.........................

DD367827.1 ........................~~...............a.......~~.........................

DD367828.1 ........................~~...............a.......~~.........................

DI075091.1 ........................~~...............a.......~~.................--------

DI085220.1 ........................~~...............a.......~~.........................

EF107523.1 ........................~~...............a.......~~.........................

EF543861.1 ........................~~...............a.......~~.........................

EF623987.1 ........................~~..........t....a.......~~.........................

EF623988.1 ........................~~..........t....a.......~~.........................

EF623989.1 ........................~~..........t....a.......~~.........................

EU429297.1 ........................~~................t......~~.........................

EU693899.1 ........................~~................t......~~.........................

EU880214.1 ........................~~................t......~~..g......................

FJ185036.1 ........................~~...............a.......~~.........................

FJ185037.1 ........................~~...............a.......~~.........................

FJ495189.1 ........................~~................t......~~.........................

GQ199609.1 ........................~~...............a.......~~.........................

GQ902058.1 ........................~~...............a.......~~.........................

GQ902059.1 ........................~~...............a.......~~.........................

GQ902060.1 ........................~~...............at......~~.........................

GQ902061.1 ........................~~...............a.......~~.........................

GQ902062.1 ........................~~...............a.......~~.........................

GQ902063.1 ........................~~...............a.......~~.........................

GQ918133.2 ........................~~...............a.......~~.........................

GU187972.1 ........................~~................t......~~.........................

GU205163.1 ........................~~.......................~~.........................

GU556217.1 ........................~~................t......~~.........................

HE861351.1 ........................~~...............a.......~~.........................

HM228921.1 ........................~~.............t..t......~~.........................

HM366552.1 ........................~~................t......~~.........................

HM596272.1 ..c.....................~~.........--..g.ag......~~.......................t.

HQ223285.1 ------------------------~~-----------------------~~-------------------------

HQ223286.1 ------------------------~~-----------------------~~-------------------------

HQ223287.1 ------------------------~~-----------------------~~-------------------------

HQ652538.1 ........................~~................tg.....~~.........................

HQ893545.1 ........................~~................t......~~.........................

JF499788.1 ........................~~................ac.....~~.........................

JF499789.1 ........................~~................t......~~.........................

JF499790.1 ........................~~.......................~~.........................

JF706267.1 ........................~~................tg.....~~.........................

JF706268.1 ........................~~................t......~~.........................

JF706269.1 ........................~~...............a.......~~.........................

JF706270.1 ........................~~................t.....c~~.........................

JF706271.1 ........................~~....c...........t......~~.........................

JF706272.1 ........................~~............g..a.......~~.........................

JF706273.1 ........................~~...............a.......~~.........................

JF706274.1 ........................~~................t......~~.........................

JF706275.1 ........................~~...............a.......~~.........................

JF706276.1 ........................~~............g..a.......~~.........................

JF706277.1 ........................~~.......................~~.........................

JF706278.1 ........................~~.......................~~.........................

JF706279.1 ........................~~...............at......~~.........................

JF706280.1 ........................~~...............a.......~~.........................

JF706281.1 ........................~~................t......~~.........................

JF706282.1 ........................~~...............at......~~.........................

JF706283.1 ........................~~...............a.......~~.........................

JF706284.1 ........................~~...............a.......~~.........................

JF706285.1 ........................~~...............a.......~~.........................

JF706286.1 ........................~~................t......~~.........................

JF915894.1 ..c.....................~~.........--..g.aa......~~.......................t.

JN381830.1 ........................~~................t......~~.........................

JN381831.1 ........................~~................t......~~.........................

JN381832.1 ........................~~.......................~~.........................

JN381833.1 ........................~~................t......~~.........................

JN381834.1 ........................~~................t......~~.........................

JN381835.1 ........................~~................t......~~.........................

JN381836.1 ........................~~................t......~~.........................

JN381837.1 ........................~~................t......~~.........................

JN381838.1 ........................~~................t......~~.........................

JN381839.1 ......t.................~~.........tt.....t.....t~~.........................

JN381840.1 ........................~~................t......~~.........................

JN381841.1 ........................~~................t......~~.........................

JN381842.1 ........................~~................t......~~.........................

JN381843.1 ........................~~.......................~~.........................

JN381844.1 ........................~~................t......~~.........................

JN381845.1 ........................~~................t......~~.........................

JN381846.1 ........................~~................t......~~.........................

JN381847.1 ........................~~................t......~~.........................

JN381848.1 ........................~~.......................~~.........................

JN381849.1 ........................~~................t......~~.........................

JN381850.1 ........................~~................t......~~.........................

JN381851.1 ........................~~................t......~~.........................

JN381852.1 ........................~~................t......~~.........................

JN381853.1 ........................~~...............a.......~~.........................

JN381854.1 ........................~~...............a.......~~.........................

JN381855.1 ........................~~............g..a.......~~.........................

JN381856.1 ........................~~............g..a.......~~.........................

JN381857.1 ........................~~............g..a.......~~.........................

JN381858.1 ........................~~............g..a.......~~.........................

JN381859.1 ........................~~............g..a.......~~.........................

JN381860.1 ........................~~...............a.......~~.........................

JN381861.1 ........................~~............g..a.......~~.........................

JN381862.1 ........................~~...............a.......~~.........................

JN381863.1 ........................~~...............a.......~~.........................

JN381864.1 ........................~~...............a.......~~.........................

JN381865.1 ........................~~.............t.a.......~~.........................

JN381866.1 ........................~~............g..a.......~~.........................

JN381867.1 ........................~~...............a.......~~.........................

JN381868.1 ........................~~...............a.......~~.........................

JN381869.1 ........................~~...............a.......~~.........................

JN381870.1 ........................~~...............a.......~~.........................

JN381871.1 ........................~~...............a.......~~.........................

JN381872.1 ........................~~............g..a.......~~.........................

JN381873.1 ........................~~...............a.......~~.........................

JN604986.1 ........................~~...............a.......~~.........................

JN711458.1 ........................~~...............a.......~~.........................

JN711459.1 ...............-........~~...............a.......~~.........................

JN864064.1 ........................~~...............at......~~.........................

JQ031753.1 ........................~~.......................~~.........................

JQ086762.1 ........................~~...............at......~~.........................

JQ086763.1 ........................~~...............at......~~.........................

JX050179.1 ........................~~...............a.......~~.........................

JX072965.1 ........................~~...............a.......~~.........................

JX131374.1 ........................~~...............a.......~~.........................

KC183732.1 ------------------------~~-----------------------~~-------------------------

KC196115.1 ........................~~.......................~~.........................

KC517497.1 ........................~~...............a.......~~.........................

KC915016.1 ........................~~...............a.......~~.........................

KF297915.1 ........................~~...............a.......~~.........................

KF297916.1 ........................~~...............a.......~~.........................

KF667310.1 ........................~~.............tga.......~~.........................

KF667311.1 ........................~~.............t.a.......~~.........................

KF667312.1 ........................~~...............a.......~~.........................

KF667313.1 ........................~~...............a.......~~.........................

KF667314.1 ........................~~...............a.......~~.........................

KF667315.1 ........................~~...............a.......~~.........................

KF667316.1 ........................~~................t......~~.........................

KF667317.1 ........................~~................t......~~.........................

KF667318.1 ........................~~.......................~~.........................

KF667319.1 ........................~~.......................~~.........................

KF667320.1 ........................~~.......................~~.........................

KF667321.1 ........................~~...........g....t......~~.........................

KF667322.1 ........................~~........t.......t......~~.........................

KF667323.1 ........................~~.......................~~.........................

KF667324.1 ........................~~.......................~~.........................

KF667325.1 ........................~~................t......~~.........................

KF667326.1 ........................~~.......................~~.........................

KF667327.1 ........................~~.......................~~.........................

KF711994.1 ------------------------~~-----------------------~~-------------------------

KF907505.1 ........................~~...............a.......~~.........................

KM658163.1 ........................~~................t......~~.........................

KM677246.1 ..c.....................~~.........--..g.ag......~~.......................t.

KP164498.2 ........................~~...............at......~~.........................

KR265316.1 ........................~~...............a.......~~.........................

KT229572.1 ........................~~................ag.....~~.........................

KT229573.1 ........................~~................tg.....~~.........................

KT229574.1 ........................~~................t......~~.........................

KT229575.1 ........................~~................t......~~.........................

KT239164.1 ........................~~...............a.......~~.........................

KT957419.1 ........................~~................tg.....~~.........................

KT957420.1 ........................~~................t......~~.........................

KT957421.1 ........................~~...........g....t......~~.........................

KT957422.1 ........................~~...............at......~~.........................

KT957423.1 ........................~~...............at......~~.........................

KU323483.1 ........................~~...............a.......~~.........................

KU351667.1 ........................~~................t......~~.........................

KU351668.1 ........................~~................t......~~.........................

KU363309.1 ........................~~...............a.......~~.........................

KU508408.1 ........................~~................t......~~.........................

KU508409.1 ........................~~................t......~~.........................

KU821122.1 ------------------------~~-----------------------~~-------------------------

KU871316.1 ------------------------~~-----------------------~~-------------------------

KU871317.1 ------------------------~~-----------------------~~-------------------------

KU871318.1 ------------------------~~-----------------------~~-------------------------

KU871319.1 ------------------------~~-----------------------~~-------------------------

KU871320.1 ------------------------~~-----------------------~~-------------------------

KU871321.1 ------------------------~~-----------------------~~-------------------------

KU871322.1 ------------------------~~-----------------------~~-------------------------

KU871323.1 ------------------------~~-----------------------~~-------------------------

KU871324.1 ------------------------~~-----------------------~~-------------------------

KU871325.1 ------------------------~~-----------------------~~-------------------------

KU871326.1 ------------------------~~-----------------------~~-------------------------

KU871327.1 ------------------------~~-----------------------~~-------------------------

KU871328.1 ------------------------~~-----------------------~~-------------------------

KU871329.1 ------------------------~~-----------------------~~-------------------------

KU871330.1 ------------------------~~-----------------------~~-------------------------

KU871331.1 ------------------------~~-----------------------~~-------------------------

KU871332.1 ------------------------~~-----------------------~~-------------------------

KU871333.1 ------------------------~~-----------------------~~-------------------------

KU871334.1 ------------------------~~-----------------------~~-------------------------

KU871335.1 ------------------------~~-----------------------~~-------------------------

KU871336.1 ------------------------~~-----------------------~~-------------------------

KU871337.1 ------------------------~~-----------------------~~-------------------------

KU871338.1 ------------------------~~-----------------------~~-------------------------

KU871339.1 ------------------------~~-----------------------~~-------------------------

KU871340.1 ------------------------~~-----------------------~~-------------------------

KU871341.1 ------------------------~~-----------------------~~-------------------------

KU871342.1 ------------------------~~-----------------------~~-------------------------

KU871343.1 ------------------------~~-----------------------~~-------------------------

KU871344.1 ------------------------~~-----------------------~~-------------------------

KU871345.1 ------------------------~~-----------------------~~-------------------------

KU871346.1 ------------------------~~-----------------------~~-------------------------

KU871347.1 ------------------------~~-----------------------~~-------------------------

KU871348.1 ------------------------~~-----------------------~~-------------------------

KU871349.1 ------------------------~~-----------------------~~-------------------------

KU871350.1 ------------------------~~-----------------------~~-------------------------

KU871351.1 ------------------------~~-----------------------~~-------------------------

KU871352.1 ------------------------~~-----------------------~~-------------------------

KU871353.1 ------------------------~~-----------------------~~-------------------------

KU871354.1 ------------------------~~-----------------------~~-------------------------

KU871355.1 ------------------------~~-----------------------~~-------------------------

KU871356.1 ------------------------~~-----------------------~~-------------------------

KU871357.1 ------------------------~~-----------------------~~-------------------------

KU871358.1 ------------------------~~-----------------------~~-------------------------

KU871359.1 ------------------------~~-----------------------~~-------------------------

KU871360.1 ------------------------~~-----------------------~~-------------------------

KU871361.1 ------------------------~~-----------------------~~-------------------------

KU871362.1 ------------------------~~-----------------------~~-------------------------

KU871363.1 ------------------------~~-----------------------~~-------------------------

KU871364.1 ------------------------~~-----------------------~~-------------------------

KU871365.1 ------------------------~~-----------------------~~-------------------------

KU871366.1 ------------------------~~-----------------------~~-------------------------

KU871367.1 ------------------------~~-----------------------~~-------------------------

KU871368.1 ------------------------~~-----------------------~~-------------------------

KU871369.1 ------------------------~~-----------------------~~-------------------------

KU871370.1 ------------------------~~-----------------------~~-------------------------

KU871371.1 ------------------------~~-----------------------~~-------------------------

KX357114.1 ........................~~...............at......~~.........................

KX779520.1 ........................~~.....-.........--------~~-------------------------

KX779521.1 ........................~~...............a-------~~-------------------------

KX779522.1 ........................~~...............a-------~~-------------------------

L48961.1 ........................~~...............a.......~~.........................

L78128.1 ........................~~...............a.......~~.........................

M18370.1 ........................~~...............at......~~.........................

M55506.1 ........................~~...............a.......~~.........................

NC_001437.1 ........................~~...............at......~~.........................

JN644310.1 ........................~~...--------------------~~-------------------------

U14163.1 ........................~~...............a.......~~.........................

U15763.1 ........................~~...............a.......~~.........................

U47032.1 ........................~~...............a.......~~.........................

**4) Shirato assay (2005)**

Accession Numbers: 80 155

....|....|....|....|....|....|....|....|....|....|....|....|....

**AGAACGGAAGAYAACCATGACTAAA~~ACCAGGAGGGCCCGG~~ATCAATATGCTGAAACGCGG**

**EF571853.1**  **..........-at.a.catgact..~~...............~~....................**

**AB051292.1**  **...........ac.a.catgact..~~...............~~....................**

**AB196923.1**  **..........-at.a.catgact..~~...............~~....................**

**AB196924.1**  **..........-at.a.catgact..~~...............~~....................**

**AB196925.1**  **..........-at.a.catgact..~~...............~~....................**

**AB196926.1**  **..........-at.a.catgact..~~...............~~....................**

**AB241118.1**  **...........ac.a.catgact..~~...............~~....................**

**AB241119.1**  **...........ac.a.catgact..~~...............~~....................**

**AB269326.1**  **..........-at.a.catgact..~~...............~~....................**

**AB471666.2**  **...........ac.a.catgact..~~...............~~....................**

**AB471667.2**  **...........ac.a.catgact..~~...............~~....................**

**AB471668.2**  **...........ac.a.catgact..~~...............~~....................**

**AB471669.2**  **...........ac.a.catgact..~~...............~~....................**

**AB471670.2**  **...........ac.a.catgact..~~...............~~....................**

**AB551990.1**  **..........-at.a.catgact..~~...............~~....................**

**AB551991.1**  **..........-at.a.catgact..~~...............~~....................**

**AB551992.1**  **..........-at.a.catgact..~~...............~~....................**

**AB569980.2**  **..........-at.a.catgact..~~...............~~....................**

**AB569981.2**  **..........-at.a.catgact..~~...............~~....................**

**AB569982.2**  **..........-at.a.catgact..~~...............~~....................**

**AB569983.2**  **..........-at.a.catgact..~~...............~~....................**

**AB569984.2**  **..........-at.a.catgact..~~...............~~....................**

**AB569985.2**  **..........-at.a.catgact..~~...............~~....................**

**AB569987.2**  **..........-at.a.catgact..~~...............~~....................**

**AB569988.2**  **..........-at.a.catgact..~~...............~~....................**

**AB569989.2**  **..........-at.a.catgact..~~...............~~....................**

**AB569990.2**  **..........-at.a.catgact..~~...............~~....................**

**AB594829.1**  **...........ac.a.catgact..~~...............~~....................**

**AB698905.1**  **...........ac.a.catgact..~~...............~~....................**

**AB698906.1**  **...........ac.a.catgact..~~...............~~....................**

**AB698907.1**  **...........ac.a.catgact..~~...............~~....................**

**AB698908.1**  **...........ac.a.catgact..~~...............~~....................**

**AB698909.1**  **...........ac.a.catgact..~~...............~~....................**

**AB830335.1**  **...........ac.a.catgact..~~...............~~....................**

**AB853904.1**  **...........ac.a.catgact..~~...............~~....................**

**AB920399.1**  **...........ac.a.catgact..~~...............~~....................**

**AB981183.1**  **...........ac.a.catgact..~~...............~~....................**

**AB981184.1**  **...........ac.a.catgact..~~...............~~....................**

**AF014160.1**  **..........-at.a.catgact..~~...............~~....................**

**AF014161.1**  **..........-at.a.catgact..~~...............~~....................**

**AF045551.2**  **..........-ac.a.catgact..~~...............~~....................**

**AF069076.1**  **..........-at.a.catgact..~~...............~~....................**

**AF075723.1**  **..........-at.a.catgact..~~...............~~....................**

**AF080251.1**  **..........-at.a.catgact..~~...............~~....................**

**AF098735.1**  **..........-at.a.catgact..~~...............~~....................**

**AF098736.1**  **..........-at.a.catgact..~~...............~~....................**

**AF098737.1**  **..........-at.a.catgact..~~...............~~....................**

**AF217620.1**  **..........-ac.a.catgact..~~...............~~....................**

**AF221499.1**  **..........-at.a.catgact..~~...............~~....................**

**AF221500.1**  **..........-at.a.catgact..~~...............~~....................**

**AF254452.1**  **..........-at.a.catgact..~~...............~~....................**

**AF254453.1**  **..........-at.a.catgact..~~...............~~....................**

**AF315119.1**  **..........-at.a.catgact..~~...............~~....................**

**AF416457.1**  **..........-at.a.catgact..~~...............~~....................**

**AF486638.1**  **..........-at.a.catgact..~~...............~~....................**

**AY184212.1**  **.a........-at.t.catgact..~~....c..........~~...t................**

**AY303791.1**  **..........-at.a.catgact..~~...............~~....................**

**AY303792.1**  **..........-at.a.catgact..~~...............~~....................**

**AY303793.1**  **..........-at.a.catgact..~~...............~~....................**

**AY303794.1**  **..........-at.a.catgact..~~...............~~....................**

**AY303795.1**  **..........-at.a.catgact..~~...............~~....................**

**AY303796.1**  **..........-at.a.catgact..~~...............~~....................**

**AY303797.1**  **..........-at.a.catgact..~~...............~~....................**

**AY303798.1**  **..........-at.a.catgact..~~...............~~....................**

**AY316157.1**  **...........a-.a.catgact..~~...............~~....................**

**AY508812.1**  **..........-at.a.catgact..~~...............~~....................**

**AY508813.1**  **..........-at.a.catgact..~~...............~~....................**

**AY585242.1**  **..........-at.a.catgact..~~...............~~....................**

**AY585243.1**  **..........-at.a.catgact..~~...............~~....................**

**AY849939.1**  **..........-at.a.catgact..~~...............~~....................**

**D90194.1**  **..........-at.a.catgact..~~...............~~....................**

**D90195.1**  **..........-at.a.catgact..~~...............~~....................**

**DD298237.1**  **..........-at.a.catgact..~~...............~~....................**

**DD298243.1**  **..........-at.a.catgact..~~...............~~....................**

**DD367827.1**  **..........-at.a.catgact..~~...............~~....................**

**DD367828.1**  **..........-at.a.catgact..~~...............~~....................**

**DI075091.1**  **..........-at.a.catgact..~~...............~~....................**

**DI085220.1**  **..........-at.a.catgact..~~...............~~....................**

**EF107523.1**  **..........-at.a.catgact..~~...............~~....................**

**EF543861.1**  **..........-at.a.catgact..~~...............~~....................**

**EF623987.1**  **..........-at.a.catgact..~~...............~~....................**

**EF623988.1**  **..........-at.a.catgact..~~...............~~....................**

**EF623989.1**  **..........-at.a.catgact..~~...............~~....................**

**EU429297.1**  **...........ac.a.catgact..~~...............~~....................**

**EU693899.1**  **...........ac.a.catgact..~~...............~~....................**

**EU880214.1**  **...........ac.a.catgact..~~...............~~....................**

**FJ185036.1**  **..........-at.a.catgact..~~...............~~....................**

**FJ185037.1**  **..........-at.a.catgact..~~...............~~....................**

**FJ495189.1**  **...........ac.a.catgact..~~...............~~....................**

**GQ199609.1**  **..........-at.a.catgact..~~...............~~....................**

**GQ902058.1**  **...........ac.a.catgact..~~...............~~....................**

**GQ902059.1**  **...........ac.a.catgact..~~...............~~....................**

**GQ902060.1**  **...........ac.a.catgact..~~...............~~....................**

**GQ902061.1**  **...........ac.a.catgact..~~...............~~....................**

**GQ902062.1**  **...........ac.a.catgact..~~...............~~....................**

**GQ902063.1**  **...........at.a.catgact..~~...............~~....................**

**GQ918133.2**  **..........-at.a.catgact..~~...............~~....................**

**GU187972.1**  **...........ac.a.catgact..~~...............~~....................**

**GU205163.1**  **...........ac.a.catgact..~~...............~~....................**

**GU556217.1**  **...g.......ac.a.catgact..~~...............~~....................**

**HE861351.1**  **..........-at.a.catgact..~~...............~~....................**

**HM228921.1**  **...........ac.a.catgact..~~...............~~....................**

**HM366552.1**  **...........ac.a.catgact..~~...............~~....................**

**HM596272.1**  **..........-a..a.catgact..~~...............~~....................**

**HQ223285.1**  **-----------------atgact..~~...............~~....................**

**HQ223286.1**  **-----------------atgact..~~...............~~....................**

**HQ223287.1**  **-----------------atgact..~~...............~~....................**

**HQ652538.1**  **...........ac.a.catgact..~~...............~~....................**

**HQ893545.1**  **...........ac.a.catgact..~~...............~~....................**

**JF499788.1**  **...........ac.a.catgact..~~...............~~....................**

**JF499789.1**  **...........ac.a.catgact..~~...............~~....................**

**JF499790.1**  **...........ac.a.catgact..~~...............~~....................**

**JF706267.1**  **...........ac.a.catgact..~~...............~~....................**

**JF706268.1**  **...........ac.a.catgact..~~...............~~....................**

**JF706269.1**  **..........-at.a.catgact..~~...............~~....................**

**JF706270.1**  **...........ac.a.catgact..~~...............~~....................**

**JF706271.1**  **...........ac.a.catgact..~~...............~~....................**

**JF706272.1**  **..........-at.a.catgact..~~...............~~....................**

**JF706273.1**  **..........-at.a.catgact..~~...............~~....................**

**JF706274.1**  **...........ac.a.catgact..~~...............~~....................**

**JF706275.1**  **..........-at.a.catgact..~~...............~~....................**

**JF706276.1**  **..........-at.a.catgact..~~...............~~....................**

**JF706277.1**  **...........ac.a.catgact..~~...............~~....................**

**JF706278.1**  **...........ac.a.catgact..~~...............~~....................**

**JF706279.1**  **...........ac.a.catgact..~~...............~~....................**

**JF706280.1**  **..........-at.a.catgact..~~...............~~....................**

**JF706281.1**  **...........ac.a.catgact..~~...............~~....................**

**JF706282.1**  **...........ac.a.catgact..~~...............~~....................**

**JF706283.1**  **..........-at.a.catgact..~~...............~~....................**

**JF706284.1**  **..........-at.a.catgact..~~...............~~....................**

**JF706285.1**  **..........-at.a.catgact..~~...............~~....................**

**JF706286.1**  **...........ac.a.catgact..~~...............~~....................**

**JF915894.1**  **..........-a..a.catgact..~~...............~~....................**

**JN381830.1**  **...........ac.a.catgact..~~...............~~....................**

**JN381831.1**  **...........ac.a.catgact..~~...............~~....................**

**JN381832.1**  **...........ac.a.catgact..~~...............~~....................**

**JN381833.1**  **...........ac.a.catgact..~~...............~~....................**

**JN381834.1**  **...........ac.a.catgact..~~...............~~....................**

**JN381835.1**  **...........ac.a.catgact..~~...............~~....................**

**JN381836.1**  **...........ac.a.catgact..~~...............~~....................**

**JN381837.1**  **...........ac.a.catgact..~~...............~~....................**

**JN381838.1**  **...........ac.a.catgact..~~...............~~....................**

**JN381839.1**  **...........ac.a.catgact..~~...............~~....................**

**JN381840.1**  **...........ac.a.catgact..~~...............~~....................**

**JN381841.1**  **...........ac.a.catga.t..~~...............~~....................**

**JN381842.1**  **...........ac.a.catgact..~~...............~~....................**

**JN381843.1**  **...........ac.a.catga.t..~~...............~~.........t.......g..**

**JN381844.1**  **...........ac.a.catgact..~~...............~~....................**

**JN381845.1**  **...........ac.a.catgact..~~...............~~....................**

**JN381846.1**  **...........ac.a.catgact..~~...............~~....................**

**JN381847.1**  **...........ac.a.catgact..~~...............~~....................**

**JN381848.1**  **...........ac.a.catgact..~~...............~~....................**

**JN381849.1**  **...........ac.a.catgact..~~...............~~....................**

**JN381850.1**  **...........ac.a.catgact..~~...............~~....................**

**JN381851.1**  **...........ac.a.catgact..~~...............~~....................**

**JN381852.1**  **...........ac.a.catgact..~~...............~~....................**

**JN381853.1**  **..........-at.a.catgact..~~...............~~....................**

**JN381854.1**  **..........-at.a.catgact..~~...............~~....................**

**JN381855.1**  **..........-at.a.catgact..~~...............~~....................**

**JN381856.1**  **..........-at.a.catgact..~~...............~~....................**

**JN381857.1**  **..........-at.a.catgact..~~...............~~....................**

**JN381858.1**  **..........-at.a.catgact..~~...............~~....................**

**JN381859.1**  **..........-at.a.catgact..~~...............~~....................**

**JN381860.1**  **..........-at.a.catgact..~~...............~~....................**

**JN381861.1**  **..........-at.a.catgact..~~...............~~....................**

**JN381862.1**  **..........-at.a.catgact..~~...............~~....................**

**JN381863.1**  **..........-at.a.catgact..~~...............~~....................**

**JN381864.1**  **..........-at.a.catgact..~~...............~~....................**

**JN381865.1**  **..........-at.a.catgact..~~...............~~....................**

**JN381866.1**  **..........-at.a.catgact..~~...............~~....................**

**JN381867.1**  **..........-at.a.catgact..~~...............~~....................**

**JN381868.1**  **..........-at.a.catgact..~~...............~~....................**

**JN381869.1**  **..........-at.a.catgact..~~...............~~....................**

**JN381870.1**  **..........-at.a.catgact..~~...............~~....................**

**JN381871.1**  **..........-at.a.catgact..~~...............~~....................**

**JN381872.1**  **..........-at.a.catgact..~~...............~~....................**

**JN381873.1**  **..........-at.a.catgact..~~...............~~....................**

**JN604986.1**  **..........-at.a.catgact..~~...............~~....................**

**JN711458.1**  **..........-at.a.catgact..~~...............~~....................**

**JN711459.1**  **..........-at.a.catgact..~~...............~~....................**

**JN864064.1**  **..........-at.a.catgact..~~...............~~....................**

**JQ031753.1**  **...........ac.a.catgact..~~...............~~....................**

**JQ086762.1**  **..........-at.a.catgact..~~...............~~....................**

**JQ086763.1**  **..........-at.a.catgact..~~...............~~....................**

**JX050179.1**  **..........-at.a.catgact..~~...............~~....................**

**JX072965.1**  **..........-at.a.catgact..~~...............~~....................**

**JX131374.1**  **..........-at.a.catgact..~~...............~~....................**

**KC183732.1**  **-----------------atgact..~~...............~~....................**

**KC196115.1**  **...........ac.a.catgact..~~...............~~....................**

**KC517497.1**  **..........-at.a.catgact..~~...............~~....................**

**KC915016.1**  **..........-at.a.catgact..~~...............~~....................**

**KF297915.1**  **...g......-at.a.catgact..~~...............~~....................**

**KF297916.1**  **..........-at.a.catgact..~~...............~~....................**

**KF667310.1**  **..........-at.a.catgact..~~...............~~....................**

**KF667311.1**  **..........-at.a.catgact..~~...............~~....................**

**KF667312.1**  **..........-at.a.catgact..~~...............~~....................**

**KF667313.1**  **..........-at.a.catgact..~~...............~~....................**

**KF667314.1**  **..........-at.a.catgact..~~...............~~....................**

**KF667315.1**  **..........-at.a.catgact..~~...............~~....................**

**KF667316.1**  **...........ac.a.catgact..~~...............~~....................**

**KF667317.1**  **...........ac.a.catgact..~~...............~~....................**

**KF667318.1**  **...........ac.a.catgact..~~...............~~....................**

**KF667319.1**  **...........ac.a.catgact..~~...............~~....................**

**KF667320.1**  **...........ac.a.catgact..~~...............~~....................**

**KF667321.1**  **...........ac.a.catgact..~~...............~~....................**

**KF667322.1**  **...........ac.a.catgact..~~...............~~....................**

**KF667323.1**  **...........ac.a.catgact..~~...............~~....................**

**KF667324.1**  **...........ac.a.catgact..~~...............~~....................**

**KF667325.1**  **...........ac.a.catgact..~~...............~~....................**

**KF667326.1**  **...........ac.a.catgact..~~...............~~....................**

**KF667327.1**  **...........ac.a.catgact..~~...............~~....................**

**KF711994.1**  **-----------------atgact..~~...............~~....................**

**KF907505.1**  **..........-at.a.catgact..~~...............~~....................**

**KM658163.1**  **...........ac.a.catgact..~~...............~~....................**

**KM677246.1**  **..........-a..a.catgact..~~...............~~....................**

**KP164498.2**  **..........-at.a.catgact..~~...............~~..t.................**

**KR265316.1**  **..........-at.a.catgact..~~...............~~....................**

**KT229572.1**  **...........ac.a.catgact..~~...............~~....................**

**KT229573.1**  **...........ac.a.catgact..~~...............~~..............g.....**

**KT229574.1**  **...........ac.a.catgact..~~...............~~....................**

**KT229575.1**  **...........ac.a.catgact..~~...............~~....................**

**KT239164.1**  **..........-at.a.catgact..~~...............~~....................**

**KT957419.1**  **...........ac.a.catgact..~~...............~~....................**

**KT957420.1**  **...........ac.a.catgact..~~...............~~....................**

**KT957421.1**  **...........ac.a.catgact..~~...............~~....................**

**KT957422.1**  **...........ac.a.catgact..~~...............~~....................**

**KT957423.1**  **...........ac.a.catgact..~~...............~~....................**

**KU323483.1**  **..........-at.a.catgact..~~...............~~....................**

**KU351667.1**  **...........ac.a.catgact..~~...............~~....................**

**KU351668.1**  **...........ac.a.catgact..~~...............~~....................**

**KU363309.1**  **..........-at.a.catgact..~~...............~~....................**

**KU508408.1**  **...........ac.a.catgact..~~...............~~....................**

**KU508409.1**  **...........ac.a.catgact..~~...............~~....................**

**KU821122.1**  **-----------------atgact..~~...............~~....................**

**KU871316.1**  **-----------------atgact..~~...............~~....................**

**KU871317.1**  **-----------------atgact..~~...............~~....................**

**KU871318.1**  **-----------------atgact..~~...............~~....................**

**KU871319.1**  **-----------------atgact..~~...............~~....................**

**KU871320.1**  **-----------------atgact..~~...............~~....................**

**KU871321.1**  **-----------------atgact..~~...............~~....................**

**KU871322.1**  **-----------------atgact..~~...............~~....................**

**KU871323.1**  **-----------------atgact..~~...............~~....................**

**KU871324.1**  **-----------------atgact..~~...............~~....................**

**KU871325.1**  **-----------------atgact..~~...............~~....................**

**KU871326.1**  **-----------------atgact..~~...............~~....................**

**KU871327.1**  **-----------------atgact..~~...............~~....................**

**KU871328.1**  **-----------------atgact..~~...............~~....................**

**KU871329.1**  **-----------------atgact..~~...............~~....................**

**KU871330.1**  **-----------------atgact..~~...............~~....................**

**KU871331.1**  **-----------------atgact..~~...............~~....................**

**KU871332.1**  **-----------------atgact..~~...............~~....................**

**KU871333.1**  **-----------------atgact..~~...............~~....................**

**KU871334.1**  **-----------------atgact..~~...............~~....................**

**KU871335.1**  **-----------------atgact..~~...............~~....................**

**KU871336.1**  **-----------------atgact..~~...............~~....................**

**KU871337.1**  **-----------------atgact..~~...............~~....................**

**KU871338.1**  **-----------------atgact..~~...............~~....................**

**KU871339.1**  **-----------------atgact..~~...............~~....................**

**KU871340.1**  **-----------------atgact..~~...............~~....................**

**KU871341.1**  **-----------------atgact..~~...............~~....................**

**KU871342.1**  **-----------------atgact..~~...............~~....................**

**KU871343.1**  **-----------------atgact..~~...............~~....................**

**KU871344.1**  **-----------------atgact..~~...............~~....................**

**KU871345.1**  **-----------------atgact..~~...............~~....................**

**KU871346.1**  **-----------------atgact..~~...............~~....................**

**KU871347.1**  **-----------------atgact..~~...............~~....................**

**KU871348.1**  **-----------------atgact..~~...............~~....................**

**KU871349.1**  **-----------------atgact..~~...............~~....................**

**KU871350.1**  **-----------------atgact..~~...............~~....................**

**KU871351.1**  **-----------------atgact..~~...............~~....................**

**KU871352.1**  **-----------------atgact..~~...............~~....................**

**KU871353.1**  **-----------------atgact..~~...............~~....................**

**KU871354.1**  **-----------------atgact..~~...............~~....................**

**KU871355.1**  **-----------------atgact..~~...............~~....................**

**KU871356.1**  **-----------------atgact..~~...............~~....................**

**KU871357.1**  **-----------------atgact..~~...............~~....................**

**KU871358.1**  **-----------------atgact..~~...............~~....................**

**KU871359.1**  **-----------------atgact..~~...............~~....................**

**KU871360.1**  **-----------------atgact..~~...............~~....................**

**KU871361.1**  **-----------------atgact..~~...............~~....................**

**KU871362.1**  **-----------------atgact..~~...............~~....................**

**KU871363.1**  **-----------------atgact..~~...............~~....................**

**KU871364.1**  **-----------------atgact..~~...............~~....................**

**KU871365.1**  **-----------------atgact..~~...............~~....................**

**KU871366.1**  **-----------------atgact..~~...............~~....................**

**KU871367.1**  **-----------------atgact..~~...............~~....................**

**KU871368.1**  **-----------------atgact..~~...............~~....................**

**KU871369.1**  **-----------------atgact..~~...............~~....................**

**KU871370.1**  **-----------------atgact..~~...............~~....................**

**KU871371.1**  **-----------------atgact..~~...............~~....................**

**KX357114.1**  **...........ac.a.catgact..~~...............~~....................**

**KX779520.1**  **..-.......-at.a.catgact..~~...............~~....................**

**KX779521.1**  **..-.......-at.a.catgact..~~...............~~....................**

**KX779522.1**  **..-.......-at.a.catgact..~~...............~~....................**

**L48961.1**  **..........-at.a.catgact..~~...............~~....................**

**L78128.1**  **..........-at.a.catgact..~~...............~~....................**

**M18370.1**  **..........-at.a.catgact..~~...............~~....................**

**M55506.1**  **..........-at.a.catgact..~~...............~~....................**

**NC_001437.1**  **..........-at.a.catgact..~~...............~~....................**

**JN644310.1**  **..........-at.a.catgact..~~...............~~....................**

**U14163.1**  **..........-at.a.catgact..~~...............~~....................**

**U15763.1**  **..........-at.a.catgact..~~...............~~....................**

**U47032.1**  **..........-at.a.catgact..~~...............~~....................**

**5) Barros assay (2013)**

Accession Numbers: 3548 3682

....|....|....|....|....|....|....|....|....|....|....|....|....|....

**CCTTTTCAGYTGGGCCTTCT~~GTGACCATTCCTGCGGTTTTGGGG~~TTGGGGGYATBACYTACACTG**

**EF571853.1**  **....................~~.....t..................~~.....................**

**AB051292.1**  **....................~~.....g..................~~.....................**

**AB196923.1**  **....................~~........................~~.....................**

**AB196924.1**  **....................~~........................~~.....................**

**AB196925.1**  **....................~~........................~~.....................**

**AB196926.1**  **....................~~........................~~.....................**

**AB241118.1**  **....................~~.....g..................~~.....................**

**AB241119.1**  **....................~~.....g..................~~.....................**

**AB269326.1**  **....................~~........................~~.....................**

**AB471666.2**  **....................~~.....g.....c............~~.....................**

**AB471667.2**  **....................~~.....g.....c............~~.....................**

**AB471668.2**  **....................~~.....g.....c............~~.....................**

**AB471669.2**  **....................~~.....g..................~~.....................**

**AB471670.2**  **....................~~.....g..................~~.....................**

**AB551990.1**  **....................~~........................~~.....................**

**AB551991.1**  **....................~~........................~~..................t..**

**AB551992.1**  **....................~~........................~~..................t..**

**AB569980.2**  **....................~~........................~~.....................**

**AB569981.2**  **....................~~........................~~.....................**

**AB569982.2**  **....................~~........................~~.....................**

**AB569983.2**  **....................~~........................~~.....................**

**AB569984.2**  **....................~~........................~~.....................**

**AB569985.2**  **....................~~........................~~.....................**

**AB569987.2**  **....................~~........................~~.....................**

**AB569988.2**  **....................~~........................~~.....................**

**AB569989.2**  **....................~~........................~~.....................**

**AB569990.2**  **....................~~........................~~.....................**

**AB594829.1**  **....................~~.....g..................~~.....................**

**AB698905.1**  **....................~~.....g..................~~.....................**

**AB698906.1**  **....................~~.....g..................~~.....................**

**AB698907.1**  **....................~~.....g..................~~.....................**

**AB698908.1**  **....................~~.....g..................~~.....................**

**AB698909.1**  **....................~~.....g..................~~.....................**

**AB830335.1**  **....................~~.....g..................~~.....................**

**AB853904.1**  **....................~~.....g..................~~.....................**

**AB920399.1**  **....................~~.....g..................~~.....................**

**AB981183.1**  **....................~~.....g..................~~.....................**

**AB981184.1**  **....................~~.....g..................~~.....................**

**AF014160.1**  **....................~~.......................a~~.....................**

**AF014161.1**  **....................~~.......................a~~.....................**

**AF045551.2**  **....................~~.....g..................~~.....................**

**AF069076.1**  **....................~~........................~~.....................**

**AF075723.1**  **....................~~........................~~.....................**

**AF080251.1**  **....................~~........................~~.....................**

**AF098735.1**  **....................~~........................~~.....................**

**AF098736.1**  **.........a..........~~........................~~.....................**

**AF098737.1**  **....................~~........................~~.....................**

**AF217620.1**  **....................~~.....ag.................~~.....................**

**AF221499.1**  **....................~~.....t..................~~.....................**

**AF221500.1**  **....................~~.....t..................~~.....................**

**AF254452.1**  **....................~~........................~~.....................**

**AF254453.1**  **....................~~........................~~.....................**

**AF315119.1**  **....................~~........................~~.....................**

**AF416457.1**  **....................~~........................~~.....................**

**AF486638.1**  **....................~~........................~~.....................**

**AY184212.1**  **....................~~.....ag.................~~.............g.......**

**AY303791.1**  **....................~~........................~~.....................**

**AY303792.1**  **....................~~........................~~.....................**

**AY303793.1**  **....................~~.....t..a...............~~.....................**

**AY303794.1**  **....................~~.....t..a...............~~.....................**

**AY303795.1**  **....................~~........................~~.....................**

**AY303796.1**  **....................~~........................~~.....................**

**AY303797.1**  **....................~~........................~~.....................**

**AY303798.1**  **....................~~........................~~.....................**

**AY316157.1**  **....................~~.....g..................~~.....................**

**AY508812.1**  **....................~~........................~~.....................**

**AY508813.1**  **....................~~........................~~.....................**

**AY585242.1**  **....................~~........................~~.....................**

**AY585243.1**  **....................~~........................~~.....................**

**AY849939.1**  **....................~~........................~~.....................**

**D90194.1**  **....................~~........................~~.....................**

**D90195.1**  **....................~~........................~~.....................**

**DD298237.1**  **....................~~........................~~.....................**

**DD298243.1**  **....................~~........................~~.....................**

**DD367827.1**  **....................~~........................~~.....................**

**DD367828.1**  **....................~~........................~~.....................**

**DI075091.1**  **....................~~........................~~.....................**

**DI085220.1**  **....................~~........................~~.....................**

**EF107523.1**  **....................~~........................~~.....................**

**EF543861.1**  **....................~~........................~~.....................**

**EF623987.1**  **....................~~........................~~.....................**

**EF623988.1**  **....................~~........................~~.....................**

**EF623989.1**  **....................~~........................~~.....................**

**EU429297.1**  **....................~~.....g..................~~.....................**

**EU693899.1**  **....................~~.....g..................~~.....................**

**EU880214.1**  **....................~~.....g..................~~.....................**

**FJ185036.1**  **....................~~.....t..................~~................t....**

**FJ185037.1**  **....................~~.....t..................~~................t....**

**FJ495189.1**  **....................~~.....g..................~~.....................**

**GQ199609.1**  **....................~~........................~~.....................**

**GQ902058.1**  **....................~~.....g..................~~.....................**

**GQ902059.1**  **....................~~.....g..................~~.....................**

**GQ902060.1**  **....................~~.....g..................~~.....................**

**GQ902061.1**  **....................~~.....g.................a~~.....................**

**GQ902062.1**  **....................~~.....g..................~~.....................**

**GQ902063.1**  **....................~~........................~~.....................**

**GQ918133.2**  **....................~~........................~~.....................**

**GU187972.1**  **....................~~.....g..................~~.....................**

**GU205163.1**  **....................~~.....g..................~~.....................**

**GU556217.1**  **....................~~.....g..................~~.....................**

**HE861351.1**  **....................~~.....t..................~~.....................**

**HM228921.1**  **....................~~.....g..................~~.....................**

**HM366552.1**  **....................~~.....g..................~~.....................**

**HM596272.1**  **....................~~..a..g..c...............~~.....................**

**HQ223285.1**  **....................~~.....ag.................~~.....................**

**HQ223286.1**  **....................~~.....ag.................~~.....................**

**HQ223287.1**  **....................~~.....ag.................~~.....................**

**HQ652538.1**  **....................~~.....g..................~~.....................**

**HQ893545.1**  **....................~~.....g..................~~.....................**

**JF499788.1**  **....................~~.....g..................~~.....................**

**JF499789.1**  **....................~~.....g..................~~.....................**

**JF499790.1**  **....................~~.....g..................~~.....................**

**JF706267.1**  **....................~~.....g..................~~.....................**

**JF706268.1**  **....................~~.....g..................~~.....................**

**JF706269.1**  **....................~~........................~~.....................**

**JF706270.1**  **....................~~.....g..................~~.....................**

**JF706271.1**  **....................~~.....g..................~~.....................**

**JF706272.1**  **....................~~........................~~.....................**

**JF706273.1**  **....................~~........................~~.....................**

**JF706274.1**  **....................~~.....g..................~~.....................**

**JF706275.1**  **....................~~........................~~.....................**

**JF706276.1**  **....................~~........................~~.....................**

**JF706277.1**  **....................~~.....g..................~~.....................**

**JF706278.1**  **....................~~.....g..................~~.....................**

**JF706279.1**  **....................~~.....g..................~~..................t..**

**JF706280.1**  **....................~~........................~~.....................**

**JF706281.1**  **....................~~.....g..................~~.....................**

**JF706282.1**  **....................~~.....g..................~~..................t..**

**JF706283.1**  **....................~~........................~~.....................**

**JF706284.1**  **....................~~.....t..................~~................t....**

**JF706285.1**  **....................~~.....t..................~~................t....**

**JF706286.1**  **....................~~.....g..................~~.....................**

**JF915894.1**  **....................~~.....a..................~~.....................**

**JN381830.1**  **....................~~.....g..................~~.....................**

**JN381831.1**  **....................~~.....g..................~~.....................**

**JN381832.1**  **....................~~.....g..................~~.....................**

**JN381833.1**  **....................~~.....g..................~~.....................**

**JN381834.1**  **....................~~.....g..................~~.....................**

**JN381835.1**  **....................~~.....g..................~~.....................**

**JN381836.1**  **....................~~.....g..................~~.....................**

**JN381837.1**  **....................~~.....g..................~~.....................**

**JN381838.1**  **....................~~.....g..................~~.....................**

**JN381839.1**  **....................~~.....g..................~~.....................**

**JN381840.1**  **....................~~.....g..................~~.....................**

**JN381841.1**  **....................~~.....g..................~~.....................**

**JN381842.1**  **....................~~.....g..................~~.....................**

**JN381843.1**  **....................~~.....g..................~~.....................**

**JN381844.1**  **....................~~.....g..................~~.....................**

**JN381845.1**  **....................~~.....g..................~~.....................**

**JN381846.1**  **....................~~.....g..................~~.....................**

**JN381847.1**  **....................~~.....g..................~~.....................**

**JN381848.1**  **....................~~.....g..................~~.....................**

**JN381849.1**  **....................~~.....g..................~~.....................**

**JN381850.1**  **....................~~.....g..................~~.....................**

**JN381851.1**  **....................~~.....g..................~~.....................**

**JN381852.1**  **....................~~.....g..................~~.....................**

**JN381853.1**  **....................~~........................~~........g............**

**JN381854.1**  **....................~~........................~~.....................**

**JN381855.1**  **....................~~........................~~.....................**

**JN381856.1**  **....................~~........................~~.....................**

**JN381857.1**  **....................~~........................~~.....................**

**JN381858.1**  **....................~~........................~~.....................**

**JN381859.1**  **....................~~........................~~.....................**

**JN381860.1**  **....................~~........................~~.....................**

**JN381861.1**  **....................~~........................~~.....................**

**JN381862.1**  **....................~~........................~~.....................**

**JN381863.1**  **....................~~........................~~.....................**

**JN381864.1**  **....................~~........................~~.....................**

**JN381865.1**  **....................~~........................~~.....................**

**JN381866.1**  **....................~~........................~~.....................**

**JN381867.1**  **....................~~........................~~.....................**

**JN381868.1**  **....................~~........................~~.....................**

**JN381869.1**  **....................~~........................~~.....................**

**JN381870.1**  **....................~~........................~~.....................**

**JN381871.1**  **....................~~........................~~.....................**

**JN381872.1**  **....................~~........................~~.....................**

**JN381873.1**  **....................~~........................~~.....................**

**JN604986.1**  **....................~~........................~~.....................**

**JN711458.1**  **.....c..............~~.....t..................~~.....................**

**JN711459.1**  **.....c..............~~.....t..................~~.....................**

**JN864064.1**  **....................~~........................~~.....................**

**JQ031753.1**  **....................~~.....g..................~~.....................**

**JQ086762.1**  **....................~~........................~~.....................**

**JQ086763.1**  **....................~~........................~~.....................**

**JX050179.1**  **....................~~........................~~.....................**

**JX072965.1**  **....................~~........................~~.....................**

**JX131374.1**  **..............t.....~~........................~~.....................**

**KC183732.1**  **....................~~........................~~.....................**

**KC196115.1**  **....................~~.....g..................~~.....................**

**KC517497.1**  **....................~~........................~~.....................**

**KC915016.1**  **....................~~........................~~.....................**

**KF297915.1**  **....................~~........................~~.....................**

**KF297916.1**  **....................~~........................~~.....................**

**KF667310.1**  **....................~~.....t..a...............~~.....................**

**KF667311.1**  **....................~~.....t..a...............~~.....................**

**KF667312.1**  **....................~~........................~~.....................**

**KF667313.1**  **....................~~........................~~.....................**

**KF667314.1**  **....................~~.....t..a...............~~.....................**

**KF667315.1**  **....................~~.....t..a...............~~.....................**

**KF667316.1**  **....................~~.....g..................~~.....................**

**KF667317.1**  **....................~~.....g..................~~.....................**

**KF667318.1**  **....................~~.....g..................~~.....................**

**KF667319.1**  **....................~~.....g..................~~.....................**

**KF667320.1**  **....................~~.....g..................~~.....................**

**KF667321.1**  **....................~~.....g..................~~.....................**

**KF667322.1**  **....................~~.....g..................~~.....................**

**KF667323.1**  **....................~~.....g..................~~.....................**

**KF667324.1**  **....................~~.....g..................~~.....................**

**KF667325.1**  **....................~~.....g..........c.......~~.....................**

**KF667326.1**  **....................~~.....g..................~~.....................**

**KF667327.1**  **....................~~.....g..................~~.....................**

**KF711994.1**  **....................~~........................~~.....................**

**KF907505.1**  **....................~~.......................a~~.....................**

**KM658163.1**  **....................~~.....g..................~~.....................**

**KM677246.1**  **....................~~..a..g..c...............~~.....................**

**KP164498.2**  **....................~~........................~~.....................**

**KR265316.1**  **....................~~........................~~.....................**

**KT229572.1**  **....................~~.....g..................~~.....................**

**KT229573.1**  **....................~~.....g..................~~.....................**

**KT229574.1**  **....................~~.....g..................~~.....................**

**KT229575.1**  **....................~~.....g..................~~.....................**

**KT239164.1**  **..............t.....~~........................~~.....................**

**KT957419.1**  **.tc.................~~.....g..................~~.....................**

**KT957420.1**  **....................~~.....g..................~~.....................**

**KT957421.1**  **....................~~.....g..................~~.....................**

**KT957422.1**  **....................~~.....g..................~~..................t..**

**KT957423.1**  **....................~~.....g..................~~..................t..**

**KU323483.1**  **....................~~........................~~.....................**

**KU351667.1**  **....................~~.....g..................~~.....................**

**KU351668.1**  **....................~~.....g........a.........~~.....................**

**KU363309.1**  **....................~~........................~~.....................**

**KU508408.1**  **....................~~.....g..................~~.....................**

**KU508409.1**  **....................~~.....g........a.........~~.....................**

**KU821122.1**  **....................~~........................~~.....................**

**KU871316.1**  **....................~~........................~~.....................**

**KU871317.1**  **....................~~........................~~.....................**

**KU871318.1**  **....................~~........................~~.....................**

**KU871319.1**  **....................~~........................~~.....................**

**KU871320.1**  **....................~~........................~~.....................**

**KU871321.1**  **....................~~........................~~.....................**

**KU871322.1**  **....................~~........................~~.....................**

**KU871323.1**  **....................~~........................~~.....................**

**KU871324.1**  **....................~~........................~~.....................**

**KU871325.1**  **....................~~........................~~.....................**

**KU871326.1**  **....................~~........................~~.....................**

**KU871327.1**  **....................~~........................~~.....................**

**KU871328.1**  **....................~~........................~~.....................**

**KU871329.1**  **....................~~........................~~.....................**

**KU871330.1**  **....................~~........................~~.....................**

**KU871331.1**  **....................~~........................~~.....................**

**KU871332.1**  **....................~~........................~~.....................**

**KU871333.1**  **....................~~........................~~.....................**

**KU871334.1**  **....................~~........................~~.....................**

**KU871335.1**  **....................~~........................~~.....................**

**KU871336.1**  **....................~~........................~~.....................**

**KU871337.1**  **....................~~........................~~.....................**

**KU871338.1**  **....................~~........................~~.....................**

**KU871339.1**  **....................~~........................~~.....................**

**KU871340.1**  **....................~~........................~~.....................**

**KU871341.1**  **....................~~........................~~.....................**

**KU871342.1**  **....................~~........................~~.....................**

**KU871343.1**  **....................~~................c.......~~.....................**

**KU871344.1**  **....................~~........................~~.....................**

**KU871345.1**  **....................~~........................~~.....................**

**KU871346.1**  **....................~~........................~~.....................**

**KU871347.1**  **....................~~........................~~.....................**

**KU871348.1**  **....................~~........................~~.....................**

**KU871349.1**  **....................~~........................~~.....................**

**KU871350.1**  **....................~~........................~~.....................**

**KU871351.1**  **....................~~........................~~.....................**

**KU871352.1**  **....................~~........................~~.....................**

**KU871353.1**  **....................~~........................~~.....................**

**KU871354.1**  **....................~~........................~~.....................**

**KU871355.1**  **....................~~........................~~.....................**

**KU871356.1**  **....................~~........................~~.....................**

**KU871357.1**  **....................~~........................~~.....................**

**KU871358.1**  **....................~~........................~~.....................**

**KU871359.1**  **....................~~........................~~.....................**

**KU871360.1**  **....................~~........................~~.....................**

**KU871361.1**  **....................~~........................~~.....................**

**KU871362.1**  **....................~~........................~~.....................**

**KU871363.1**  **....................~~........................~~.....................**

**KU871364.1**  **....................~~........................~~.....................**

**KU871365.1**  **....................~~........................~~.....................**

**KU871366.1**  **....................~~........................~~.....................**

**KU871367.1**  **....................~~........................~~.....................**

**KU871368.1**  **....................~~........................~~.....................**

**KU871369.1**  **....................~~........................~~.....................**

**KU871370.1**  **....................~~........................~~.....................**

**KU871371.1**  **....................~~........................~~.....................**

**KX357114.1**  **....................~~.....g..................~~.....................**

**KX779520.1**  **....................~~........................~~.....................**

**KX779521.1**  **....................~~........................~~.....................**

**KX779522.1**  **....................~~........................~~.....................**

**L48961.1**  **....................~~........................~~.....................**

**L78128.1**  **....................~~........................~~.....................**

**M18370.1**  **....................~~........................~~.....................**

**M55506.1**  **....................~~........................~~.....................**

**NC_001437.1**  **....................~~........................~~.....................**

**JN644310.1**  **....................~~........................~~.....................**

**U14163.1**  **....................~~........................~~.....................**

**U15763.1**  **....................~~........................~~.....................**

**U47032.1**  **....................~~........................~~.....................**

**6) Huang assay (2004)**

Accession Numbers: 5357 5452

....|....|....|....|....|....|....|....|....|....|....|....|....|....|....|

**AGAGCACCAAGGGAATGAAATAGT~~CCACGCCACTCGACCCATAGACTG~~AGAGTGCCCAACTACAACCTATT**

**EF571853.1**  **........................~~...t.......tga..cacagact~~..............t........**

**AB051292.1**  **g.....t..g..a...........~~...t.......tga..catagact~~..g..............tt....**

**AB196923.1**  **........................~~...........tga..catagact~~.......................**

**AB196924.1**  **........................~~...........tga..catagact~~.......................**

**AB196925.1**  **........................~~...........tga..catagact~~.......................**

**AB196926.1**  **........................~~...........tga..catagact~~.......................**

**AB241118.1**  **g.....t..g..a...........~~...t.......tga..catagact~~..g..............tt....**

**AB241119.1**  **g.....t..g..a...........~~...t.......tga..catagact~~..g..............tt....**

**AB269326.1**  **........................~~...........tga..catagact~~.......................**

**AB471666.2**  **g.....t..g..a...........~~...t.......tga..cataga.t~~..g..............tt....**

**AB471667.2**  **g.....t..g..a...........~~...t.......tga..cataga.t~~..g..............tt....**

**AB471668.2**  **g.....t..g..a...........~~...t.......tga..cataga.t~~..g..............tt....**

**AB471669.2**  **g.....t..g..a...........~~...t.......tga..cataga.t~~..g..............tt....**

**AB471670.2**  **g.....t..g..a...........~~...t.......tga..cataga.t~~..g..............tt....**

**AB551990.1**  **........................~~...........tga..catagact~~.......................**

**AB551991.1**  **........................~~...........tga..cacaga.t~~..............t........**

**AB551992.1**  **........................~~...t.......tga..cacaga.t~~..............t........**

**AB569980.2**  **............a...........~~...........tga..catagact~~.......................**

**AB569981.2**  **............a...........~~...........tga..catagact~~.......................**

**AB569982.2**  **........................~~...........tga..catagact~~.......................**

**AB569983.2**  **........................~~...........tga..catagact~~.......................**

**AB569984.2**  **........................~~...........tga..catagact~~.......................**

**AB569985.2**  **........................~~...........tga..catagact~~.......................**

**AB569987.2**  **........................~~...........tga..catagact~~.......................**

**AB569988.2**  **............a...........~~...........tga..catagact~~.......................**

**AB569989.2**  **........................~~...........tga..catagact~~.......................**

**AB569990.2**  **........................~~...........tga..catagact~~.......................**

**AB594829.1**  **g..a..t..g..a...........~~...t.......tga..catagact~~.................tt....**

**AB698905.1**  **g.....t..g..a...........~~...t.......tga..catagact~~..g..............tt....**

**AB698906.1**  **g.....t..g..a...........~~...t.......tga..catagact~~..g..............tt....**

**AB698907.1**  **g.....t..g..a...........~~...t.......tga..catagact~~..g..............tt....**

**AB698908.1**  **g.....t..g..a...........~~...t.......tga..catagact~~..g..............tt....**

**AB698909.1**  **g.....t..g..a...........~~...t.......tga..catagact~~..g..............tt....**

**AB830335.1**  **g.....t..g..a...........~~...t.......tga..catagact~~...........t......t....**

**AB853904.1**  **g.....t..g..a...........~~...t.......tga..catagact~~..g..............tt....**

**AB920399.1**  **g.....t..g..a...........~~...t.......tga..catagact~~..g..............tt....**

**AB981183.1**  **g.....t..g..a...........~~...t.......tga..catagact~~...........t......t....**

**AB981184.1**  **g.....t..g..a...........~~...t.......tga..catagact~~...........t......t....**

**AF014160.1**  **........................~~...........tga..catagact~~.......................**

**AF014161.1**  **........................~~...........tga..catagact~~.......................**

**AF045551.2**  **g.....t..g..a...........~~...t.......tga..catagact~~..g..............tt....**

**AF069076.1**  **........................~~...........tga..catagact~~.......................**

**AF075723.1**  **........................~~...t.......tga..catagact~~.......................**

**AF080251.1**  **........................~~t..........tga..cacagact~~..................t....**

**AF098735.1**  **........................~~...........tga..catagact~~.......................**

**AF098736.1**  **........................~~...........tga..catagact~~.......................**

**AF098737.1**  **........................~~...........tga..catagact~~.......................**

**AF217620.1**  **g.....t..g..............~~...t..a....tga.tcacagact~~...........t......t.g..**

**AF221499.1**  **........................~~...........tga..catagact~~.......................**

**AF221500.1**  **........................~~...........tga..catagact~~.......................**

**AF254452.1**  **........................~~...........tga..catagact~~.......................**

**AF254453.1**  **........................~~...........tga..catagact~~.......................**

**AF315119.1**  **........................~~...........tga..catagact~~.......................**

**AF416457.1**  **........................~~...........tga..catagact~~.......................**

**AF486638.1**  **.................c......~~...........tga..catagact~~.......................**

**AY184212.1**  **g.....t.....a.....g.....~~...t.....cttga..cacagact~~..g.....t..t........g..**

**AY303791.1**  **........................~~...........tga..catagact~~.......................**

**AY303792.1**  **........................~~...........tga..catagact~~.......................**

**AY303793.1**  **........................~~...........tga..catagact~~.......................**

**AY303794.1**  **........................~~...........tga..catagact~~.......................**

**AY303795.1**  **........................~~...........tga..catagact~~.......................**

**AY303796.1**  **........................~~...........tga..catagact~~.......................**

**AY303797.1**  **........................~~...........tga..catagact~~.......................**

**AY303798.1**  **........................~~...........tga..catagact~~.......................**

**AY316157.1**  **g.....t..g..a...........~~...t.......tga..catagact~~..g..............tt....**

**AY508812.1**  **........................~~...........tga..catagact~~.......................**

**AY508813.1**  **........................~~...........tga..catagact~~.......................**

**AY585242.1**  **........................~~...........tga..cacaga.t~~.................t.....**

**AY585243.1**  **........................~~...........tga..cacaga.t~~.................t..g..**

**AY849939.1**  **......t.................~~...........tga..catagact~~..............t........**

**D90194.1**  **........................~~...........tga..catagact~~.......................**

**D90195.1**  **........................~~...........tga..catagact~~.......................**

**DD298237.1**  **........................~~...........tga..cacaga.t~~.................t.....**

**DD298243.1**  **........................~~...........tga..cacaga.t~~.................t.....**

**DD367827.1**  **........................~~...........tga..catagact~~.......................**

**DD367828.1**  **........................~~...........tga..catagact~~.......................**

**DI075091.1**  **........................~~...........tga..cacaga.t~~.................t.....**

**DI085220.1**  **........................~~...........tga..cacaga.t~~.................t.....**

**EF107523.1**  **......t.................~~...........tga..catagact~~..............t........**

**EF543861.1**  **........................~~...........tga..catagact~~.......................**

**EF623987.1**  **........................~~...t.......tga..catagact~~.......................**

**EF623988.1**  **........................~~...t.......tga..catagact~~.......................**

**EF623989.1**  **........................~~...t.......tga..catagact~~.......................**

**EU429297.1**  **g.....t..g..a...........~~...t.......tga..catagact~~...........t......t....**

**EU693899.1**  **g.....t..g..a...........~~t..t.......tga..catagact~~...........t......t....**

**EU880214.1**  **g.....t..g..a...........~~...t.......tga..catagact~~...........t......t....**

**FJ185036.1**  **........................~~...t.......tga..cacagact~~.......................**

**FJ185037.1**  **........................~~...t.......tga..cacagact~~.......................**

**FJ495189.1**  **g.....t..g..a...........~~...t.......tga..catagact~~..g..............tt....**

**GQ199609.1**  **........................~~...........tga..cacaga.t~~.................t..g..**

**GQ902058.1**  **g.....t..g..............~~t..t.....c.tga..cataggct~~.................tt....**

**GQ902059.1**  **g.....t..g..a...........~~t..t..t..c.tga..cataggct~~.................tt....**

**GQ902060.1**  **g.....t..g..a...........~~t..t..t..c.tga..catagact~~.................tt....**

**GQ902061.1**  **g.....t..g..a...........~~t..t..t..c.tga..catagact~~...........t.....tt....**

**GQ902062.1**  **g.....t..g..a...........~~t..t..t..c.tga..catagact~~.................tt....**

**GQ902063.1**  **........................~~...t.......tga..catagact~~........t..............**

**GQ918133.2**  **.tc.....................~~...........tga..catagact~~.......................**

**GU187972.1**  **g.....t..g..a...........~~...t.......tga..catagact~~..g..............tt....**

**GU205163.1**  **g.....t..g..a...........~~...t.......tga..catagact~~..g...............t....**

**GU556217.1**  **g.....t..g..a...........~~...t.......tga..catagact~~...........t......t....**

**HE861351.1**  **........................~~...t.......tga..cacagact~~..............t........**

**HM228921.1**  **g.....t..g..a...........~~...t.......tga..catagact~~..g..............tt....**

**HM366552.1**  **g.....t..g..a...........~~...t..t....tga..catagact~~..g..............tt....**

**HM596272.1**  **g..a.....g..a.....g.....~~t..t.......t.a.gcatagact~~c.c..t.....t......t.g..**

**HQ223285.1**  **g.....t..g..............~~...t.......tga..cacagact~~..................t....**

**HQ223286.1**  **g.....t..g..............~~...........tga.tcacagact~~...........t......t.g..**

**HQ223287.1**  **g.....t..g.....c........~~...t.......tga.tcacagact~~..................t.g..**

**HQ652538.1**  **...a..t..g..a...........~~...t.......tga..catagact~~..g..............tt....**

**HQ893545.1**  **g.....t..g..a...........~~...t.......tga..catagact~~...........t......t....**

**JF499788.1**  **g.....t..g..a...........~~...t.......tga..catagact~~...........t......t....**

**JF499789.1**  **g.....t..g..a...........~~...t.......tga..catagact~~...........t......t....**

**JF499790.1**  **......t..g..a...........~~...t.......tga..catagact~~..g...............t....**

**JF706267.1**  **g.....t..g..a...........~~...t.......tga..catagact~~..g..............tt....**

**JF706268.1**  **g..a..t..g..a...........~~...t.......tga..catagact~~..g..............tt....**

**JF706269.1**  **........................~~...........tga..catagact~~.......................**

**JF706270.1**  **g.....t..g..a...........~~t..t.......tga..catagact~~..g..............tt....**

**JF706271.1**  **g.....t..g..a...........~~...t.......tga..catagact~~..g..............tt....**

**JF706272.1**  **......t.................~~...........tga..catagact~~..............t........**

**JF706273.1**  **......t.................~~...........tga..catagact~~..............t........**

**JF706274.1**  **g.....t..g..a...........~~...t.......tga..catagact~~..g..............tt....**

**JF706275.1**  **........................~~...........tga..catagact~~.......................**

**JF706276.1**  **......t.................~~...........tga..catagact~~..............t........**

**JF706277.1**  **g.....t..g..a...........~~...t.......tga..catagact~~..g...............t....**

**JF706278.1**  **g.....t..g..a...........~~...t.......tga..catagact~~..g........t.....tt....**

**JF706279.1**  **g.....t..g..a...........~~t..t..t....tga..catagact~~.................tt....**

**JF706280.1**  **........................~~...........tga..catagact~~.......................**

**JF706281.1**  **g.....t..g..a...........~~...t.......tga..catagact~~.................tt....**

**JF706282.1**  **g.....t..g..a...........~~...t.......tga..catagact~~..g..............tt....**

**JF706283.1**  **........................~~...........tga..catagact~~..............t........**

**JF706284.1**  **........................~~...t.......tga..cacagact~~.......................**

**JF706285.1**  **........................~~...t.......tga..cacagact~~.......................**

**JF706286.1**  **g.....t..g..a...........~~...t.......tga..catagact~~...........t......t....**

**JF915894.1**  **...a..t..g........g.....~~t.........tt.a.gcataggct~~c.c..t............t.g..**

**JN381830.1**  **g.....t..g..a...........~~...t.......tga..catagact~~..g..............tt....**

**JN381831.1**  **g.....t..g..a...........~~...t.......tga..catagact~~..g..............tt....**

**JN381832.1**  **g.....t..g..a...........~~t..t.......tga..catagact~~..g..............tt....**

**JN381833.1**  **g..a..t..g..a...........~~...t.......tga..catagact~~..g..............tt....**

**JN381834.1**  **g.....t..g..a...........~~...t.......tga..catagact~~..g...............t....**

**JN381835.1**  **g.....t..g..a...........~~...t.......tga..catagact~~..g........t.....tt....**

**JN381836.1**  **g.....t..g..a...........~~...t.......tga..catagact~~..g..............tt....**

**JN381837.1**  **g.....t..g..a...........~~t..t.......tga..catagact~~..g..............tt....**

**JN381838.1**  **......t..g..a...........~~...t.......tga..catagact~~..g..............tt....**

**JN381839.1**  **g.....t..g..a...........~~...t.......tga..catagact~~..g..............tt....**

**JN381840.1**  **g.....t..g..a...........~~...t.......tga..catagact~~..g..............tt....**

**JN381841.1**  **g.....t..g..a...........~~...t.......tga..catagact~~..g..............tt....**

**JN381842.1**  **g.....t..g..a...........~~...t.......tga..catagact~~..g..............tt....**

**JN381843.1**  **g.....t..g..a...........~~...t.......tga..catagact~~..g..............tt....**

**JN381844.1**  **g.....t..g..a...........~~t..t.......tga..catagact~~..g..............tt....**

**JN381845.1**  **g.....t..g..a...........~~t..t.......tga..catagact~~..g..............tt....**

**JN381846.1**  **g.....t..g..a...........~~...t.......tga..catagact~~..g..............tt....**

**JN381847.1**  **g.....t..g..a...........~~...t.......tga..catagact~~..g........t.....tt....**

**JN381848.1**  **g..a..t..g..a...........~~...t.......tga..catagact~~..g..............tt....**

**JN381849.1**  **g.....t..g..a...........~~...t.......tga..catagact~~...........t.....tt....**

**JN381850.1**  **g.....t..g..a.....g.....~~...t.......tga..catagact~~..g..............tt....**

**JN381851.1**  **g........g..a...........~~...t.......tga..catagact~~..g..............tt....**

**JN381852.1**  **g.....t..g..a...........~~...t.......tga..catagact~~..g..............tt....**

**JN381853.1**  **......t.................~~...........tga..catagact~~..............t........**

**JN381854.1**  **......t.................~~...........tga..catagact~~..............t........**

**JN381855.1**  **......t.................~~...........tga..catagact~~..............t........**

**JN381856.1**  **......t.................~~...........tga..catagact~~..............t........**

**JN381857.1**  **......t.................~~...........tga..catagact~~..............t........**

**JN381858.1**  **......t.................~~...........tga..catagact~~..............t........**

**JN381859.1**  **......t.................~~...........tga..catagact~~..............t........**

**JN381860.1**  **......t.................~~...........tga..catagact~~..............t........**

**JN381861.1**  **......t.....a...........~~...t.......tga..catagact~~..............t........**

**JN381862.1**  **......t.................~~...........tga..catagact~~.......................**

**JN381863.1**  **......t.................~~...........tga..catagact~~..............t........**

**JN381864.1**  **........................~~...........tga..catagact~~..............t........**

**JN381865.1**  **........................~~...........tga..catagact~~..............t........**

**JN381866.1**  **........................~~...........tga..catagact~~..............t........**

**JN381867.1**  **........................~~...........tga..catagact~~.......................**

**JN381868.1**  **......t.................~~...........tga..catagact~~..............t........**

**JN381869.1**  **........................~~...........tga..catagact~~.......................**

**JN381870.1**  **........................~~...........tga..catagact~~...........t...........**

**JN381871.1**  **........................~~...........tga..catagact~~.......................**

**JN381872.1**  **........................~~...........tga..cacagact~~.......................**

**JN381873.1**  **........................~~...........tga..cacaga.t~~..............t........**

**JN604986.1**  **........................~~...........tga..catagact~~.......................**

**JN711458.1**  **........................~~...t.......tga.tcacagact~~..............t........**

**JN711459.1**  **........................~~...t.......tga.tcacagact~~..............t........**

**JN864064.1**  **........................~~...........tga..catagact~~.......................**

**JQ031753.1**  **g.....t..g..a...........~~...t.......tga..catagact~~..g...............t....**

**JQ086762.1**  **........................~~...........tga..catagact~~.......................**

**JQ086763.1**  **........................~~...........tga..catagact~~.......................**

**JX050179.1**  **........................~~t..........tga..cacagact~~..................t....**

**JX072965.1**  **........................~~t..........tga..cacagact~~..................t....**

**JX131374.1**  **........................~~t..........tga..cacagact~~..................t....**

**KC183732.1**  **......t.................~~...........tga..catagact~~..............t........**

**KC196115.1**  **g.....t..g..a...........~~...t.......tga..catagact~~..g..............tt....**

**KC517497.1**  **........................~~...........tga..catagact~~.......................**

**KC915016.1**  **...a....................~~...........tga..cacaga.t~~.......................**

**KF297915.1**  **........................~~...........tga..catagact~~.......................**

**KF297916.1**  **...a....................~~...........tga..cacaga.t~~.......................**

**KF667310.1**  **........................~~...........tga..catagact~~.......................**

**KF667311.1**  **........................~~...........tga..catagact~~.......................**

**KF667312.1**  **........................~~...........tga..catagact~~.......................**

**KF667313.1**  **........................~~...........tga..catagact~~.......................**

**KF667314.1**  **........................~~...........tga..catagact~~.......................**

**KF667315.1**  **........................~~...........tga..catagact~~.......................**

**KF667316.1**  **g.....t..g..a...........~~...t.......tga..catagact~~...........t......t....**

**KF667317.1**  **g.....t..g..a...........~~...t.......tga..catagact~~...........t......t....**

**KF667318.1**  **g.....t..g..a...........~~...t.......tga..catagact~~..g...............t....**

**KF667319.1**  **......t..g..a...........~~...t.......tga..catagact~~..g...............t....**

**KF667320.1**  **g.....t..g..a...........~~...t.....a.tga..catagact~~..g...............t....**

**KF667321.1**  **g.....t..g..a...........~~...t.......tga..catagact~~...........t......t....**

**KF667322.1**  **g.....t..g..a...........~~...t.......tga..catagact~~...........t......t....**

**KF667323.1**  **g.....t..g..a...........~~...t.......tga..catagact~~..g...............t....**

**KF667324.1**  **g.....t..g..a...........~~...t.......tga..catagact~~..g...............t....**

**KF667325.1**  **g.....t..g..a...........~~...t.....a.tga..catagact~~...........t......t....**

**KF667326.1**  **g.....t..g..a...........~~...t.......tga..catagact~~..g...............t....**

**KF667327.1**  **g.....t..g..a...........~~...t.......tga..catagact~~..g...............t....**

**KF711994.1**  **........................~~...........tga..catagact~~.......................**

**KF907505.1**  **........................~~...........tga..catagact~~.......................**

**KM658163.1**  **g.....t..g..a...........~~...t.......tga..catagact~~...........t......t....**

**KM677246.1**  **g..a.....g..a.....g.....~~t..t.......t.a.gcatagact~~c.c..t.....t......t.g..**

**KP164498.2**  **........................~~...........tga..catagact~~.......................**

**KR265316.1**  **......t.................~~...........tga..catagact~~..............t........**

**KT229572.1**  **g.....t..g..a...........~~...t.......tga..catagact~~..g..............tt....**

**KT229573.1**  **g.....t..g..a...........~~...t.......tga..catagact~~..g..............tt....**

**KT229574.1**  **g.....t..g..a...........~~...t.......tga..catagact~~..g..............tt....**

**KT229575.1**  **g.....t..g..a...........~~...t.......tga..catagact~~..g..............tt....**

**KT239164.1**  **........................~~t..........tga..cacagact~~..................t....**

**KT957419.1**  **g.....t..g..a...........~~...t.......tga..catagact~~..g..............tt....**

**KT957420.1**  **g.....t..g..a...........~~...t.......tga..catagact~~..g..............tt....**

**KT957421.1**  **g.....t..g..a...........~~...t.......tga.tcatagact~~..g..............tt....**

**KT957422.1**  **g.....t..g..a...........~~t..t..t....tga..catagact~~.................tt....**

**KT957423.1**  **g.....t..g..a...........~~t..t..t....tga..catagact~~.................tt....**

**KU323483.1**  **........................~~...........tga..catagact~~.......................**

**KU351667.1**  **g.....t..g..a...........~~t..t.......tga..catagact~~..g..............tt....**

**KU351668.1**  **g.....t..g..a...........~~...t.......tga..catagact~~...........t......t....**

**KU363309.1**  **......t.................~~...........tga..catagact~~..............t........**

**KU508408.1**  **g.....t..g..a...........~~...t.......tga..catagact~~...........t......t....**

**KU508409.1**  **g.....t..g..a...........~~...t.......tga..catagact~~...........t......t....**

**KU821122.1**  **........................~~...........tga..catagact~~..g....................**

**KU871316.1**  **........................~~...........tga..catagact~~..g....................**

**KU871317.1**  **........................~~...........tga..catagact~~..g....................**

**KU871318.1**  **........................~~...........tga..catagact~~..g....................**

**KU871319.1**  **........................~~...........tga..catagact~~..g....................**

**KU871320.1**  **........................~~...........tga..catagact~~..g....................**

**KU871321.1**  **........................~~...........tga..catagact~~..g....................**

**KU871322.1**  **........................~~...........tga..catagact~~..g....................**

**KU871323.1**  **........................~~...........tga..catagact~~..g....................**

**KU871324.1**  **........................~~...........tga..catagact~~.......................**

**KU871325.1**  **........................~~...........tga..catagact~~.......................**

**KU871326.1**  **........................~~...........tga..catagact~~..g....................**

**KU871327.1**  **........................~~...........tga..catagact~~.......................**

**KU871328.1**  **........................~~...........tga..catagact~~.......................**

**KU871329.1**  **........................~~...........tga..cata.act~~.......................**

**KU871330.1**  **........................~~...........tga..catagact~~..g....................**

**KU871331.1**  **........................~~...........tga..catagact~~..g....................**

**KU871332.1**  **........................~~...........tga..catagact~~.......................**

**KU871333.1**  **........................~~...........tga..catagact~~..g....................**

**KU871334.1**  **........................~~...........tga..catagact~~..g....................**

**KU871335.1**  **........................~~...........tga..catagact~~..g....................**

**KU871336.1**  **........................~~...........tga..catagact~~..g....................**

**KU871337.1**  **........................~~...........tga..catagact~~..g....................**

**KU871338.1**  **........................~~...........tga..catagact~~..g....................**

**KU871339.1**  **........................~~...........tga..catagact~~..g....................**

**KU871340.1**  **........................~~...........tga..catagact~~..g....................**

**KU871341.1**  **........................~~...........tga..catagact~~..g....................**

**KU871342.1**  **........................~~...........tga..catagact~~..g....................**

**KU871343.1**  **........................~~...........tga..catagact~~..g....................**

**KU871344.1**  **........................~~...........tga..catagact~~.......................**

**KU871345.1**  **........................~~...........tga..catagact~~..g....................**

**KU871346.1**  **........................~~...........tga..catagact~~.......................**

**KU871347.1**  **........................~~...........tga..catagact~~..g....................**

**KU871348.1**  **........................~~...........tga..catagact~~..g....................**

**KU871349.1**  **........................~~...........tga..catagact~~.......................**

**KU871350.1**  **........................~~...........tga..catagact~~.......................**

**KU871351.1**  **........................~~...........tga..catagact~~.......................**

**KU871352.1**  **........................~~...........tga..catagact~~..g....................**

**KU871353.1**  **........................~~...........tga..catagact~~..g....................**

**KU871354.1**  **........................~~...........tga..catagact~~..g....................**

**KU871355.1**  **........................~~...........tga..catagact~~..g....................**

**KU871356.1**  **........................~~..........ttga..catagact~~.......................**

**KU871357.1**  **........................~~...........tga..catagact~~..g....................**

**KU871358.1**  **........................~~...........tga..catagact~~.......................**

**KU871359.1**  **........................~~...........tga..catagact~~.......................**

**KU871360.1**  **........................~~...........tga..catagact~~.......................**

**KU871361.1**  **........................~~...........tga..catagact~~.......................**

**KU871362.1**  **........................~~...........tga..catagact~~.......................**

**KU871363.1**  **........................~~...........tga..catagact~~.......................**

**KU871364.1**  **........................~~...........tga..catagact~~..g....................**

**KU871365.1**  **........................~~...........tga..catagact~~.......................**

**KU871366.1**  **........................~~...........tga..catagact~~.......................**

**KU871367.1**  **........................~~...........tga..catagact~~.......................**

**KU871368.1**  **........................~~...........tga..catagact~~.......................**

**KU871369.1**  **........................~~...........tga..catagact~~.......................**

**KU871370.1**  **........................~~...........tga..catagact~~.......................**

**KU871371.1**  **........................~~...........tga..catagact~~..g....................**

**KX357114.1**  **g.....t..g..a...........~~...t.......tga..catagact~~...........t.....tt....**

**KX779520.1**  **........................~~...........tga..catagact~~.......................**

**KX779521.1**  **........................~~...........tga..catagact~~.......................**

**KX779522.1**  **........................~~...........tga..catagact~~.......................**

**L48961.1**  **........................~~...........tga..cacagact~~..............t........**

**L78128.1**  **........................~~...........tga..cacagact~~.......................**

**M18370.1**  **........................~~...........tga..catagact~~.......................**

**M55506.1**  **........................~~...........tga..catagact~~.......................**

**NC_001437.1**  **........................~~...........tga..catagact~~.......................**

**JN644310.1**  **........................~~...t.......tga..catagact~~.......................**

**U14163.1**  **........................~~...........tga..catagact~~.......................**

**U15763.1**  **........................~~...........tga..catagact~~.......................**

**U47032.1**  **......t.................~~...........tga..catagact~~..............t........**

**7) NS2A v1 assay**

Accession 3563 3675

Numbers: ....|....|....|....|....|....|....|....|....|....|....|....|

**AGCTGGGCCTTCTGGT~~CTTCGCAAGAGGTGGACGGCCA~~CTTGTGCTGATGCTTGGG**

**EF571853.1**  **................~~.....t................~~..................**

**AB051292.1**  **................~~......................~~..................**

**AB196923.1**  **................~~......................~~..................**

**AB196924.1**  **................~~......................~~..................**

**AB196925.1**  **................~~......................~~..................**

**AB196926.1**  **................~~......................~~..................**

**AB241118.1**  **................~~......................~~..................**

**AB241119.1**  **................~~......................~~..................**

**AB269326.1**  **................~~......................~~..................**

**AB471666.2**  **................~~......................~~..................**

**AB471667.2**  **................~~......................~~..................**

**AB471668.2**  **................~~......................~~..................**

**AB471669.2**  **................~~......................~~..................**

**AB471670.2**  **................~~......................~~..................**

**AB551990.1**  **................~~......................~~..................**

**AB551991.1**  **................~~......................~~..................**

**AB551992.1**  **................~~......................~~..................**

**AB569980.2**  **................~~......................~~..................**

**AB569981.2**  **................~~......................~~..................**

**AB569982.2**  **................~~......................~~..................**

**AB569983.2**  **................~~......................~~..................**

**AB569984.2**  **................~~......................~~..................**

**AB569985.2**  **................~~......................~~..................**

**AB569987.2**  **................~~......................~~..................**

**AB569988.2**  **................~~......................~~..................**

**AB569989.2**  **................~~......................~~..................**

**AB569990.2**  **................~~......................~~..................**

**AB594829.1**  **................~~......................~~..................**

**AB698905.1**  **................~~......................~~..................**

**AB698906.1**  **................~~......................~~..................**

**AB698907.1**  **................~~......................~~..................**

**AB698908.1**  **................~~......................~~..................**

**AB698909.1**  **................~~......................~~..................**

**AB830335.1**  **................~~......................~~..................**

**AB853904.1**  **................~~......................~~..................**

**AB920399.1**  **................~~......................~~..................**

**AB981183.1**  **................~~......................~~..................**

**AB981184.1**  **................~~......................~~..................**

**AF014160.1**  **................~~......................~~..................**

**AF014161.1**  **................~~......................~~..................**

**AF045551.2**  **................~~......................~~..................**

**AF069076.1**  **................~~......................~~..................**

**AF075723.1**  **................~~......................~~..................**

**AF080251.1**  **................~~......................~~..................**

**AF098735.1**  **................~~......................~~..................**

**AF098736.1**  **..a.............~~...g..................~~..................**

**AF098737.1**  **................~~......................~~..................**

**AF217620.1**  **................~~......................~~..................**

**AF221499.1**  **................~~......................~~..................**

**AF221500.1**  **................~~......................~~..................**

**AF254452.1**  **................~~......................~~..................**

**AF254453.1**  **................~~......................~~..................**

**AF315119.1**  **................~~......................~~..................**

**AF416457.1**  **................~~......................~~..................**

**AF486638.1**  **................~~......................~~..................**

**AY184212.1**  **..t.............~~......................~~..................**

**AY303791.1**  **................~~......................~~..................**

**AY303792.1**  **................~~......................~~..................**

**AY303793.1**  **................~~......................~~..................**

**AY303794.1**  **................~~......................~~..................**

**AY303795.1**  **................~~......................~~..................**

**AY303796.1**  **................~~......................~~..................**

**AY303797.1**  **................~~......................~~..................**

**AY303798.1**  **................~~......................~~..................**

**AY316157.1**  **................~~......................~~..................**

**AY508812.1**  **................~~......................~~..................**

**AY508813.1**  **................~~......................~~..................**

**AY585242.1**  **................~~......................~~..................**

**AY585243.1**  **................~~......................~~..................**

**AY849939.1**  **................~~t.....................~~..................**

**D90194.1**  **................~~......................~~..................**

**D90195.1**  **................~~......................~~..................**

**DD298237.1**  **................~~......................~~..................**

**DD298243.1**  **................~~......................~~..................**

**DD367827.1**  **................~~......................~~..................**

**DD367828.1**  **................~~......................~~..................**

**DI075091.1**  **................~~......................~~..................**

**DI085220.1**  **................~~......................~~..................**

**EF107523.1**  **................~~......................~~............t.....**

**EF543861.1**  **................~~......................~~..................**

**EF623987.1**  **................~~......................~~..................**

**EF623988.1**  **................~~......................~~..................**

**EF623989.1**  **................~~......................~~..................**

**EU429297.1**  **................~~......................~~..................**

**EU693899.1**  **................~~......................~~..................**

**EU880214.1**  **................~~......................~~..................**

**FJ185036.1**  **................~~......................~~..................**

**FJ185037.1**  **................~~......................~~..................**

**FJ495189.1**  **................~~......................~~..................**

**GQ199609.1**  **................~~......................~~..................**

**GQ902058.1**  **..t.............~~......................~~..................**

**GQ902059.1**  **..t.............~~......................~~..................**

**GQ902060.1**  **................~~......................~~..................**

**GQ902061.1**  **................~~......................~~..................**

**GQ902062.1**  **................~~......................~~..................**

**GQ902063.1**  **................~~......................~~..................**

**GQ918133.2**  **................~~......................~~..................**

**GU187972.1**  **................~~......................~~..................**

**GU205163.1**  **................~~......................~~..................**

**GU556217.1**  **................~~......................~~..................**

**HE861351.1**  **................~~.....t................~~..................**

**HM228921.1**  **................~~......................~~..................**

**HM366552.1**  **................~~......................~~..................**

**HM596272.1**  **..t.............~~......................~~.....t............**

**HQ223285.1**  **................~~......................~~..................**

**HQ223286.1**  **................~~......................~~..................**

**HQ223287.1**  **................~~......................~~..................**

**HQ652538.1**  **................~~......................~~..................**

**HQ893545.1**  **................~~......................~~..................**

**JF499788.1**  **................~~......................~~..................**

**JF499789.1**  **................~~......................~~..................**

**JF499790.1**  **................~~......................~~..................**

**JF706267.1**  **................~~......................~~..................**

**JF706268.1**  **................~~......................~~..................**

**JF706269.1**  **................~~......................~~..................**

**JF706270.1**  **................~~......................~~..................**

**JF706271.1**  **................~~......................~~..................**

**JF706272.1**  **................~~......................~~..................**

**JF706273.1**  **................~~......................~~..................**

**JF706274.1**  **..t.............~~......................~~..................**

**JF706275.1**  **................~~......................~~..................**

**JF706276.1**  **................~~......................~~..................**

**JF706277.1**  **................~~......................~~..................**

**JF706278.1**  **................~~......................~~..................**

**JF706279.1**  **................~~......................~~..................**

**JF706280.1**  **................~~......................~~..................**

**JF706281.1**  **................~~......................~~..................**

**JF706282.1**  **................~~......................~~..................**

**JF706283.1**  **................~~......................~~..................**

**JF706284.1**  **................~~......................~~..................**

**JF706285.1**  **................~~......................~~..................**

**JF706286.1**  **................~~......................~~..................**

**JF915894.1**  **..t.............~~......................~~.....t............**

**JN381830.1**  **................~~......................~~..................**

**JN381831.1**  **................~~......................~~..................**

**JN381832.1**  **................~~......................~~..................**

**JN381833.1**  **................~~......................~~..................**

**JN381834.1**  **................~~......................~~..................**

**JN381835.1**  **................~~......................~~..................**

**JN381836.1**  **................~~......................~~..................**

**JN381837.1**  **................~~......................~~..................**

**JN381838.1**  **................~~......................~~..................**

**JN381839.1**  **................~~......................~~..................**

**JN381840.1**  **..t.............~~......................~~..................**

**JN381841.1**  **................~~......................~~..................**

**JN381842.1**  **................~~......................~~..................**

**JN381843.1**  **................~~......................~~..................**

**JN381844.1**  **................~~......................~~..................**

**JN381845.1**  **................~~......................~~..................**

**JN381846.1**  **................~~......................~~..................**

**JN381847.1**  **................~~......................~~..................**

**JN381848.1**  **................~~......................~~..................**

**JN381849.1**  **................~~......................~~..................**

**JN381850.1**  **................~~......................~~..................**

**JN381851.1**  **................~~......................~~..................**

**JN381852.1**  **................~~......................~~..................**

**JN381853.1**  **................~~......................~~..................**

**JN381854.1**  **................~~......................~~..................**

**JN381855.1**  **................~~......................~~..................**

**JN381856.1**  **................~~......................~~..................**

**JN381857.1**  **................~~......................~~..................**

**JN381858.1**  **................~~......................~~..................**

**JN381859.1**  **................~~......................~~..................**

**JN381860.1**  **................~~......................~~..................**

**JN381861.1**  **................~~......................~~..................**

**JN381862.1**  **................~~......................~~..................**

**JN381863.1**  **................~~......................~~..................**

**JN381864.1**  **................~~......................~~..................**

**JN381865.1**  **................~~......................~~..................**

**JN381866.1**  **................~~......................~~..................**

**JN381867.1**  **................~~......................~~..................**

**JN381868.1**  **................~~......................~~..................**

**JN381869.1**  **................~~......................~~..................**

**JN381870.1**  **................~~......................~~..................**

**JN381871.1**  **................~~......................~~..................**

**JN381872.1**  **................~~......................~~..................**

**JN381873.1**  **................~~......................~~..................**

**JN604986.1**  **................~~......................~~..................**

**JN711458.1**  **................~~......................~~..................**

**JN711459.1**  **................~~......................~~..................**

**JN864064.1**  **................~~......................~~..................**

**JQ031753.1**  **................~~......................~~..................**

**JQ086762.1**  **................~~......................~~..................**

**JQ086763.1**  **................~~......................~~..................**

**JX050179.1**  **................~~......................~~..................**

**JX072965.1**  **................~~......................~~..................**

**JX131374.1**  **.......t........~~......................~~..................**

**KC183732.1**  **................~~......................~~..................**

**KC196115.1**  **................~~......................~~..................**

**KC517497.1**  **................~~......................~~..................**

**KC915016.1**  **................~~......................~~..................**

**KF297915.1**  **................~~......................~~..................**

**KF297916.1**  **................~~......................~~..................**

**KF667310.1**  **................~~......................~~..................**

**KF667311.1**  **................~~......................~~..................**

**KF667312.1**  **................~~......................~~..................**

**KF667313.1**  **................~~......................~~..................**

**KF667314.1**  **................~~......................~~..................**

**KF667315.1**  **................~~......................~~..................**

**KF667316.1**  **................~~......................~~..................**

**KF667317.1**  **................~~......................~~..................**

**KF667318.1**  **................~~......................~~............t.....**

**KF667319.1**  **................~~......................~~..................**

**KF667320.1**  **................~~......................~~..................**

**KF667321.1**  **................~~......................~~..................**

**KF667322.1**  **................~~......................~~..................**

**KF667323.1**  **................~~......................~~..................**

**KF667324.1**  **................~~......................~~..................**

**KF667325.1**  **................~~......................~~..................**

**KF667326.1**  **................~~......................~~..................**

**KF667327.1**  **................~~......................~~..................**

**KF711994.1**  **................~~......................~~..................**

**KF907505.1**  **................~~......................~~..................**

**KM658163.1**  **................~~......................~~..................**

**KM677246.1**  **..t.............~~......................~~.....t............**

**KP164498.2**  **................~~......................~~..................**

**KR265316.1**  **................~~t.....................~~..................**

**KT229572.1**  **................~~......................~~..................**

**KT229573.1**  **................~~......................~~..................**

**KT229574.1**  **................~~......................~~..................**

**KT229575.1**  **................~~......................~~..................**

**KT239164.1**  **.......t........~~......................~~..................**

**KT957419.1**  **................~~......................~~..................**

**KT957420.1**  **................~~......................~~..................**

**KT957421.1**  **................~~......................~~.....a............**

**KT957422.1**  **................~~......................~~..................**

**KT957423.1**  **................~~......................~~..................**

**KU323483.1**  **................~~......................~~..................**

**KU351667.1**  **................~~......................~~..................**

**KU351668.1**  **................~~......................~~..................**

**KU363309.1**  **................~~......................~~..................**

**KU508408.1**  **................~~......................~~..................**

**KU508409.1**  **................~~......................~~..................**

**KU821122.1**  **................~~......................~~..................**

**KU871316.1**  **................~~......................~~..................**

**KU871317.1**  **................~~......................~~..................**

**KU871318.1**  **................~~......................~~..................**

**KU871319.1**  **................~~......................~~..................**

**KU871320.1**  **................~~......................~~..................**

**KU871321.1**  **................~~......................~~..................**

**KU871322.1**  **................~~......................~~..................**

**KU871323.1**  **................~~......................~~..................**

**KU871324.1**  **................~~......................~~..................**

**KU871325.1**  **................~~......................~~..................**

**KU871326.1**  **................~~......................~~..................**

**KU871327.1**  **................~~......................~~..................**

**KU871328.1**  **................~~......................~~..................**

**KU871329.1**  **................~~......................~~..................**

**KU871330.1**  **................~~......................~~..................**

**KU871331.1**  **................~~......................~~..................**

**KU871332.1**  **................~~....................t.~~..................**

**KU871333.1**  **................~~......................~~..................**

**KU871334.1**  **................~~......................~~..................**

**KU871335.1**  **................~~......................~~..................**

**KU871336.1**  **................~~......................~~..................**

**KU871337.1**  **................~~......................~~..................**

**KU871338.1**  **................~~......................~~..................**

**KU871339.1**  **................~~......................~~..................**

**KU871340.1**  **................~~......................~~..................**

**KU871341.1**  **................~~......................~~..................**

**KU871342.1**  **................~~......................~~..................**

**KU871343.1**  **................~~......................~~..................**

**KU871344.1**  **................~~......................~~..................**

**KU871345.1**  **................~~......................~~..................**

**KU871346.1**  **................~~......................~~..................**

**KU871347.1**  **................~~......................~~..................**

**KU871348.1**  **................~~......................~~..................**

**KU871349.1**  **................~~......................~~..................**

**KU871350.1**  **................~~......................~~..................**

**KU871351.1**  **................~~......................~~..................**

**KU871352.1**  **................~~......................~~..................**

**KU871353.1**  **................~~......................~~..................**

**KU871354.1**  **................~~......................~~..................**

**KU871355.1**  **................~~......................~~..................**

**KU871356.1**  **................~~......................~~..................**

**KU871357.1**  **................~~......................~~..................**

**KU871358.1**  **................~~......................~~..................**

**KU871359.1**  **................~~....................t.~~..................**

**KU871360.1**  **................~~......................~~..................**

**KU871361.1**  **................~~......................~~..................**

**KU871362.1**  **................~~....................y.~~..................**

**KU871363.1**  **................~~....................t.~~..................**

**KU871364.1**  **................~~......................~~..................**

**KU871365.1**  **................~~......................~~..................**

**KU871366.1**  **................~~......................~~..................**

**KU871367.1**  **................~~......................~~..................**

**KU871368.1**  **................~~......................~~..................**

**KU871369.1**  **................~~......................~~..................**

**KU871370.1**  **................~~......................~~..................**

**KU871371.1**  **................~~......................~~..................**

**KX357114.1**  **................~~......................~~..................**

**KX779520.1**  **................~~......................~~..................**

**KX779521.1**  **................~~......................~~..................**

**KX779522.1**  **................~~......................~~..................**

**L48961.1**  **................~~......................~~..................**

**L78128.1**  **................~~......................~~..................**

**M18370.1**  **................~~......................~~..................**

**M55506.1**  **................~~......................~~..................**

**NC_001437.1**  **................~~......................~~..................**

**JN644310.1**  **................~~......................~~..................**

**U14163.1**  **................~~......................~~..................**

**U15763.1**  **................~~......................~~..................**

**U47032.1**  **................~~......................~~..................**

**8) NS5 v2 assay**

Accession 9925 10240

Numbers: ....|....|....|....|....|....|....|....|....|....|....|..

**GDGCTGGATGGAATGTGA~~AGGAGAGTGGATGACYAC~~GAGGACATCTGGTGTGG**

**EF571853.1**  **..................~~..................~~.................**

**AB051292.1**  **..................~~..................~~.................**

**AB196923.1**  **..................~~..................~~.................**

**AB196924.1**  **..................~~..................~~.................**

**AB196925.1**  **..................~~..................~~.................**

**AB196926.1**  **..................~~..................~~.................**

**AB241118.1**  **..................~~..................~~.................**

**AB241119.1**  **..................~~..................~~.................**

**AB269326.1**  **..................~~..................~~.................**

**AB471666.2**  **..................~~..................~~.................**

**AB471667.2**  **..................~~..................~~.................**

**AB471668.2**  **..................~~..................~~.................**

**AB471669.2**  **..................~~..................~~.................**

**AB471670.2**  **..................~~..................~~.................**

**AB551990.1**  **..................~~..................~~.................**

**AB551991.1**  **..................~~..................~~.................**

**AB551992.1**  **..................~~..................~~.................**

**AB569980.2**  **..................~~..................~~.................**

**AB569981.2**  **..................~~..................~~.................**

**AB569982.2**  **..................~~..................~~.................**

**AB569983.2**  **..................~~..................~~.................**

**AB569984.2**  **..................~~..................~~.................**

**AB569985.2**  **..................~~..................~~.................**

**AB569987.2**  **..................~~..................~~.................**

**AB569988.2**  **..................~~..................~~.................**

**AB569989.2**  **..................~~..................~~.................**

**AB569990.2**  **..................~~..................~~.................**

**AB594829.1**  **..................~~..................~~.................**

**AB698905.1**  **..................~~..................~~.................**

**AB698906.1**  **..................~~..................~~.................**

**AB698907.1**  **..................~~..................~~.................**

**AB698908.1**  **..................~~..................~~.................**

**AB698909.1**  **..................~~..................~~.................**

**AB830335.1**  **..................~~..................~~.................**

**AB853904.1**  **..................~~..................~~.................**

**AB920399.1**  **..................~~..................~~.................**

**AB981183.1**  **..................~~..................~~.................**

**AB981184.1**  **..................~~..................~~.................**

**AF014160.1**  **..................~~..................~~.................**

**AF014161.1**  **..................~~..................~~.................**

**AF045551.2**  **..................~~..................~~.................**

**AF069076.1**  **..................~~..................~~.................**

**AF075723.1**  **..................~~..................~~.................**

**AF080251.1**  **..................~~..................~~.................**

**AF098735.1**  **..................~~..................~~.................**

**AF098736.1**  **..................~~...n..............~~.................**

**AF098737.1**  **..................~~............cc....~~.................**

**AF217620.1**  **................a.~~g.................~~..a...........c..**

**AF221499.1**  **....c.............~~..................~~.................**

**AF221500.1**  **....c.............~~..................~~.................**

**AF254452.1**  **..........c.......~~..................~~.................**

**AF254453.1**  **..........t.......~~..................~~.................**

**AF315119.1**  **..................~~..................~~.................**

**AF416457.1**  **..................~~..................~~.................**

**AF486638.1**  **..................~~..................~~.................**

**AY184212.1**  **.......g..........~~g.....a........g..~~........t.....c..**

**AY303791.1**  **..................~~..................~~.................**

**AY303792.1**  **..................~~..................~~.................**

**AY303793.1**  **....c.............~~...g..............~~.................**

**AY303794.1**  **....c.............~~...g..............~~.................**

**AY303795.1**  **..................~~..................~~.................**

**AY303796.1**  **..................~~..................~~.................**

**AY303797.1**  **..................~~..................~~.................**

**AY303798.1**  **..................~~..................~~.................**

**AY316157.1**  **..................~~..................~~.................**

**AY508812.1**  **..................~~..................~~.................**

**AY508813.1**  **..................~~..................~~.................**

**AY585242.1**  **..................~~..................~~.................**

**AY585243.1**  **..................~~..................~~.................**

**AY849939.1**  **..................~~..................~~.................**

**D90194.1**  **..................~~..................~~.................**

**D90195.1**  **..................~~..................~~.................**

**DD298237.1**  **..................~~..................~~.................**

**DD298243.1**  **..................~~..................~~.................**

**DD367827.1**  **..................~~..................~~.................**

**DD367828.1**  **..................~~..................~~.................**

**DI075091.1**  **..................~~..................~~.................**

**DI085220.1**  **..................~~..................~~.................**

**EF107523.1**  **..................~~..................~~.................**

**EF543861.1**  **..................~~..................~~.................**

**EF623987.1**  **..................~~..................~~.................**

**EF623988.1**  **..................~~..................~~.................**

**EF623989.1**  **..................~~..................~~.................**

**EU429297.1**  **..................~~..................~~.................**

**EU693899.1**  **..................~~..................~~.................**

**EU880214.1**  **..................~~..................~~.................**

**FJ185036.1**  **..................~~..................~~.................**

**FJ185037.1**  **..................~~..................~~.................**

**FJ495189.1**  **..................~~..................~~.................**

**GQ199609.1**  **..................~~..................~~.................**

**GQ902058.1**  **..................~~..................~~.................**

**GQ902059.1**  **..................~~..................~~.................**

**GQ902060.1**  **..................~~..................~~.................**

**GQ902061.1**  **..................~~..................~~.................**

**GQ902062.1**  **..................~~..................~~.................**

**GQ902063.1**  **..................~~..................~~.................**

**GQ918133.2**  **..................~~..................~~.................**

**GU187972.1**  **..................~~..................~~.................**

**GU205163.1**  **..................~~..................~~.................**

**GU556217.1**  **..................~~..................~~.................**

**HE861351.1**  **..................~~..................~~.................**

**HM228921.1**  **..................~~..................~~.................**

**HM366552.1**  **..................~~..................~~.................**

**HM596272.1**  **....a........c....~~......a........a..~~..a..............**

**HQ223285.1**  **................a.~~..................~~.....t...........**

**HQ223286.1**  **................a.~~..................~~..a...........c..**

**HQ223287.1**  **................a.~~..................~~..a..............**

**HQ652538.1**  **..................~~g.................~~.................**

**HQ893545.1**  **..................~~..................~~.................**

**JF499788.1**  **..................~~..................~~.................**

**JF499789.1**  **..................~~..................~~.................**

**JF499790.1**  **..................~~..................~~.................**

**JF706267.1**  **..................~~..................~~.................**

**JF706268.1**  **..................~~..................~~.................**

**JF706269.1**  **..................~~..................~~.................**

**JF706270.1**  **..................~~..................~~.................**

**JF706271.1**  **..................~~..................~~.................**

**JF706272.1**  **..................~~..................~~.................**

**JF706273.1**  **..................~~..................~~.................**

**JF706274.1**  **..................~~..................~~.................**

**JF706275.1**  **..................~~..................~~.................**

**JF706276.1**  **..................~~..................~~.................**

**JF706277.1**  **..................~~..................~~.................**

**JF706278.1**  **..................~~..................~~.................**

**JF706279.1**  **..................~~..................~~.................**

**JF706280.1**  **..................~~..................~~.................**

**JF706281.1**  **..................~~..................~~.................**

**JF706282.1**  **..................~~..................~~.................**

**JF706283.1**  **..................~~..................~~.................**

**JF706284.1**  **..................~~..................~~.................**

**JF706285.1**  **..................~~..................~~.................**

**JF706286.1**  **..................~~..................~~.................**

**JF915894.1**  **....a...........a.~~......a........a..~~..a...........c..**

**JN381830.1**  **..................~~..................~~.................**

**JN381831.1**  **..................~~..................~~.................**

**JN381832.1**  **..................~~..................~~.................**

**JN381833.1**  **..................~~..................~~.................**

**JN381834.1**  **..................~~..................~~.................**

**JN381835.1**  **..................~~..................~~.................**

**JN381836.1**  **..................~~..................~~.................**

**JN381837.1**  **..................~~..................~~.................**

**JN381838.1**  **..................~~..................~~.................**

**JN381839.1**  **..................~~..................~~.................**

**JN381840.1**  **..................~~..................~~.................**

**JN381841.1**  **..................~~..................~~.................**

**JN381842.1**  **..................~~..................~~.................**

**JN381843.1**  **..................~~..................~~.................**

**JN381844.1**  **..................~~..................~~.................**

**JN381845.1**  **..................~~..................~~.................**

**JN381846.1**  **..................~~..................~~.................**

**JN381847.1**  **..................~~..................~~.................**

**JN381848.1**  **..................~~..................~~.................**

**JN381849.1**  **..................~~..................~~.................**

**JN381850.1**  **..................~~..................~~.................**

**JN381851.1**  **..................~~..................~~.................**

**JN381852.1**  **..................~~..................~~.................**

**JN381853.1**  **..................~~..................~~.................**

**JN381854.1**  **..................~~..................~~.................**

**JN381855.1**  **..................~~..................~~.................**

**JN381856.1**  **..................~~..................~~.................**

**JN381857.1**  **..................~~..................~~.................**

**JN381858.1**  **..................~~..................~~.................**

**JN381859.1**  **..................~~..................~~.................**

**JN381860.1**  **..................~~..................~~.................**

**JN381861.1**  **..................~~..................~~.................**

**JN381862.1**  **..................~~..................~~.................**

**JN381863.1**  **..................~~..................~~.................**

**JN381864.1**  **..................~~..................~~.................**

**JN381865.1**  **..................~~..................~~.................**

**JN381866.1**  **..................~~..................~~.................**

**JN381867.1**  **..................~~..................~~.................**

**JN381868.1**  **..................~~..................~~.................**

**JN381869.1**  **..................~~..................~~.................**

**JN381870.1**  **..................~~..................~~.................**

**JN381871.1**  **..................~~..................~~.................**

**JN381872.1**  **..................~~..................~~.................**

**JN381873.1**  **....c.............~~..................~~.................**

**JN604986.1**  **..................~~..................~~.................**

**JN711458.1**  **..................~~..................~~.................**

**JN711459.1**  **..................~~..................~~.................**

**JN864064.1**  **..................~~..................~~.................**

**JQ031753.1**  **..................~~..................~~.................**

**JQ086762.1**  **..................~~..................~~.................**

**JQ086763.1**  **..................~~..................~~.................**

**JX050179.1**  **..................~~..................~~.................**

**JX072965.1**  **..................~~..................~~.................**

**JX131374.1**  **..................~~..................~~.................**

**KC183732.1**  **..................~~..................~~.................**

**KC196115.1**  **..................~~..................~~.................**

**KC517497.1**  **..................~~..................~~.................**

**KC915016.1**  **..................~~..................~~.................**

**KF297915.1**  **..................~~..................~~.................**

**KF297916.1**  **..................~~..................~~.................**

**KF667310.1**  **....c.............~~...g..............~~..............c..**

**KF667311.1**  **....c.............~~...g..............~~.................**

**KF667312.1**  **..................~~..................~~.................**

**KF667313.1**  **..................~~..................~~.................**

**KF667314.1**  **....c.............~~...g..............~~.................**

**KF667315.1**  **....c.............~~...g..............~~.................**

**KF667316.1**  **..................~~..................~~.................**

**KF667317.1**  **..................~~..................~~.................**

**KF667318.1**  **..................~~..................~~.................**

**KF667319.1**  **..................~~..................~~.................**

**KF667320.1**  **..................~~..................~~.................**

**KF667321.1**  **..................~~..................~~.................**

**KF667322.1**  **..................~~..................~~.................**

**KF667323.1**  **..................~~..................~~.................**

**KF667324.1**  **..................~~..................~~.................**

**KF667325.1**  **..................~~..................~~.................**

**KF667326.1**  **..................~~..................~~.................**

**KF667327.1**  **..................~~..................~~.................**

**KF711994.1**  **..................~~..................~~.................**

**KF907505.1**  **..................~~..................~~.................**

**KM658163.1**  **..................~~..................~~.................**

**KM677246.1**  **....a........c....~~......a........a..~~..a..............**

**KP164498.2**  **..................~~..................~~.................**

**KR265316.1**  **..................~~..................~~.................**

**KT229572.1**  **..................~~..................~~.................**

**KT229573.1**  **..................~~..................~~.................**

**KT229574.1**  **..................~~..................~~.................**

**KT229575.1**  **..................~~..................~~.................**

**KT239164.1**  **..................~~..................~~.................**

**KT957419.1**  **..................~~..................~~.................**

**KT957420.1**  **..................~~..................~~.................**

**KT957421.1**  **..................~~..................~~.................**

**KT957422.1**  **..................~~..................~~.................**

**KT957423.1**  **..................~~..................~~.................**

**KU323483.1**  **..................~~..................~~.................**

**KU351667.1**  **..................~~..................~~.................**

**KU351668.1**  **..................~~..................~~.................**

**KU363309.1**  **..................~~..................~~.................**

**KU508408.1**  **..................~~..................~~.................**

**KU508409.1**  **..................~~..................~~.................**

**KU821122.1**  **..................~~..................~~.................**

**KU871316.1**  **..................~~..................~~.................**

**KU871317.1**  **..................~~..................~~.................**

**KU871318.1**  **..................~~..................~~.................**

**KU871319.1**  **..................~~..................~~.................**

**KU871320.1**  **..................~~..................~~.................**

**KU871321.1**  **..................~~..................~~.................**

**KU871322.1**  **..................~~..................~~.................**

**KU871323.1**  **..................~~..................~~.................**

**KU871324.1**  **..................~~..................~~.................**

**KU871325.1**  **..................~~..................~~.................**

**KU871326.1**  **..................~~..................~~.................**

**KU871327.1**  **..................~~..................~~.................**

**KU871328.1**  **..................~~..................~~.................**

**KU871329.1**  **..................~~..................~~.................**

**KU871330.1**  **..................~~..................~~.................**

**KU871331.1**  **..................~~..................~~.................**

**KU871332.1**  **..................~~..................~~.................**

**KU871333.1**  **..................~~..................~~.................**

**KU871334.1**  **..................~~..................~~.................**

**KU871335.1**  **..................~~..................~~.................**

**KU871336.1**  **..................~~..................~~.................**

**KU871337.1**  **..................~~..................~~.................**

**KU871338.1**  **..................~~..................~~.................**

**KU871339.1**  **..................~~..................~~.................**

**KU871340.1**  **..................~~..................~~.................**

**KU871341.1**  **..................~~..................~~.................**

**KU871342.1**  **..................~~..................~~.................**

**KU871343.1**  **..................~~..................~~.................**

**KU871344.1**  **..................~~..................~~.................**

**KU871345.1**  **..................~~..................~~.................**

**KU871346.1**  **..................~~..................~~.................**

**KU871347.1**  **..................~~..................~~.................**

**KU871348.1**  **..................~~..................~~.................**

**KU871349.1**  **..................~~..................~~.................**

**KU871350.1**  **..................~~..................~~.................**

**KU871351.1**  **..................~~..................~~.................**

**KU871352.1**  **..................~~..................~~.................**

**KU871353.1**  **..................~~..................~~.................**

**KU871354.1**  **..................~~..................~~.................**

**KU871355.1**  **..................~~..................~~.................**

**KU871356.1**  **..................~~..................~~.................**

**KU871357.1**  **..................~~..................~~.................**

**KU871358.1**  **..................~~..................~~.................**

**KU871359.1**  **..................~~..................~~.................**

**KU871360.1**  **..................~~..................~~.................**

**KU871361.1**  **..................~~..................~~.................**

**KU871362.1**  **..................~~..................~~.................**

**KU871363.1**  **..................~~..................~~.................**

**KU871364.1**  **..................~~..................~~.................**

**KU871365.1**  **..................~~..................~~.................**

**KU871366.1**  **..................~~..................~~.................**

**KU871367.1**  **..................~~..................~~.................**

**KU871368.1**  **..................~~..................~~.................**

**KU871369.1**  **..................~~..................~~.................**

**KU871370.1**  **..................~~..................~~.................**

**KU871371.1**  **..................~~..................~~.................**

**KX357114.1**  **..................~~..................~~.................**

**KX779520.1**  **..................~~..................~~.................**

**KX779521.1**  **..................~~..................~~.................**

**KX779522.1**  **..................~~..................~~.................**

**L48961.1**  **..................~~..................~~.................**

**L78128.1**  **..................~~..................~~.................**

**M18370.1**  **..................~~..................~~.................**

**M55506.1**  **..................~~..................~~.................**

**NC_001437.1**  **..................~~..................~~.................**

**JN644310.1**  **..................~~..................~~.................**

**U14163.1**  **..................~~..................~~.................**

**U15763.1**  **..................~~..................~~.................**

**U47032.1**  **..................~~..................~~.................**

**9) NS3 v2 assay**

Accession 5726 5884

Numbers: ....|....|....|....|....|....|....|....|....|....|....|....|....|

**GCAATGTGYCTCCAAAGAGC~~TCCTATGAYACAGAATAYCCAAA~~GCGAGCAGGGTCATCGAC**

**EF571853.1**  **....................~~.......................~~..................**

**AB051292.1**  **...........t........~~..t....................~~..............t...**

**AB196923.1**  **....................~~.......................~~..................**

**AB196924.1**  **....................~~.......................~~..................**

**AB196925.1**  **....................~~.......................~~..................**

**AB196926.1**  **....................~~.......................~~..................**

**AB241118.1**  **....................~~..a....................~~..............t...**

**AB241119.1**  **...........t........~~..t....................~~..............t...**

**AB269326.1**  **....................~~.......................~~..................**

**AB471666.2**  **...........t........~~..t....................~~..............t...**

**AB471667.2**  **...........t........~~..t....................~~..............t...**

**AB471668.2**  **...........t........~~..t....................~~..............t...**

**AB471669.2**  **...........t........~~..t....................~~..............t...**

**AB471670.2**  **...........t........~~..t....................~~..............t...**

**AB551990.1**  **....................~~.......................~~..................**

**AB551991.1**  **....................~~.......................~~..................**

**AB551992.1**  **....................~~.......................~~..................**

**AB569980.2**  **....................~~.......................~~..................**

**AB569981.2**  **....................~~.......................~~..................**

**AB569982.2**  **....................~~.......................~~..................**

**AB569983.2**  **....................~~.......................~~..................**

**AB569984.2**  **....................~~.......................~~..................**

**AB569985.2**  **....................~~.......................~~..................**

**AB569987.2**  **....................~~.......................~~..................**

**AB569988.2**  **....................~~.......................~~..................**

**AB569989.2**  **....................~~.......................~~..................**

**AB569990.2**  **....................~~.......................~~..................**

**AB594829.1**  **...........t........~~..t....................~~..............t...**

**AB698905.1**  **....................~~..a....................~~..............t...**

**AB698906.1**  **....................~~..a....................~~..............t...**

**AB698907.1**  **....................~~..a....................~~..a...........t...**

**AB698908.1**  **....................~~..a....................~~..a...........t...**

**AB698909.1**  **....................~~..a....................~~..............t...**

**AB830335.1**  **...........t........~~..t....................~~..............t...**

**AB853904.1**  **....................~~..a....................~~..a...........t...**

**AB920399.1**  **...........t........~~..t....................~~..............t...**

**AB981183.1**  **...........t........~~..t....................~~..............t...**

**AB981184.1**  **...........t........~~..t....................~~..............t...**

**AF014160.1**  **....................~~.......................~~..................**

**AF014161.1**  **....................~~.......................~~..................**

**AF045551.2**  **...........t........~~..t....................~~..............t...**

**AF069076.1**  **....................~~.......................~~..................**

**AF075723.1**  **....................~~.......................~~..................**

**AF080251.1**  **....................~~.......................~~..................**

**AF098735.1**  **....................~~.......................~~..................**

**AF098736.1**  **....................~~.......................~~..................**

**AF098737.1**  **....................~~.......................~~..................**

**AF217620.1**  **..............g.....~~..t....................~~.....t........t...**

**AF221499.1**  **....................~~.......................~~..............t...**

**AF221500.1**  **....................~~.......................~~..............t...**

**AF254452.1**  **....................~~.......................~~..................**

**AF254453.1**  **....................~~.......................~~..................**

**AF315119.1**  **....................~~.......................~~..................**

**AF416457.1**  **....................~~.......................~~..................**

**AF486638.1**  **....................~~.......................~~..................**

**AY184212.1**  **..tg.t........g.....~~..t........t...........~~...........g..t...**

**AY303791.1**  **....................~~.......................~~..................**

**AY303792.1**  **....................~~.......................~~..................**

**AY303793.1**  **....................~~.......................~~..............t...**

**AY303794.1**  **....................~~.......................~~..............t...**

**AY303795.1**  **....................~~.......................~~..............t...**

**AY303796.1**  **....................~~.......................~~..............t...**

**AY303797.1**  **....................~~.......................~~..............t...**

**AY303798.1**  **....................~~.......................~~..............t...**

**AY316157.1**  **...........t........~~..t....................~~..............t...**

**AY508812.1**  **....................~~.......................~~..................**

**AY508813.1**  **....................~~.......................~~..................**

**AY585242.1**  **....................~~.......................~~..................**

**AY585243.1**  **....................~~.......................~~..................**

**AY849939.1**  **....................~~.......................~~..................**

**D90194.1**  **....................~~.......................~~..................**

**D90195.1**  **....................~~.......................~~..................**

**DD298237.1**  **....................~~.......................~~..................**

**DD298243.1**  **....................~~.......................~~..................**

**DD367827.1**  **....................~~.......................~~..................**

**DD367828.1**  **....................~~.......................~~..................**

**DI075091.1**  **....................~~.......................~~..................**

**DI085220.1**  **....................~~.......................~~..................**

**EF107523.1**  **....................~~.......................~~..................**

**EF543861.1**  **....................~~.......................~~..................**

**EF623987.1**  **....................~~.......................~~..................**

**EF623988.1**  **....................~~.......................~~..................**

**EF623989.1**  **....................~~.......................~~..................**

**EU429297.1**  **...........t........~~..t....................~~..............t...**

**EU693899.1**  **...........t........~~..t....................~~..............t...**

**EU880214.1**  **...........t........~~..t....................~~..............t...**

**FJ185036.1**  **....................~~.......................~~..................**

**FJ185037.1**  **....................~~.......................~~..................**

**FJ495189.1**  **....................~~..a....................~~..............t...**

**GQ199609.1**  **....................~~.......................~~..................**

**GQ902058.1**  **...........t..g.....~~..t....................~~...........t..t...**

**GQ902059.1**  **...........t..g.....~~..t....................~~...........t..t...**

**GQ902060.1**  **...........t..g.....~~..t....................~~...........t..t...**

**GQ902061.1**  **...........t..g.....~~..t....................~~...........t..t...**

**GQ902062.1**  **...........t..g..g..~~..t....................~~...........t..t...**

**GQ902063.1**  **....................~~.......................~~..................**

**GQ918133.2**  **....................~~.......................~~..................**

**GU187972.1**  **....................~~..a....................~~..............t...**

**GU205163.1**  **....................~~..a....................~~..a...........t...**

**GU556217.1**  **..g.................~~..t....................~~..............t...**

**HE861351.1**  **....................~~.......................~~..................**

**HM228921.1**  **...........t........~~..t....................~~..............t...**

**HM366552.1**  **....................~~..a....................~~..............t...**

**HM596272.1**  **...g.....t.a..g.....~~...........c..g.....t..~~........a..g..t..t**

**HQ223285.1**  **..............g.....~~..t....................~~.....t........t..t**

**HQ223286.1**  **..............g.....~~..t....................~~.....t........t..t**

**HQ223287.1**  **..............g.....~~..t....................~~..............t..t**

**HQ652538.1**  **...........t........~~..t....................~~..............t...**

**HQ893545.1**  **...........t........~~..t....................~~........a.....t...**

**JF499788.1**  **...........t........~~..t....................~~..............t...**

**JF499789.1**  **...........t..g.....~~..t....................~~..............t...**

**JF499790.1**  **....................~~..a....................~~..............t...**

**JF706267.1**  **....................~~..a....................~~..............t...**

**JF706268.1**  **....................~~..a....................~~..............t...**

**JF706269.1**  **....................~~.......................~~..................**

**JF706270.1**  **...........t........~~..t....................~~..............t...**

**JF706271.1**  **...........t........~~..t....................~~..............t...**

**JF706272.1**  **....................~~.......................~~..................**

**JF706273.1**  **....................~~.......................~~..................**

**JF706274.1**  **....................~~..a....................~~..............t...**

**JF706275.1**  **....................~~.......................~~..................**

**JF706276.1**  **....................~~.......................~~..................**

**JF706277.1**  **....................~~..a....................~~..............t...**

**JF706278.1**  **...........t........~~..t....................~~..............t...**

**JF706279.1**  **..............g.....~~..t....................~~...........t..t...**

**JF706280.1**  **....................~~.......................~~..................**

**JF706281.1**  **...........t........~~..t....................~~..............t...**

**JF706282.1**  **...........t........~~..t....................~~..............t...**

**JF706283.1**  **....................~~.......................~~.....t............**

**JF706284.1**  **....................~~.......................~~..................**

**JF706285.1**  **....................~~.......................~~..................**

**JF706286.1**  **...........t........~~..t....................~~..............t..t**

**JF915894.1**  **..gg.......a..g.....~~..t........c..g.....c..~~........a..g.....t**

**JN381830.1**  **....................~~..a....................~~..............t...**

**JN381831.1**  **....................~~..a....................~~..............t...**

**JN381832.1**  **....................~~..a....................~~..............t..t**

**JN381833.1**  **....................~~..a....................~~..............t...**

**JN381834.1**  **....................~~..a....................~~..............t...**

**JN381835.1**  **....................~~..a....................~~..............t...**

**JN381836.1**  **..............g.....~~..a....................~~..............t...**

**JN381837.1**  **....................~~..a....................~~..............t...**

**JN381838.1**  **....................~~..a....................~~..............t...**

**JN381839.1**  **....................~~..a....................~~..............t...**

**JN381840.1**  **....................~~..a....................~~..............t...**

**JN381841.1**  **....................~~..a....................~~..............t...**

**JN381842.1**  **....................~~..a....................~~..............t...**

**JN381843.1**  **....................~~..a....................~~..............t...**

**JN381844.1**  **....................~~..a....................~~..............t...**

**JN381845.1**  **....................~~..a....................~~..............t...**

**JN381846.1**  **...........t........~~..t....................~~..............t...**

**JN381847.1**  **...........t........~~..t....................~~..............t...**

**JN381848.1**  **...........t........~~..t....................~~..............t...**

**JN381849.1**  **...........t........~~..t....................~~..............t...**

**JN381850.1**  **...........t........~~..t....................~~..............t...**

**JN381851.1**  **...........t........~~..t....................~~..............t...**

**JN381852.1**  **...........t........~~..t....................~~..............t...**

**JN381853.1**  **....................~~.......................~~..................**

**JN381854.1**  **....................~~.......................~~..................**

**JN381855.1**  **....................~~.......................~~..................**

**JN381856.1**  **....................~~.......................~~..................**

**JN381857.1**  **....................~~.......................~~..................**

**JN381858.1**  **....................~~.......................~~..................**

**JN381859.1**  **....................~~.......................~~..................**

**JN381860.1**  **....................~~.......................~~..................**

**JN381861.1**  **....................~~.......................~~..................**

**JN381862.1**  **....................~~.......................~~..................**

**JN381863.1**  **....................~~.......................~~..................**

**JN381864.1**  **....................~~.......................~~..................**

**JN381865.1**  **....................~~.......................~~..................**

**JN381866.1**  **....................~~.......................~~..................**

**JN381867.1**  **....................~~.......................~~..................**

**JN381868.1**  **....................~~.......................~~..................**

**JN381869.1**  **....................~~.......................~~..................**

**JN381870.1**  **....................~~.......................~~..................**

**JN381871.1**  **....................~~.......................~~..................**

**JN381872.1**  **....................~~.......................~~..................**

**JN381873.1**  **....................~~.......................~~..................**

**JN604986.1**  **....................~~.......................~~..................**

**JN711458.1**  **....................~~.......................~~..................**

**JN711459.1**  **....................~~.......................~~..................**

**JN864064.1**  **....................~~.......................~~..................**

**JQ031753.1**  **....................~~..a....................~~..............t...**

**JQ086762.1**  **....................~~.......................~~..................**

**JQ086763.1**  **....................~~.......................~~..................**

**JX050179.1**  **....................~~.......................~~..................**

**JX072965.1**  **....................~~.......................~~..................**

**JX131374.1**  **....................~~.......................~~..................**

**KC183732.1**  **....................~~.......................~~..................**

**KC196115.1**  **....................~~..a....................~~..............t...**

**KC517497.1**  **....................~~.......................~~..................**

**KC915016.1**  **....................~~.......................~~..............t...**

**KF297915.1**  **....................~~.......................~~..................**

**KF297916.1**  **....................~~.......................~~..............t...**

**KF667310.1**  **....................~~.......................~~..............t...**

**KF667311.1**  **....................~~.......................~~..............t...**

**KF667312.1**  **....................~~.......................~~..................**

**KF667313.1**  **....................~~.......................~~..................**

**KF667314.1**  **....................~~.......................~~..............t...**

**KF667315.1**  **....................~~.......................~~..............t...**

**KF667316.1**  **...........t........~~..t....................~~..............t...**

**KF667317.1**  **...........t........~~..t....................~~..............t...**

**KF667318.1**  **....................~~..a....................~~..............t...**

**KF667319.1**  **....................~~..a....................~~..............t...**

**KF667320.1**  **....................~~..a....................~~..............t...**

**KF667321.1**  **...........t........~~..t....................~~..................**

**KF667322.1**  **...........t........~~..t....................~~..............t...**

**KF667323.1**  **....................~~..a...........g........~~......c.......t...**

**KF667324.1**  **....................~~..a...........g........~~......c.......t...**

**KF667325.1**  **...........t........~~..t....................~~..............t...**

**KF667326.1**  **....................~~..a...........g........~~......c.......t...**

**KF667327.1**  **....................~~..a....................~~..............t...**

**KF711994.1**  **....................~~.......................~~..................**

**KF907505.1**  **....................~~.......................~~..................**

**KM658163.1**  **...........t........~~..t....................~~..............t...**

**KM677246.1**  **...g.....t.a..g.....~~..t........c..g.....t..~~........a..g..t..t**

**KP164498.2**  **....................~~.......................~~..................**

**KR265316.1**  **....................~~.......................~~..................**

**KT229572.1**  **....................~~..a....................~~..............t...**

**KT229573.1**  **....................~~..a....................~~..............t...**

**KT229574.1**  **....................~~..a....................~~..............t...**

**KT229575.1**  **....................~~..a....................~~..............t...**

**KT239164.1**  **....................~~.......................~~..................**

**KT957419.1**  **....................~~..a....................~~..............t...**

**KT957420.1**  **...........t........~~..t....................~~..............t...**

**KT957421.1**  **.........a..........~~..a....................~~..............t...**

**KT957422.1**  **..............g.....~~..t....................~~...........t..t...**

**KT957423.1**  **..............g.....~~..t....................~~...........t..t...**

**KU323483.1**  **....................~~.......................~~..................**

**KU351667.1**  **....................~~..a....................~~..............t...**

**KU351668.1**  **...........t........~~..t....................~~..............t...**

**KU363309.1**  **....................~~.......................~~..................**

**KU508408.1**  **...........t........~~..t....................~~..............t...**

**KU508409.1**  **...........t........~~..t....................~~..............t...**

**KU821122.1**  **....................~~.......................~~..................**

**KU871316.1**  **....................~~.......................~~..................**

**KU871317.1**  **....................~~.......................~~..................**

**KU871318.1**  **....................~~.......................~~..................**

**KU871319.1**  **....................~~.......................~~..................**

**KU871320.1**  **....................~~.......................~~..................**

**KU871321.1**  **....................~~.......................~~..................**

**KU871322.1**  **....................~~.......................~~..................**

**KU871323.1**  **....................~~.......................~~..................**

**KU871324.1**  **....................~~.......................~~..................**

**KU871325.1**  **....................~~.......................~~..................**

**KU871326.1**  **....................~~.......................~~..................**

**KU871327.1**  **....................~~.......................~~..................**

**KU871328.1**  **....................~~.......................~~..................**

**KU871329.1**  **....................~~.......................~~..................**

**KU871330.1**  **....................~~.......................~~..................**

**KU871331.1**  **....................~~.......................~~..................**

**KU871332.1**  **....................~~.......................~~..................**

**KU871333.1**  **....................~~.......................~~..................**

**KU871334.1**  **....................~~.......................~~..................**

**KU871335.1**  **....................~~.......................~~..................**

**KU871336.1**  **....................~~.......................~~..................**

**KU871337.1**  **....................~~.......................~~..................**

**KU871338.1**  **....................~~.......................~~..................**

**KU871339.1**  **....................~~.......................~~..................**

**KU871340.1**  **....................~~.......................~~..................**

**KU871341.1**  **....................~~.......................~~..................**

**KU871342.1**  **....................~~.......................~~..................**

**KU871343.1**  **....................~~.......................~~..................**

**KU871344.1**  **....................~~.......................~~..................**

**KU871345.1**  **....................~~.......................~~..................**

**KU871346.1**  **....................~~.......................~~..................**

**KU871347.1**  **....................~~.......................~~..................**

**KU871348.1**  **....................~~.......................~~..................**

**KU871349.1**  **....................~~.......................~~..................**

**KU871350.1**  **....................~~.......................~~..................**

**KU871351.1**  **....................~~.......................~~..................**

**KU871352.1**  **....................~~.......................~~..................**

**KU871353.1**  **....................~~.......................~~..................**

**KU871354.1**  **....................~~.......................~~..................**

**KU871355.1**  **....................~~.......................~~..................**

**KU871356.1**  **....................~~.......................~~..................**

**KU871357.1**  **....................~~.......................~~..................**

**KU871358.1**  **....................~~.......................~~..................**

**KU871359.1**  **....................~~.......................~~..................**

**KU871360.1**  **....................~~.......................~~..................**

**KU871361.1**  **....................~~.......................~~..................**

**KU871362.1**  **....................~~.......................~~..................**

**KU871363.1**  **....................~~.......................~~..................**

**KU871364.1**  **....................~~.......................~~..................**

**KU871365.1**  **....................~~.......................~~..................**

**KU871366.1**  **....................~~.......................~~..................**

**KU871367.1**  **....................~~.......................~~..................**

**KU871368.1**  **....................~~.......................~~..................**

**KU871369.1**  **....................~~.......................~~..................**

**KU871370.1**  **....................~~.......................~~..................**

**KU871371.1**  **....................~~.......................~~..................**

**KX357114.1**  **...........t........~~..t....................~~..............t...**

**KX779520.1**  **....................~~.......................~~..................**

**KX779521.1**  **....................~~.......................~~..................**

**KX779522.1**  **....................~~.......................~~..................**

**L48961.1**  **....................~~.......................~~..................**

**L78128.1**  **....................~~.......................~~..................**

**M18370.1**  **....................~~.......................~~..................**

**M55506.1**  **....................~~.......................~~..................**

**NC_001437.1**  **....................~~.......................~~..................**

**JN644310.1**  **....................~~.......................~~..................**

**U14163.1**  **....................~~.......................~~..................**

**U15763.1**  **....................~~.......................~~..................**

**U47032.1**  **....................~~.......................~~..................**

Note that NS2A v2 (using the reverse complement of the probe for v1), NS5 v1 and NS3 v1 are not shown in this table as they are the same or very similar to their counterpart versions in the table.

**Table E: Cq Results for** **Comparison of RT-qPCR conditions using the Pre-existing In-house JEV RT-qPCR assay**

| **Primers and Probes** | **Mastermix** | **Sample vol.** | **Reaction vol.** | **10^-2^** | **10^-2^** | **10^-2^** | **10^-2^** | **10^-3^** | **10^-3^** | **10^-3^** | **10^-3^** | **10^-4^** | **10^-4^** | **10^-4^** | **10^-4^** | **10^-5^** | **10^-5^** | **10^-5^** | **10^-5^** | **10^-6^** | **10^-6^** | **10^-6^** | **10^-6^** | **10^-7^** | **10^-7^** | **10^-7^** | **10^-7^** | **10^-8^** | **10^-8^** | **10^-8^** | **10^-8^** | **10^-9^** | **10^-9^** | **10^-9^** | **10^-9^** |
| --- | --- | --- | --- | --- | --- | --- | --- | --- | --- | --- | --- | --- | --- | --- | --- | --- | --- | --- | --- | --- | --- | --- | --- | --- | --- | --- | --- | --- | --- | --- | --- | --- | --- | --- | --- |
|  |  |  |  | **Cq1** | **Cq2** | **Cq3** | **Mean** | **Cq1** | **Cq2** | **Cq3** | **Mean** | **Cq1** | **Cq2** | **Cq3** | **Mean** | **Cq1** | **Cq2** | **Cq3** | **Mean** | **Cq1** | **Cq2** | **Cq3** | **Mean** | **Cq1** | **Cq2** | **Cq3** | **Mean** | **Cq1** | **Cq2** | **Cq3** | **Mean** | **Cq1** | **Cq2** | **Cq3** | **Mean** |
| **Mastermix** | | | |  |  |  |  |  |  |  |  |  |  |  |  |  |  |  |  |  |  |  |  |  |  |  |  |  |  |  |  |  |  |  |  |
| In-house^1^ | Superscript-III | 5µl | 25µl |  |  |  |  |  |  |  |  | 26.27 | 26.33 |  | 26.30 | 29.22 | 29.34 |  | 29.28 | 32.90 | 33.73 |  | 33.32 | No Cq | No Cq |  | No Cq |  |  |  |  |  |  |  |  |
| In-house^1^ | Express | 5µl | 25µl |  |  |  |  |  |  |  |  | 24.89 | 25.17 |  | 25.03 | 29.16 | 28.24 |  | 28.70 | >40 | 35.30 |  | 39.50 | No Cq | No Cq |  | No Cq |  |  |  |  |  |  |  |  |
| In-house^2^ | Fastvirus | 5µl | 25µl |  |  |  |  |  |  |  |  | 25.46 | 24.84 |  | 25.15 | 28.45 | 28.62 |  | 28.53 |  | 35.00 |  | 35.00 | No Cq | No Cq |  | No Cq |  |  |  |  |  |  |  |  |
|  |  |  |  |  |  |  |  |  |  |  |  |  |  |  |  |  |  |  |  |  |  |  |  |  |  |  |  |  |  |  |  |  |  |  |  |
| **Reaction volume** | | | |  |  |  |  |  |  |  |  |  |  |  |  |  |  |  |  |  |  |  |  |  |  |  |  |  |  |  |  |  |  |  |  |
| In-house^5^ | Superscript-III | 5µl | 25µl | 18.97 | 19.87 |  | 19.42 | 23.81 | 23.35 |  | 23.58 | 25.65 | 25.55 |  | 25.60 | 29.15 | 29.27 |  | 29.21 | 34.28 | 31.76 |  | 33.02 | >40 | 39.94 |  | >40 | No Cq | No Cq |  | No Cq | No Cq | No Cq |  | No Cq |
| In-house^6^ | Superscript-III | 10µl | 50µl | 18.42 | 17.25 |  | 17.84 | 20.42 | 20.11 |  | 20.27 | 23.27 | 23.03 |  | 23.15 | 26.14 | 27.99 |  | 27.06 | 28.95 | 28.80 |  | 28.88 | 39.71 | 38.99 |  | 39.35 | >40 | >40 |  | >40 | >40 | >40 |  | >40 |
|  |  |  |  |  |  |  |  |  |  |  |  |  |  |  |  |  |  |  |  |  |  |  |  |  |  |  |  |  |  |  |  |  |  |  |  |
| **Sample volume** | | | |  |  |  |  |  |  |  |  |  |  |  |  |  |  |  |  |  |  |  |  |  |  |  |  |  |  |  |  |  |  |  |  |
| In-house^3^ | Superscript-III | 10µl | 50µl | 18.77 | 18.58 | 19.06 | 18.80 | 22.37 | 22.38 | 22.40 | 22.39 | 25.93 | 26.15 | 25.59 | 25.89 | 29.26 | 28.59 | 28.90 | 28.92 | 32.31 | 32.97 | 32.74 | 32.67 | 35.96 | 41.30 | 36.17 | 37.81 | >40 | >40 | >40 | >40 | No Cq | >40 | No Cq | No Cq |
| In-house^3^ | Superscript-III | 16µl | 50µl | 18.15 | 18.08 | 18.17 | 18.13 | 21.15 | 21.49 | 21.50 | 21.38 | 25.16 | 24.84 | 25.17 | 25.06 | 28.77 | 27.60 | 28.27 | 28.22 | 30.76 | 31.79 | 25.71 | 29.42 | 35.46 | 36.88 | 37.29 | 36.55 | No Cq | >40 | No Cq | No Cq | No Cq | No Cq | No Cq | No Cq |
| In-house^4^ | Fastvirus | 5µl | 25µl |  |  |  |  |  |  |  |  | 25.46 | 24.84 |  | 25.15 | 28.45 | 28.62 |  | 28.53 |  | 35.00 |  | 35.00 | No Cq | No Cq |  | No Cq | No Cq | No Cq |  | No Cq |  |  |  |  |
| In-house^4^ | Fastvirus | 15µl | 25µl |  |  |  |  |  |  |  |  | 24.29 | 24.08 |  | 24.18 | 27.53 | 27.65 |  | 27.59 | 30.47 | 30.91 |  | 30.69 | 34.88 | 34.84 |  | 34.86 | >40 | >40 |  | >40 |  |  |  |  |

Numbers in superscript denote the RT-qPCR run in which the experiments were performed. All experiments were performed with JEV strain G1-1326 RNA at 10^-2^ to 10^-9^ dilutions. Blank cells were not performed.

**Table F: Cq Results for the Selection of the Best Performing RT-qPCR systems** **Using Superscript-III kit with Standard Conditions**

| **PCR System** | ***JEV Strain** | **10^-2^** | **10^-2^** | **10^-2^** | **10^-3^** | **10^-3^** | **10^-3^** | **10^-4^** | **10^-4^** | **10^-4^** | **10^-5^** | **10^-5^** | **10^-5^** | **10^-6^** | **10^-6^** | **10^-6^** | **10^-7^** | **10^-7^** | **10^-7^** | **10^-8^** | **10^-8^** | **10^-8^** | **10^-9^** | **10^-9^** | **10^-9^** | **10^-10^** | **10^-10^** | **10^-10^** | **10^-11^** | **10^-11^** | **10^-11^** |
| --- | --- | --- | --- | --- | --- | --- | --- | --- | --- | --- | --- | --- | --- | --- | --- | --- | --- | --- | --- | --- | --- | --- | --- | --- | --- | --- | --- | --- | --- | --- | --- |
|  |  | **Cq1** | **Cq2** | **Mean** | **Cq1** | **Cq2** | **Mean** | **Cq1** | **Cq2** | **Mean** | **Cq1** | **Cq2** | **Mean** | **Cq1** | **Cq2** | **Mean** | **Cq1** | **Cq2** | **Mean** | **Cq1** | **Cq2** | **Mean** | **Cq1** | **Cq2** | **Mean** | **Cq1** | **Cq2** | **Mean** | **Cq1** | **Cq2** | **Mean** |
| In-house^1^ | G1 | 21.02 | 20.69 | 20.86 | 24.08 | 23.99 | 24.03 | 27.46 | 27.59 | 27.53 | 30.27 | 30.44 | 30.36 | 37.94 | 38.07 | 38.00 | No Cq | >40 | No Cq | No Cq | No Cq | No Cq |  |  |  |  |  |  |  |  |  |
| Pyke^1^ | G1 | 16.25 | 16.22 | 16.23 | 19.23 | 19.55 | 19.39 | 22.85 | 22.90 | 22.87 | 26.24 | 26.32 | 26.28 | 29.32 | 29.54 | 29.43 | 31.98 | 32.79 | 32.38 | 35.54 | 35.43 | 35.48 |  |  |  |  |  |  |  |  |  |
| Yang^1^ | G1 | 18.58 | 18.65 | 18.62 | 21.81 | 21.63 | 21.72 | 25.29 | 25.37 | 25.33 | 28.04 | 27.70 | 27.87 | 31.34 | 31.32 | 31.33 | >40 | >40 | >40 | No Cq | No Cq | No Cq |  |  |  |  |  |  |  |  |  |
| Shirato^1^ | G1 | 26.07 | 25.74 | 25.90 | 29.61 | 29.38 | 29.50 | 33.20 | 32.89 | 33.05 | 36.20 | 36.19 | 36.20 | >40 | >40 | >40 | No Cq | No Cq | No Cq | No Cq | No Cq | No Cq |  |  |  |  |  |  |  |  |  |
| In-house^1^ | G1 | 20.44 | 20.61 | 20.52 | 23.76 | 23.64 | 23.70 | 26.97 | 26.77 | 26.87 | 30.81 | 30.13 | 30.47 | 34.08 | 33.49 | 33.79 | >40 | >40 | >40 | No Cq | No Cq | No Cq |  |  |  |  |  |  |  |  |  |
| NS2A v1^2^ | G1 | 16.92 | 16.69 | 16.81 | 20.14 | 20.29 | 20.21 | 23.31 | 23.34 | 23.33 | 26.71 | 26.73 | 26.72 | 30.15 | 29.75 | 29.95 | 34.12 | 33.38 | 33.75 | >40 | No Cq | No Cq |  |  |  |  |  |  |  |  |  |
| NS2A v2^2^ | G1 | 17.24 | 17.19 | 17.22 | 20.37 | 20.58 | 20.48 | 23.63 | 23.79 | 23.71 | 27.29 | 27.14 | 27.21 | 30.38 | 30.69 | 30.54 | 33.45 | 34.38 | 33.91 | No Cq | No Cq | No Cq |  |  |  |  |  |  |  |  |  |
| NS5 v1^2^ | G1 | 19.04 | 18.95 | 19.00 | 22.41 | 22.61 | 22.51 | 25.76 | 25.65 | 25.71 | 29.35 | 28.80 | 29.08 | 32.65 | 32.12 | 32.39 | >40 | No Cq | No Cq | No Cq | No Cq | No Cq |  |  |  |  |  |  |  |  |  |
| NS5 v2^3^ | G1 | 19.08 | 19.87 | 19.48 | 23.22 | 23.68 | 23.45 | 26.72 | 27.86 | 27.29 | 30.35 | 38.32 | 34.34 | 34.30 | 38.40 | 36.35 | No Cq | No Cq | No Cq | No Cq | No Cq | No Cq |  |  |  |  |  |  |  |  |  |
| NS3 v1^3^ | G1 | 21.51 | 21.71 | 21.61 | 24.56 | 24.64 | 24.60 | 28.04 | 29.02 | 28.53 | 31.51 | 30.97 | 31.24 | 33.75 | 39.50 | 36.63 | >40 | 39.82 | >40 | >40 | No Cq | No Cq |  |  |  |  |  |  |  |  |  |
| NS3 v2^3^ | G1 | 21.70 | 22.06 | 21.88 | 24.46 | 24.30 | 24.38 | 27.36 | 27.06 | 27.21 | 30.61 | 31.57 | 31.09 | 32.96 | 34.34 | 33.65 | No Cq | >40 | No Cq | No Cq | No Cq | No Cq |  |  |  |  |  |  |  |  |  |
| In-house^4^ | G3 |  |  |  |  |  |  |  |  |  | 30.78 | 31.59 | 31.18 | 33.95 | 35.32 | 34.64 | >40 | >40 | >40 | No Cq | No Cq | No Cq | No Cq | No Cq | No Cq | No Cq | No Cq | No Cq | No Cq | No Cq | No Cq |
| Pyke^4^ | G3 |  |  |  |  |  |  |  |  |  | 27.88 | 27.45 | 27.67 | 30.93 | 30.71 | 30.82 | 34.07 | 33.64 | 33.86 | 36.47 | 35.85 | 36.16 | No Cq | 33.80 | No Cq | No Cq | No Cq | No Cq | No Cq | No Cq | No Cq |
| Yang^4^ | G3 |  |  |  |  |  |  |  |  |  | 27.88 | 29.20 | 28.54 | 30.67 | 30.39 | 30.53 | 34.16 | 33.23 | 33.69 | No Cq | >40 | No Cq | No Cq | No Cq | No Cq | No Cq | No Cq | No Cq | No Cq | No Cq | No Cq |
| Shirato^4^ | G3 |  |  |  |  |  |  |  |  |  | 33.87 | 35.11 | 34.49 | 37.61 | No Cq | No Cq | No Cq | No Cq | No Cq | No Cq | No Cq | No Cq | No Cq | No Cq | No Cq | No Cq | No Cq | No Cq | No Cq | No Cq | No Cq |
| In-house^5^ | G3 |  |  |  |  |  |  |  |  |  | 30.4 |  | 30.4 | 34.7 |  | 34.7 | No Cq |  | No Cq | No Cq |  | No Cq | No Cq |  | No Cq | No Cq |  | No Cq | No Cq |  | No Cq |
| NS2A v1^5^ | G3 |  |  |  |  |  |  |  |  |  | 27.3 |  | 27.3 | 29.7 |  | 29.7 | 33.9 |  | 33.9 | No Cq |  | No Cq | No Cq |  | No Cq | No Cq |  | No Cq | No Cq |  | No Cq |
| NS2A v2^5^ | G3 |  |  |  |  |  |  |  |  |  | 27.1 |  | 27.1 | 30.1 |  | 30.1 | 34.2 |  | 34.2 | No Cq |  | No Cq | No Cq |  | No Cq | No Cq |  | No Cq | No Cq |  | No Cq |
| NS5 v1^5^ | G3 |  |  |  |  |  |  |  |  |  | 26.7 |  | 26.7 | 30.1 |  | 30.1 | 32.6 |  | 32.6 | No Cq |  | No Cq | No Cq |  | No Cq | No Cq |  | No Cq | No Cq |  | No Cq |
| NS5 v2^6^ | G3 |  |  |  |  |  |  |  |  |  | 27.3 |  | 27.3 | 29.5 |  | 29.5 | 32.6 |  | 32.6 | No Cq |  | No Cq | No Cq |  | No Cq | No Cq |  | No Cq | No Cq |  | No Cq |
| NS3 v1^6^ | G3 |  |  |  |  |  |  |  |  |  | 28.0 |  | 28.0 | 31.2 |  | 31.2 | 33.7 |  | 33.7 | 35.6 |  | 35.6 | No Cq |  | No Cq | No Cq |  | No Cq | No Cq |  | No Cq |
| NS3 v2^6^ | G3 |  |  |  |  |  |  |  |  |  | 27.5 |  | 27.5 | 31.3 |  | 31.3 | 33.6 |  | 33.6 | 35.2 |  | 35.2 | No Cq |  | No Cq | No Cq |  | No Cq | No Cq |  | No Cq |

Numbers in superscript denote the RT-qPCR run in which the experiments were performed. *JEV strains G1-1326 and G3-RP9-190 RNA were used at 10^-2^ to 10^-8^ and 10^-5^ to 10^-11^ dilutions respectively.

**Table G: Cq Results for Annealing Temperature Optimisation using the Pre-existing In-house JEV RT-qPCR assay**

| **PCR System** | **Annealing Temperature (°C)** | **10^-5^** | **10^-5^** | **10^-5^** | **10^-6^** | **10^-6^** | **10^-6^** | **10^-7^** | **10^-7^** | **10^-7^** | **10^-8^** | **10^-8^** | **10^-8^** | **10^-9^** | **10^-9^** | **10^-9^** |
| --- | --- | --- | --- | --- | --- | --- | --- | --- | --- | --- | --- | --- | --- | --- | --- | --- |
|  |  | **Cq1** | **Cq2** | **Mean** | **Cq1** | **Cq2** | **Mean** | **Cq1** | **Cq2** | **Mean** | **Cq1** | **Cq2** | **Mean** | **Cq1** | **Cq2** | **Mean** |
| Pyke | 64 | 28.42 | 28.54 | 28.48 | 31.50 | 32.22 | 31.86 | 34.09 | 34.51 | 34.30 | 37.70 | 35.53 | 36.61 | No Cq | No Cq | No Cq |
| Pyke | 62 | 27.65 | 27.69 | 27.67 | 30.63 | 30.85 | 30.74 | 33.67 | 34.49 | 34.08 | >40 | 38.03 | >40 | No Cq | No Cq | No Cq |
| Pyke | 60 | 27.48 | 27.72 | 27.60 | 30.49 | 30.79 | 30.64 | 34.01 | 34.26 | 34.14 | 36.88 | 37.90 | 37.39 | No Cq | No Cq | No Cq |
| Pyke | 58 | 27.41 | 27.36 | 27.39 | 30.90 | 30.36 | 30.63 | 33.89 | 33.70 | 33.80 | 37.15 | 37.77 | 37.46 | No Cq | No Cq | No Cq |
| Pyke | 56 | 27.35 | 27.46 | 27.41 | 30.93 | 30.58 | 30.76 | 33.63 | 33.86 | 33.75 | 36.06 | 34.79 | 35.43 | No Cq | No Cq | No Cq |
| Pyke | 54 | 27.54 | 27.61 | 27.58 | 30.79 | 30.97 | 30.88 | 33.69 | 35.18 | 34.44 | >40 | 36.36 | >40 | No Cq | No Cq | No Cq |
| Pyke | 52 | 28.79 | 28.76 | 28.78 | 32.35 | 31.76 | 32.05 | 36.75 | 35.16 | 35.95 | >40 | >40 | No Cq | No Cq | No Cq | No Cq |
| NS2Av1 | 64 | 29.16 | 28.69 | 28.92 | 32.70 | 32.46 | 32.58 | 35.10 | 36.60 | 35.85 | No Cq | >40 | No Cq | No Cq | No Cq | No Cq |
| NS2Av1 | 62 | 28.28 | 28.48 | 28.38 | 31.75 | 32.35 | 32.05 | 34.94 | 34.33 | 34.63 | 38.61 | 39.37 | 38.99 | No Cq | No Cq | No Cq |
| NS2Av1 | 60 | 28.09 | 28.13 | 28.11 | 32.18 | 31.30 | 31.74 | 35.34 | 35.75 | 35.55 | No Cq | No Cq | No Cq | No Cq | No Cq | No Cq |
| NS2Av1 | 58 | 28.06 | 28.21 | 28.14 | 31.25 | 31.20 | 31.23 | >40 | 35.56 | >40 | No Cq | No Cq | No Cq | No Cq | No Cq | No Cq |
| NS2Av1 | 56 | 28.10 | 28.15 | 28.13 | 31.39 | 32.17 | 31.78 | No Cq | No Cq | No Cq | No Cq | No Cq | No Cq | No Cq | No Cq | No Cq |
| NS2Av1 | 54 | 28.25 | 28.58 | 28.42 | 31.55 | 31.93 | 31.74 | >40 | >40 | >40 | No Cq | No Cq | No Cq | No Cq | No Cq | No Cq |
| NS2Av1 | 52 | 29.88 | 29.88 | 29.88 | 34.16 | 34.25 | 34.20 | No Cq | No Cq | No Cq | No Cq | No Cq | No Cq | No Cq | No Cq | No Cq |
| NS3v2 | 62 | 33.37 | 33.22 | 33.30 | >40 | >40 | No Cq | No Cq | >40 | No Cq | No Cq | No Cq | No Cq | No Cq | No Cq | No Cq |
| NS3v2 | 60 | 31.32 | 31.10 | 31.21 | 34.57 | 34.05 | 34.31 | >40 | >40 | >40 | No Cq | No Cq | No Cq | No Cq | No Cq | No Cq |
| NS3v2 | 58 | 31.55 | 31.08 | 31.32 | 34.06 | 35.02 | 34.54 | 37.23 | 36.42 | 36.83 | No Cq | No Cq | No Cq | No Cq | No Cq | No Cq |
| NS3v2 | 56 | 31.55 | 31.08 | 31.32 | 34.06 | 35.02 | 34.54 | 37.23 | 36.42 | 36.83 | No Cq | No Cq | No Cq | No Cq | No Cq | No Cq |
| NS3v2 | 54 | 30.28 | 29.91 | 30.10 | 33.03 | 33.11 | 33.07 | 35.68 | 36.23 | 35.96 | 36.65 | No Cq | No Cq | No Cq | No Cq | No Cq |
| NS3v2 | 52 | 31.47 | 30.78 | 31.12 | 34.28 | 34.75 | 34.52 | 37.08 | 36.15 | 36.62 | No Cq | No Cq | No Cq | No Cq | >40 | No Cq |

Note that the different annealing temperatures were performed on different plates, and ct values between temperatures are not perfectly comparable. JEV G1-1326 RNA was used at 10^-5^ to 10^-9^ dilutions.

**Table H: Cq Results for Primer Concentration Optimisation Experiments Using Superscript-III kit with Standard Conditions**

| **Pyke** | **Forward** | | | | | | |
| --- | --- | --- | --- | --- | --- | --- | --- |
| **Reverse** |  | **200nM** | **300nM** | **400nM** | **500nM** | **600nM** | **800nM** |
|  | **200nM** | 27.0 | 26.8 | 26.7 | 26.8 | 27.0 | - |
|  | **300nM** | 27.1 | 26.8 | 29.0 | 26.7 | 26.6 | - |
|  | **400nM** | 26.9 | 26.8 | 26.8 | 26.5 | 26.9 | - |
|  | **500nM** | 27.1 | 26.5 | 26.5 | 26.6 | 26.7 | - |
|  | **600nM** | 27.0 | 26.6 | 26.6 | 26.7 | 26.8 | - |
|  | **800nM** | - | - | - | - | - | - |
|  | | | | | | | |
| **NS2A** | **Forward** | | | | | | |
| **Reverse** |  | **200nM** | **300nM** | **400nM** | **500nM** | **600nM** | **800nM** |
|  | **200nM** | 27.6 | 27.0 | 27.3 | - | - | - |
|  | **300nM** | **-** | 27.0 | 27.4 | 26.7 | 27.4 | 27.3 |
|  | **400nM** | 27.0 | 26.9 | 27.3 | 26.9 | 27.3 | 27.3 |
|  | **500nM** | **-** | 27.1 | 27.2 | 27.1 | 27.2 | 26.5 |
|  | **600nM** | **-** | 27.1 | 26.7 | 27.4 | 26.9 | 27.1 |
|  | **800nM** | **-** | 26.7 | 26.7 | 26.6 | 27.2 | 26.8 |
|  |  |  |  |  |  |  |  |
|  | | | | | | | |
| **NS3** | **Forward** | | | | | | |
| **Reverse** |  | **200nM** | **300nM** | **400nM** | **500nM** | **600nM** | **800nM** |
|  | **200nM** | 30.5 | 30.1 | 30.2 | 30.2 | 29.7 | - |
|  | **300nM** | 31.5 | 29.9 | 29.9 | 29.6 | 29.5 | - |
|  | **400nM** | 29.5 | 29.8 | 29.7 | 30.0 | 29.3 | - |
|  | **500nM** | 29.8 | 29.4 | 30.0 | 29.6 | 29.2 | - |
|  | **600nM** | 0.0 | 30.0 | 29.6 | 29.6 | 29.1 | - |
|  | **800nM** | - | - | - | - | - | - |

Mean Cq results for duplicates are presented. G1-1326 RNA at 10^-6^ dilution was used.

**Table I: Cq Results for Probe Concentrations Optimisation Experiments Using Superscript-III kit with Standard Conditions**

|  | **100nM** | | **200nM** | | **300nM** | | **400nM** | |
| --- | --- | --- | --- | --- | --- | --- | --- | --- |
| **Pyke** | 31.3 | 31.1 | 30.1 | 29.6 | 29.7 | 29.2 | 29.5 | 29.5 |
| **NS2A** | 29.5 | 29.4 | 29.1 | 29.4 | 29.2 | 29.4 | 29.9 | 29.6 |
| **NS3** | 32.8 | 31.8 | 32.0 | 31.6 | 31.5 | 30.4 | 34.3 | 33.0 |

Mean Cq results for duplicates are presented. G1-1326 RNA at 10^-6^ dilution was used.
